# Circulating miR-132-3p as a Candidate Diagnostic Biomarker for Malignant Mesothelioma

**DOI:** 10.1155/2017/9280170

**Published:** 2017-02-21

**Authors:** Daniel G. Weber, Katarzyna Gawrych, Swaantje Casjens, Alexander Brik, Martin Lehnert, Dirk Taeger, Beate Pesch, Jens Kollmeier, Torsten T. Bauer, Georg Johnen, Thomas Brüning

**Affiliations:** ^1^Institute of Prevention and Occupational Medicine of the German Social Accident Insurance-Institute of the Ruhr-Universität Bochum (IPA), Buerkle-de-la-Camp-Platz 1, 44789 Bochum, Germany; ^2^Lungenklinik Heckeshorn, HELIOS Klinikum Emil von Behring, Walterhöferstraße 11, 14165 Berlin, Germany

## Abstract

The use of circulating microRNAs as biomarkers has opened new opportunities for diagnosis of cancer because microRNAs exhibit tumor-specific expression profiles. The aim of this study was the identification of circulating microRNAs in human plasma as potential biomarkers for the diagnosis of malignant mesothelioma. For discovery, TaqMan Low Density Array Human MicroRNA Cards were used to analyze 377 microRNAs in plasma samples from 21 mesothelioma patients and 21 asbestos-exposed controls. For verification, individual TaqMan microRNA assays were used for quantitative real-time PCR in plasma samples from 22 mesothelioma patients and 44 asbestos-exposed controls. The circulating miR-132-3p showed different expression levels between mesothelioma patients and asbestos-exposed controls. For discrimination, sensitivity of 86% and specificity of 61% were calculated. Circulating miR-132-3p in plasma was not affected by hemolysis and no impact of age or smoking status on miR-132-3p levels could be observed. For the combination of miR-132-3p with the previously described miR-126, sensitivity of 77% and specificity of 86% were calculated. The results of this study indicate that miR-132-3p might be a new promising diagnostic biomarker for malignant mesothelioma. It is indicated that the combination of miR-132-3p with other individual biomarkers improves the biomarker performance.

## 1. Introduction

Asbestos-related diseases are a global public health problem. World Health Organization (WHO) estimated 107,000 deaths annually worldwide related to asbestosis, lung cancer, and mesothelioma [1]. The majority of the asbestos-related disease burden occurs in Europe with more than 71,000 mesothelioma deaths from 1994 to 2012 [2]. In Germany alone, more than 12,000 deaths were recorded between 2000 and 2010 [3]. Between 2001 and 2050, approximately 65,000 deaths are calculated for Great Britain [4] and similar trends are estimated worldwide, for example, 66,000 cases in Japan until 2050 [5] and 85,000 cases in the USA until 2054 [6]. Thus, asbestos-related diseases, particularly mesothelioma, still remain a major public health problem in the future.

For the detection of cancer, the analysis of biomarkers in various body fluids is promising. Biomarkers are economical and easy to apply and might be simply implemented in clinical routine in order to detect the disease directly or guide suspicious cases to advanced cost-intensive diagnostic methods like High-Resolution Computed Tomography (HRCT). However, proper diagnostic biomarkers need to comply with several key characteristics [7]. Four important key characteristics are the following: (i) minimal invasiveness to measure the biomarkers in easily accessible body fluids, (ii) robustness against influencing factors, (iii) sufficient sensitivity to detect individuals with cancer, and (iv) high specificity to avoid false positive tests in cancer-free subjects.

Circulating microRNAs (miRNAs) are a well-known class of biomarkers for several diseases, including cancer. The benefit of miRNAs as biomarkers is based on the deregulation in diseased cells and the stability in blood [8]. Thus, it has been suggested that miRNAs are appropriate candidates for liquid biopsies, avoiding the need for invasive procedures to obtain tissue biopsies [9]. For mesothelioma, a multitude of deregulated miRNAs in tissues and cell lines was described [10–18], whereas only three blood-based miRNAs, namely, miR-103a-3p [19], miR-126 [20], and miR-625-3p [21], were identified as potential biomarkers. However, these results indicate the general suitability of miRNAs for the diagnosis of malignant mesothelioma utilizing blood samples.

The aim of the present study was the identification of circulating miRNAs in human plasma as biomarkers for the diagnosis of malignant mesothelioma, the assessment of the identified miRNAs with regard to the key characteristics of proper biomarkers, and the analysis of their performance.

## 2. Materials and Methods

### 2.1. Ethics Statement

All participants of the study provided written informed consent. The study was designed according to the rules guarding patient privacy and with the approval from the ethics committee of the Ruhr-Universität Bochum (reference number: 3217-08).

### 2.2. Study Population

Mesothelioma patients were recruited at the Lungenklinik Heckeshorn, HELIOS Klinikum Emil von Behring, Berlin, Germany, and in participating medical practices of the MoMar study. Cancer-free controls were derived from participants of the MoMar study. The MoMar study is a prospective study comprising annual medical examination and peripheral blood collection of more than 2,000 German workers formerly exposed to asbestos in order to identify and validate molecular biomarkers for the detection of mesothelioma and lung cancer. As the target group for the future application of biomarkers to detect mesothelioma will consist of asbestos-exposed persons, all subjects in the control group were formerly exposed to asbestos.

The study group for the initial discovery phase consisted of 21 male patients with diagnosed mesothelioma, including 14 epithelioid (67%), four biphasic (19%), and three sarcomatoid (14%) mesotheliomas. Six patients underwent partial pleurectomy before blood drawing (median: 31 days; range: 13–148 days). The matched cancer-free control group consisted of 21 men formerly exposed to asbestos. Criteria for matching were age and smoking status.

The study group for the subsequent verification phase consisted of 22 male mesothelioma patients including 14 epithelioid (64%), two biphasic (9%), and two sarcomatoid (9%) mesotheliomas. In four cases, the histological subtype remained unknown. Six patients underwent partial pleurectomy before blood drawing (median: 60 days; range: 24–179 days). The matched cancer-free control group consisted of 44 men formerly exposed to asbestos. Criteria for matching were age and smoking status.

Detailed characteristics of the study groups for discovery and verification are presented in Table 1.

### 2.3. Blood Collection

Blood collection was performed after diagnosis of mesothelioma (median: 19 days; range: 0–731 days). Peripheral blood was collected in 9.0 mL S-Monovette EDTA gel tubes (Sarstedt, Nümbrecht, Germany). Within 30 minutes after blood collection, samples were centrifuged at 2,000 ×g for ten minutes at room temperature. After centrifugation, plasma was separated and frozen immediately until RNA isolation.

### 2.4. Detection of miRNAs

For biomarker discovery, RNA isolation from 1 mL plasma was performed using the mirVana PARIS Kit (Life Technologies, Darmstadt, Germany) according to the manufacturer's instructions, modified by adding 5 *μ*L Carrier RNA MS2 (Roche, Mannheim, Germany). Profiling of 377 miRNAs was performed utilizing commercial TaqMan Low Density Array Human MicroRNA Card A v2.0 (TLDA) according to the manufacturer's instructions (Life Technologies). In brief, 3 *μ*L RNA was used as template for reverse transcription (RT) using the following conditions: 40 cycles of 16°C for 2 minutes, 42°C for 1 minute, and 50°C for 1 second. As template for preamplification, 2.5 *μ*L cDNA was used, using the following conditions: 95°C for 10 minutes, 55°C for 2 minutes, and 72°C for 2 minutes, followed by 12 cycles of 95°C for 15 seconds and 60°C for 4 minutes. Quantitative real-time PCR (qPCR) was carried out using 9 *μ*L of the diluted preamplification product using the following conditions: 94.5°C for 10 minutes, followed by 40 cycles of 97°C for 30 seconds and 59.7°C for 1 minute. All reactions were performed utilizing a 7900 HT Fast Real-Time PCR System (Life Technologies) according to the manufacturer's protocol. Negative controls tested continuously negative.

For biomarker verification, RNA was isolated from 0.5 mL plasma using the mirVana PARIS Kit (Life Technologies) according to the manufacturer's instructions, modified by adding 5 *μ*L Carrier RNA MS2 (Roche). Individual miRNAs were analyzed using commercial TaqMan microRNA assays (Life Technologies; miR-16 (ID 000391), miR-24 (ID 000402), miR-28-3p (ID 002446), miR-126 (ID 002228), miR-132-3p (ID 000457), miR-146b-5p (ID 001097), miR-625-3p (ID 002432), and U6 snRNA (ID 001973)) according to the manufacturer's instructions. In brief, 5 *μ*L RNA was used as template for RT and 5 *μ*L cDNA as template for qPCR. RT is carried out using the following conditions: 16°C for 30 minutes, 42°C for 30 minutes, and 85°C for 5 minutes. PCR is carried out using the following conditions: 95°C for 10 minutes, followed by 40 cycles of 95°C for 15 seconds and 60°C for 1 minute. All reactions were performed in duplicate utilizing a 7300 Real-Time PCR System (Life Technologies). Nontemplate controls were included in all assays and tested continuously negative.

### 2.5. Artificial Hemolysis

The dependence of miRNA levels from hemolysis was analyzed utilizing peripheral blood from three healthy volunteers. Blood samples were centrifuged at 2,000 ×g for ten minutes at room temperature. Plasma and buffy coat layer were separated. Erythrocytes were resuspended in the 2-fold volume of normal saline (0.9%) and mixed. The mixture was centrifuged at 2,000 ×g for five minutes and the supernatant was discarded. Washing steps were repeated twice. For cell lysis, washed erythrocytes were mixed with the 2-fold volume of dH_2_O and incubated for 30 minutes at room temperature. Corresponding plasma samples were spiked with lysed erythrocytes in order to obtain plasma samples with hemolysis grades of 0%, 0.125%, 0.25%, 0.5%, 1%, and 2%.

Amount of free hemoglobin (Hb) in plasma was measured by spectral analysis using a NanoDrop ND-100 spectrophotometer (Thermo Scientific, Braunschweig, Germany). Absorbance was measured at 415 nm (total Hb), 450 nm (bilirubin), and 700 nm (sample turbidity). Hb concentrations were quantified using the formula Hb (ng/mL) = 154.7 × A_415_  − 130.7 × A_450_  − 123.9 × A_700_ [22, 23].

### 2.6. Data Analyses and Statistical Methods

Normalization of TLDA results was performed according to Mestdagh et al. [24] in order to identify proper references suitable for normalization of single assays in the verification phase. As the geometric mean (GM) of combined references might be more reasonable than that of single references [25], the GMs of the candidate references were additionally calculated and analyzed. For the identification of the most stable references, the web-based comprehensive tool RefFinder [26] was used, including the four typically used algorithms, namely, comparative ΔCt method [27], BestKeeper [28], NormFinder [29], and geNorm [25].

Quantitative miRNA expression data were acquired using the ABI SDS software (Life Technologies). For estimation of the cycle threshold (Ct), a fixed threshold of 0.2 was used [30]. Ct values > 35 were considered to be under the detection limit [31]. Thus, for calculation, Ct values > 35 were marked as 35 [32] and miRNA levels were expressed as 2^−ΔCt^ [33].

For evaluation of the influence of the hemolysis grade on miRNA levels, 2^−ΔCt^ values were used and values > 2.0 were marked as significantly upregulated [34].

Box plots with median and interquartile range (IQR) were used to depict the distribution of single biomarkers and their combinations. Whiskers represent minimum and maximum values. Wilcoxon rank-sum tests were applied to compare the distributions of the miRNAs between groups and *p* values < 0.05 were considered as statistically significant. Receiver operating characteristic (ROC) curves were used to quantify classification performance of the biomarkers and the area under curve (AUC) represents the accuracy of the diagnostic test. Biomarker cut-offs were determined utilizing maximum Youden's Index (YI) and fixed false positive rates (FPRs).

Classification performance of individual miRNAs and the combination of two miRNAs were evaluated, investigating three different algorithms to combine the biomarkers. For this purpose,* and*,* or*, and* sequential* combinations of the biomarkers were analyzed. Considering the two biomarkers *X* and *Y*, the sets of marker specific cut-offs are *x*_1_, *x*_2_,…, *x*_*n*_ and *y*_1_, *y*_2_,…, *y*_*n*_, respectively. Applying the* and* algorithm, the two-marker combination was defined as positive if both markers were positive (*X* < *x*_*i*_ and *Y* < *y*_*i*_). Applying the* or* algorithm, the combination was defined as positive if one of the markers was positive (*X* < *x*_*i*_ or *Y* < *y*_*i*_). The* sequential* algorithm is based on sequential screening. In the first step, all tests measured with the marker *X* were considered and defined as positive if *X* < *x*_*i*_. In the second step, negative tests of marker *X* were classified using the marker *Y* and defined as positive if *Y* < *y*_*i*_. AUCs of miRNA combinations were calculated utilizing bootstrap analysis with 500 samples and 95% confidence intervals (CI) were computed directly from distribution of bootstrap estimates. For each bootstrap sample, a ROC curve was generated and AUCs were calculated using the trapezoidal rule.

Potential factors influencing the log-transformed biomarker level were evaluated using a linear regression model. Estimates were given as exp⁡(*β*) with 95% CI.

Statistical analyses were performed using SAS/STAT and SAS/IML software, version 9.4 (SAS Institute Inc., Cary, NC, USA).

## 3. Results

### 3.1. Candidate References

**Figure 1:**
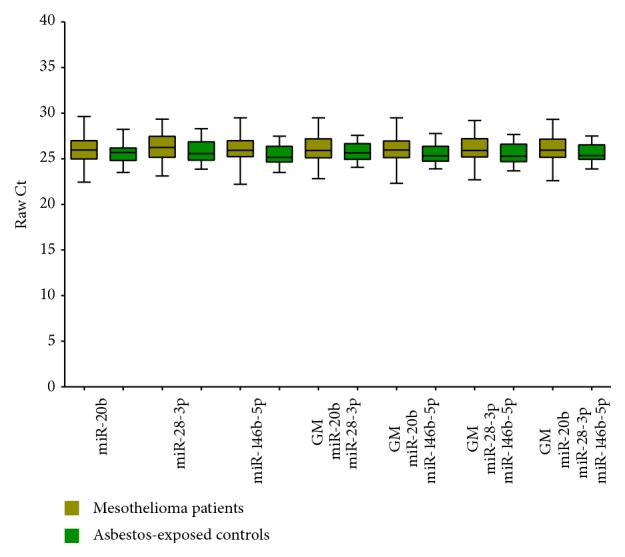
Box plots of raw Ct values of candidate references and geometric mean (GM) of applied combinations.

In order to identify the most stable reference across the studied groups, RefFinder was used to rank the 377 miRNAs measured by TLDAs. Using the four typical algorithms, miR-20b, miR-28-3p, and miR-146b-5p were repeatedly identified throughout the study groups as the top three miRNAs characterized by highest stability. Comparing the raw Ct values of miR-20b, miR-28-3p, and miR-146b-5p and the calculated GMs of all possible combinations, no significant differences between mesothelioma patients and asbestos-exposed controls could be observed (Figure 1). Thus, miR-20b, miR-28-3p, and miR-146b-5p and the four combinations (GM of miR-20b/miR-28-3p, miR-20b/miR-146b-5p, miR-28-3p/miR-146b-5p, and miR-20b/miR-28-3p/miR-146b-5p) were in principal feasible as potential references for subsequent analyses.

### 3.2. Discovery Phase Utilizing TLDAs

Raw Ct values of the remaining 374 miRNAs were normalized using the potential references. ROC analyses were performed to evaluate the performance of the miRNAs to discriminate between mesothelioma cases and asbestos-exposed controls. Biomarkers with more than one false positive test, representing specificity of 95%, were excluded from further analysis. In total, 40 combinations of miRNAs and references showing sensitivities between 86% (representing three false negative tests) and 100% (representing zero false negative tests) were revealed fulfilling the exclusion criteria (Additional File 1 in Supplementary Material available online at https://doi.org/10.1155/2017/9280170). Overall, the 40 combinations consisted of 15 different miRNAs (miR-20b, miR-24, miR-28-3p, miR-132-3p, miR-140-3p, miR-146b-5p, miR-155, miR-191, miR-193a-5p, miR-328, miR-331, miR-381, miR-532, miR-628-5p, and miR-660) that were used for further analysis.

### 3.3. Influence of Hemolysis on miRNA Levels in Plasma

**Figure 2:**
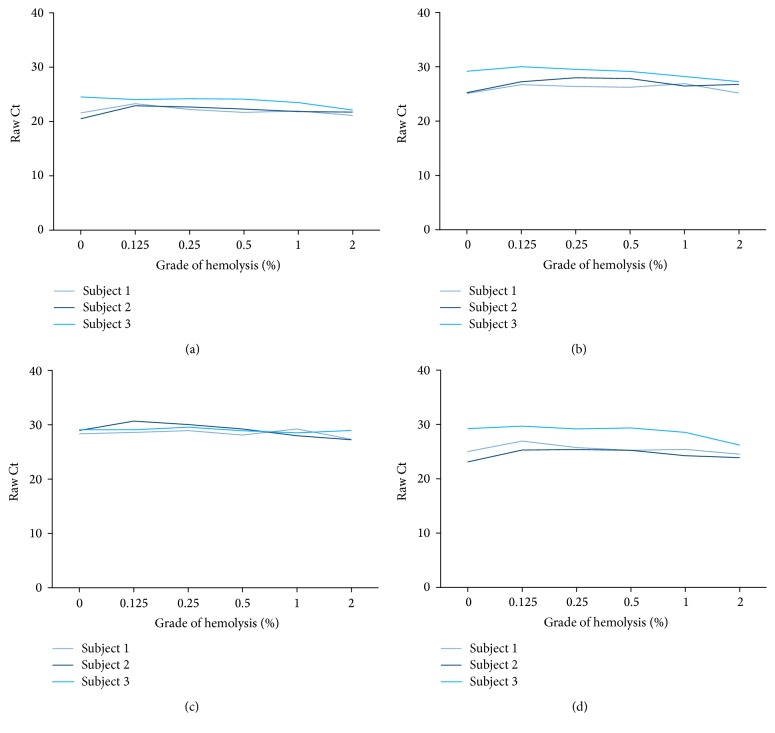
Influence of hemolysis grade on raw miRNA levels (a) miR-24, (b) miR-28-3p, (c) miR-132-3p, and (d) miR-146b-5p in plasma spiked with lysed erythrocytes.

As miRNA levels in plasma might be altered by miRNA release during lysis of erythrocytes, the influence of hemolysis on the identified miRNAs in plasma was evaluated. Plasma samples with ascending amounts of spiked-in lysed erythrocytes (0.125%, 0.25%, 0.5%, 1%, and 2%) exhibit rising free hemoglobin levels in comparison to the nonhemolyzed (0%) samples (Additional File 2). The 15 miRNAs were measured in plasma samples with different grades of hemolysis. No significant fold changes > 2.0 could be observed for miR-24, miR-28-3p, miR-132-3p, and miR-146b-5p at low hemolysis grades < 0.5% (Figure 2 and Additional File 3). In contrast, most of the analyzed miRNAs were distinctly influenced by hemolysis, showing fold changes > 2 already at low hemolysis grades (Additional File 3). Thus, these miRNAs were excluded from further analyses. Notably, miR-381 seemed to be not affected by hemolysis, whereas the corresponding reference miR-20b was highly affected even at low hemolysis grades. In addition, the detectability of miR-193-5p in plasma using single assays was not reliable (data not shown). Thus, these two miRNAs were also excluded from further analysis.

### 3.4. Verification of TLDA Results Using Individual Assays

Based on the discovery results and the preanalytical analysis, four combinations of miRNAs and references were analyzed in the subsequent verification phase (Table 2). All combinations showed a statistically significant downregulation (*p* < 0.001) in the initiating TLDA results between mesothelioma patients and asbestos-exposed controls (Additional File 4).

**Figure 3:**
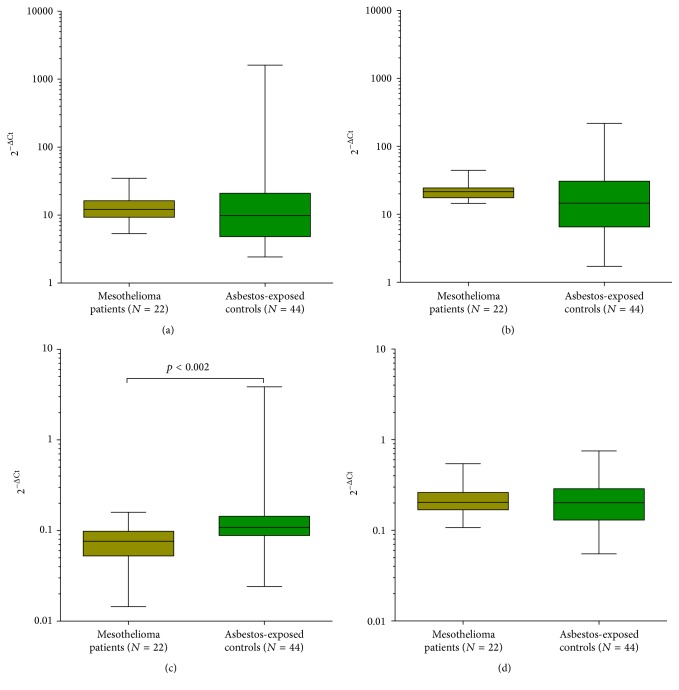
Box plots of the analyzed miRNA combinations in the verification study group (a) miR-24, miR-146b-5p, (b) miR-24, GM (miR-146b-5p and miR-28-3p), (c) miR-132-3p, miR-146b-5p, and (d) miR-132-3p, miR-28-3p (GM: geometric mean). Wilcoxon rank-sum tests were performed to examine group differences.

Analyzing the four combinations using individual assays, a statistically significant difference (*p* = 0.002) between the mesothelioma patients and the control group was confirmed only for miR-132-3p using miR-146b-5p as reference (Figure 3(c)). Median level of miR-132-3p was 0.08 (IQR: 0.05–0.10) in mesothelioma patients and 0.11 (IQR: 0.09–0.14) in cancer-free controls. In contrast, the other three combinations showed no significant differences between the two studied groups (Figures 3(a), 3(b), and 3(d)).

As only two sarcomatoid and two biphasic mesothelioma were in the study group, no analysis regarding the histological subtype was performed.

**Figure 4:**
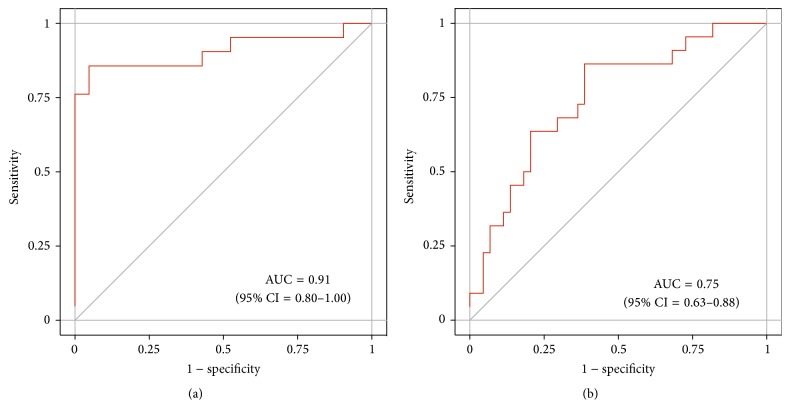
Receiver operating characteristic (ROC) curves of miR-132-3p in (a) the discovery group and (b) the verification group.

In order to evaluate the performance of miR-132-3p, ROC analyses were conducted, revealing an AUC of 0.91 (95% CI = 0.80–1.00) in the discovery group (Figure 4(a)) and an AUC of 0.75 (95% CI = 0.63–0.88) in the verification group (Figure 4(b)). For the discrimination of mesothelioma cases from cancer-free subjects, sensitivities and specificities of miR-132-3p were calculated (Table 3). Utilizing maximum YI revealed 86% sensitivity and 61% specificity. Using fixed FPRs of 0%, 5%, and 10% revealed sensitivities of 5%, 23%, and 36% and specificities of 100%, 95%, and 90%, respectively.

Surgical treatment of mesothelioma, that is, pleurectomy, might influence biomarkers levels. Thus, six mesothelioma cases that underwent at least partial pleurectomy were excluded. ROC analysis revealed a similar AUC for miR-132-3p (0.76; 95% CI = 0.62–0.89) as seen in the entire verification group.

### 3.5. Potential Influencing Factors

The impact of two potential influencing factors on the levels of miR-132-3p was analyzed in subjects without malignant disease. Neither age nor the smoking status influenced the miR-132-3p levels in human plasma (Table 4).

### 3.6. Combination of Candidate Biomarkers

The two miRNAs miR-126 (U6 snRNA as reference) and miR-625-3p (miR-16 as reference) were previously described as circulating biomarkers for mesothelioma [20, 21]. In order to improve the biomarker performance of miR-132-3p (miR-146b-5p as reference), miR-126 and miR-625-3p and their corresponding references U6 snRNA and miR-16, respectively, were additionally measured in the verification set to evaluate the discrimination potential of individual biomarkers and biomarker combinations. Statistically significant downregulation of miR-126 (*p* < 0.001) between mesothelioma cases and asbestos-exposed controls could be observed in contrast to miR-625-3p (Additional File 5). For miR-126 an AUC of 0.76 (95% CI: 0.64–0.87) and for miR-625-3p an AUC of 0.58 (95% CI: 0.44–0.72) were calculated (Additional File 5). Thus, for combination analysis, miR-126 was selected, whereas miR-625-3p was excluded from further analysis.

**Figure 5:**
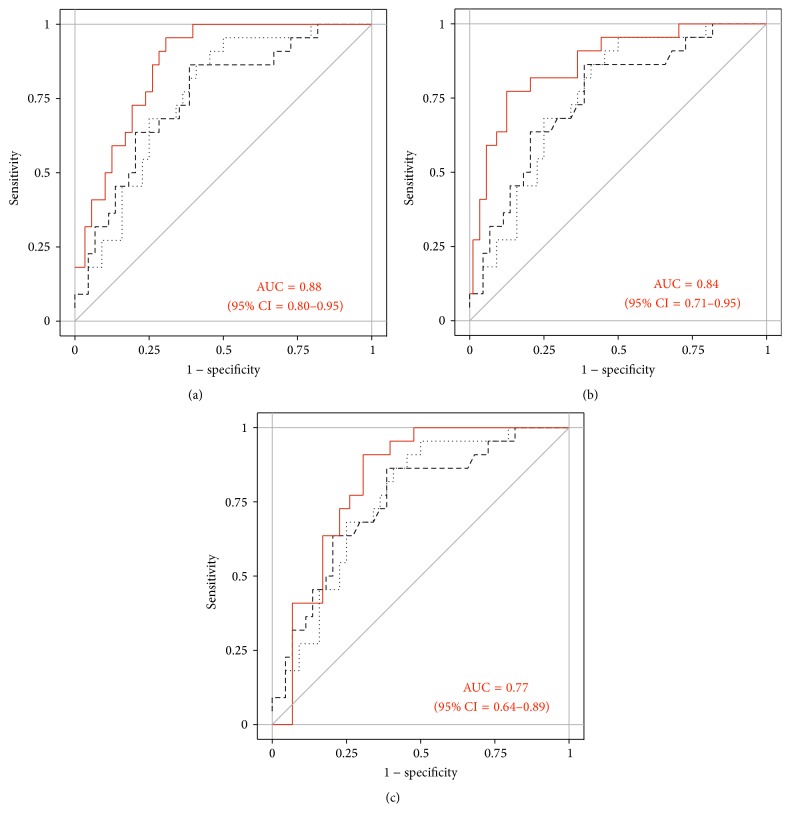
Receiver operating characteristic (ROC) curves of miR-132-3p (dashed line), miR-126 (dotted line), and (a) the* sequential* algorithm, (b) the* and* algorithm, and (c) the* or* algorithm of miR-132-3p/miR-126 (solid line) in the verification group.

In order to ascertain the best combination of miR-132-3p and miR-126, three different approaches were tested, namely,* sequential* algorithm,* and* algorithm, and* or* algorithm. Overall, the miR-132-3p/miR-126 combination improved the diagnostic performance. The AUCs of miR-132-3p/miR-126 were 0.88 (95% CI = 0.80–0.95) for the* sequential *algorithm, 0.84 (95% CI = 0.71–0.95) for* and *algorithm, and 0.77 (95% CI = 0.64–0.89) for* or* algorithm (Figure 5). For the discrimination of mesothelioma cases from asbestos-exposed subjects, sensitivities and specificities using the* sequential* algorithm and* and* algorithm of miR-132-3p/miR-126 were calculated (Tables 5 and 6). Performing bootstrap analyses of 500 samples indicated a good precision of the assessments (Additional File 6). The* and* algorithm showed higher sensitivities at applicable high specificities of 89% and 95%. Thus, it was indicated that the* and* algorithm of miR-132-3p/miR-126 might be the best strategy to improve the diagnostic performance. Utilizing maximum YI revealed 77% sensitivity and 86% specificity. Using fixed FPRs of 0%, 5%, and 11%, sensitivities of 9%, 41%, and 64% and specificities of 100%, 95%, and 89% were calculated, respectively.

Excluding six mesothelioma cases, which underwent at least partial pleurectomy, from ROC analysis showed no differences (data not shown).

## 4. Discussion

Analysis of liquid biopsies is a promising approach in translational cancer research and can be performed in almost all body fluids [35]. Generally, there is no need for an invasive procedure to obtain body fluids like blood. Thus, it might be the preferable choice for diagnostic procedures to detect cancer at early stages. Screening for the early detection of cancer might be especially meaningful in at-risk populations. As the main cause for mesothelioma is the former exposure to asbestos, the target population for screening of mesothelioma is people with a former exposure to asbestos. The incidence of mesothelioma is too low to justify screening in the general population.

For the diagnosis of cancer, the use of miRNAs as biomarkers provided new opportunities [36]. For quantitative expression analysis of miRNAs, qPCR is considered to be the gold standard [37]. In this context, the use of appropriate references for normalization is an important issue [38]. The three identified candidate references miR-20b, miR-28-3p, and miR-146b-5p showed no statistically significant differences between asbestos-exposed controls and mesothelioma patients. However, the raw Ct values showed a small but consistent increase in the mesothelioma group in comparison to the control group. This might be due to the general characteristic of miRNAs to reflect tiny pathological variations [39]. Additionally, the variations of the raw Ct values are rather large in the analyzed groups. This might be due to interindividual differences of the participants within the groups. Circulating miRNAs are strongly influenced by several personal characteristics, for example, age, BMI, and gender [40]. Thus, prior to the use of miRNAs in screening routine, the miRNAs should be analyzed in large study groups with detailed information about personal characteristics to assess potential influencing factors leading to interindividual differences. In addition, the feasibility of endogenous references is also influenced by preanalytical variations, for example, differences in RNA extraction efficiency and possible PCR inhibitors [41]. Thus, the addition of an exogenous miRNA like cel-miR-39 might be very meaningful in order to obtain more reliable results.

In this study, human plasma was used to analyze the expression of 377 miRNAs in samples of cancer patients and asbestos-exposed controls in order to identify circulating miRNAs as candidate biomarkers for the detection of malignant mesothelioma. In patients with diagnosed mesothelioma, miR-132-3p was significantly downregulated in comparison to cancer-free subjects. To the best of our knowledge, this is the first time to show miR-132-3p as a biomarker for malignant mesothelioma. Comparable results were obtained for ovarian cancer, showing significant downregulation of circulating miR-132-3p in serum of cancer patients [42]. It is common that the same biomarkers can detect different tumor entities and this might be also true for mesothelioma and ovarian cancer. Accordingly, it has been shown that even mesothelin, the most prominent biomarker for mesothelioma, might be a suitable biomarker to detect ovarian cancer [43]. Notably, in lung cancer, a cancer also associated with asbestos exposure; miR-132-3p was shown to be downregulated as well [44]. Thus, it might be interesting in future studies to analyze miR-132-3p in lung cancers, especially with regard to the differential diagnosis of mesothelioma and adenocarcinomas of the lung.

A trend towards global downregulation of miRNAs is indicated in several cancers [45, 46] as well as in mesothelioma [11, 20]. For several downregulated miRNAs, a tumor suppressor function has been suggested, regulating the overexpression of certain proteins in the pathogenesis of malignant mesothelioma [13, 15, 47]. Recently, Lei et al. identified YAP (Yes-associated protein) as a target of miR-132, showing that miR-132 alters the expression of YAP at the mRNA and protein level in hepatocellular carcinomas [48]. YAP is a key oncoprotein of the Hippo pathway, a downstream cascade of Merlin, regulating cellular properties linked to carcinogenesis [49]. Enhanced YAP expression might be a common event in certain cancers [50], and particularly in mesothelioma YAP is constitutively active in more than 70% of the primary tumors [51], promoting cell proliferation [52]. Thus, it might be reasonable that miR-132-3p also regulates the expression of YAP in malignant mesothelioma. The elucidation of a possible regulatory function of miR-132-3p could offer additional insights in the pathogenesis of mesothelioma and might lead to new candidate targets for therapy. Unfortunately, a possible effect of miR-132-3p on YAP expression could not be investigated because YAP mRNA was not detectable in the plasma samples in this study. Therefore, appropriate studies (preferably in tumor tissues) are needed to follow up on the possible interaction between miR-132-3p and YAP in mesothelioma.

Biomarker candidates need to fulfill several key characteristics [7], namely, minimal invasiveness, robustness, sensitivity, and specificity. It is well known that circulating miRNAs are easily detectable in serum and plasma [53] and this is also shown in this study. Thus, miR-132-3p fulfills the first key feature to be minimally invasive. Regarding the second feature robustness, the most problematic influencing factor for circulating miRNAs, which are generally considered to be stable, might be hemolysis. Recently, Kirschner et al. showed that the grade of hemolysis significantly influences the levels of miRNAs in plasma and serum [54]. Additionally, it has been suggested that a multitude of miRNAs published as candidate biomarkers are rather artifacts of hemolysis than being influenced by the disease they were supposed to detect [55]. Hemolysis frequently occurs in clinical routine and it is indicated that 3.3% of all samples are hemolytic [56]. Generally, high grades of hemolysis show distinct red colored samples and might not be appropriate for diagnostic analyses. However, lower grades of hemolysis do not lead to obviously colored plasma but might still influence the miRNA levels in the samples. Thus, it is essential to analyze the dependence of miRNAs from hemolysis prior to the assessment of the biomarker performance, particularly at low grades of hemolysis. In order to exclude circulating miRNAs that are affected by hemolysis, 15 miRNAs identified in the discovery phase were analyzed with respect to their dependence on hemolysis. Most miRNAs were clearly affected by hemolysis, even at low hemolytic levels, and had to be excluded from further analysis. Accordingly, they are not appropriate for the use as reliable biomarkers because they do not fulfill the second key characteristic to be robust. In contrast, the results indicate that miR-132-3p and the corresponding reference miR-146b-5p are not affected by low-grade hemolysis and thus are sufficiently robust.

Regarding the usual influencing factors age and smoking status, miR-132-3p seems to be relatively independent, further indicating the robustness of the candidate biomarker. However, the results are based on small numbers. Thus, a detailed analysis in a large study group of healthy subjects without malignant diseases should be performed to evaluate the impact of influencing factors [7].

Surgical treatment of mesothelioma might lead to an alteration of biomarker levels as shown for mesothelin [57]. Regarding partial pleurectomy and therefore at least a substantial reduction in tumor burden, no influence of the miR-132-3p performance was observable between the study groups, also implicating that the biomarkers might be robust. Comparable results were obtained for miR-126 showing that the marker level did not change after tumor reduction [57].

Regarding the key features sensitivity and specificity, it is not likely that a single biomarker is sufficient for the diagnosis of a disease. Rather, it is expected that only the combination of individual biomarkers in a marker panel can achieve sufficient sensitivity at fixed high specificity for diagnostic purposes. Thus, two already described circulating miRNAs, namely, miR-126 [20] and miR-625-3p [21], were additionally analyzed in this study group. Notably, miR-126 and miR-625-3p are not affected by hemolysis [55]. Whereas miR-625-3p failed to show a significant difference in this study, miR-126 discriminated between cancer cases and controls. For miR-126 alone, sensitivity of 86% and specificity of 59% (maximum YI) were calculated in this study, whereas Santarelli et al. calculated 73% sensitivity and 74% specificity for the discrimination of mesothelioma patients from asbestos-exposed controls [20].

Accordingly, the combination of miR-132-3p and miR-126 was assessed, showing an improved diagnostic performance. Despite the slightly higher AUC for the* sequential* algorithm, it was indicated that the* and* algorithm of miR-132-3p/miR-126 might be the best strategy to improve the diagnostic performance due to higher sensitivities at applicable high specificities of 89% and 95% and the easy implementation of the* and* algorithm in clinical routine (the two-marker combination is defined as positive if both markers are positive). Assigning fixed specificities, sensitivities were enhanced by the factor 1.8 for the* and* combination of miR-132-3p/miR-126 in comparison to miR-132-3p alone. These results indicate that the combination of biomarkers within a panel improves the diagnostic performance. However, using miR-132-3p and miR-126 within a panel, two different references for normalization are needed, implicating a less robust method. Thus, it might be preferable to compile a panel of several miRNAs as biomarkers with a single reference for normalization. Additionally, biomarkers of different molecular classes like proteins (e.g., mesothelin [58] and calretinin [59]) or methylated DNA (e.g.,* ZIC1* [60] and* RASSF1A* [61]) could be tested in combination with the miRNAs and might be added to a future marker panel in order to further improve the diagnostic performance.

Malignant mesothelioma is an aggressive cancer and symptoms of mesothelioma commonly occur at late stages of the disease. Depending on treatment, the median survival after diagnosis is between nine and 13 months [62]. Thus, there is a need to detect the cancer at early stages to improve therapeutic outcome, ideally resulting in a decreased mortality. For the use of proper biomarkers in cancer screening procedures, high specificity of at least 95% is required to minimize false positive tests, because they might lead to psychological distress and unnecessary diagnostic interventions or even therapies. However, for subjects formerly exposed to asbestos, there is an increased risk to develop asbestos-associated cancers like lung cancer or mesothelioma [63, 64]. Thus, slightly lower specificity might be tolerable for the use in at-risk collectives.

However, as the study group is relatively small, the results should be verified in larger study groups, including a sufficient number of epithelioid as well as biphasic and sarcomatoid mesotheliomas. Additionally, to assess the feasibility of miR-132-3p and miR-126 to detect the cancer at early stages, studies with a prospective design are urgently needed [7]. Regarding malignant mesothelioma, the prospective study MoMar started in 2008 comprising more than 2,000 German workers formerly exposed to asbestos. The sample collection will end in 2017 and afterwards the potential of candidate biomarkers for the early detection of the cancers will be evaluated.

## 5. Conclusions

We identified miR-132-3p as a new candidate biomarker for malignant mesothelioma, showing significantly different expression levels between mesothelioma patients and cancer-free controls formerly exposed to asbestos. Thus, the circulating miR-132-3p might be useful for diagnosis of mesothelioma utilizing human plasma samples. Plasma levels of miR-132-3p are not altered by hemolysis, the most problematic influencing factor for circulating miRNAs. Combination of miR-132-3p with miR-126 improved diagnostic performance, resulting in enhanced sensitivity and specificity. To evaluate the use of the miR-132-3p/miR-126 combination for the detection of malignant mesothelioma at early stages, studies with a prospective design are urgently needed.

## Supplementary Material

Additional File 1: Performance of potential biomarkers in the discovery phase. Additional File 2: Hemoglobin levels in artificial hemolyzed plasma samples. Additional File 3: miRNA foldchanges in artificial hemolyzed plasma samples. Additional File 4: Box plots of candidate biomarkers. Additional File 5: Box plot and ROC curve of additional analyzed miRNAs. Additional File 6: Performance of biomarkers utilizing bootstrap analysis. Additional File 7: Raw Data.

## Figures and Tables

**Table 1:** Characteristics of the study groups.

		Discovery		Verification	
		Mesothelioma patients (*N*)	Asbestos-exposed controls (*N*)	Mesothelioma patients (*N*)	Asbestos-exposed controls (*N*)
Gender	Male	21	21	22	44

Age (years)	Median (range)	72 (35–85)	72 (43–82)	72 (39–85)	72 (49–85)

Smoking status^*∗*^	Ever	12	12	9	20
	Never	9	9	11	22

Histological subtype	Epithelioid	14		14	
	Biphasic	4		2	
	Sarcomatoid	3		2	
	Not specified	0		4	

^*∗*^Smoking status was not available for four participants.

**Table 2:** Analyzed combinations in the verification phase (GM: geometric mean).

miRNA	Reference
miR-24	miR-146b-5p
miR-24	GM (miR-146b-5p/miR-28-3p)
miR-132-3p	miR-146b-5p
miR-132-3p	miR-28-3p

**Table 3:** Sensitivity and specificity of miR-132-3p and number of true positive, true negative, false positive, and false negative tests, calculated for maximum Youden's Index (YI) and fixed false positive rates (FPRs) of 0%, 5%, and 10%.

FPR (%)	Cut-off	Sensitivity (%)	Specificity (%)	True positive (*N*)	True negative (*N*)	False positive (*N*)	False negative (*N*)
	2^−(miR-132-3p - miR-146b)^						
Maximum YI	0.100	86	61	19	27	17	3
0	0.014	5	100	1	44	0	21
5	0.047	23	95	5	42	2	17
10	0.063	36	90	8	39	5	14

**Table 4:** Estimates of the influence of age and smoking status on miR-132-3p.

Factor	*N*	exp (*β*)	95% CI
Intercept		0.11	0.01–1.40
Age (per one year)	42	1.00	0.97–1.04
Ever smoking	20	0.94	0.54–1.67
Adjusted *R*^2^		−0.05	

**Table 5:** Sensitivity and specificity of the *sequential* algorithm for miR-132-3p/miR-126 and number of true positive, true negative, false positive, and false negative tests, calculated for maximum Youden's Index (YI) and fixed false positive rates (FPRs) of 0%, 5%, and 11%.

FPR (%)	Cut-off		Sensitivity (%)	Specificity (%)	True positive (*N*)	True negative (*N*)	False positive (*N*)	False negative (*N*)
	2^−(miR-132-3p - miR-146b)^	2^−(miR-126 - U6-snR)^						
Maximum YI	0.080	3.51	95	68	21	30	14	1
0	0.020	0.10	18	100	4	44	0	18
5	0.050	0.10	32	95	7	42	2	15
11	0.060	0.62	50	89	11	39	5	11

**Table 6:** Sensitivity and specificity of the *and* algorithm for miR-132-3p/miR-126 and number of true positive, true negative, false positive, and false negative tests, calculated for maximum Youden's Index (YI) and fixed false positive rates (FPRs) of 0%, 5%, and 11%.

FPR (%)	Cut-off		Sensitivity (%)	Specificity (%)	True positive (*N*)	True negative (*N*)	False positive (*N*)	False negative (*N*)
	2^−(miR-132-3p - miR-146b)^	2^−(miR-126 - U6-snR)^						
Maximum YI	0.102	34.27	77	86	17	38	6	5
0	0.132	0.62	9	100	2	44	0	20
5	0.085	19.51	41	95	9	42	2	13
11	0.095	62.60	64	89	14	39	5	8

## Data Access

The raw data supporting the conclusions of this article are included as Additional File 7.
