# Supplementary material for: Circulating miR-132-3p as a Candidate Diagnostic Biomarker for Malignant Mesothelioma
Source: Dis Markers. 2017 Feb 21;2017:9280170. doi: 10.1155/2017/9280170 (PMC5339541; doi:10.1155/2017/9280170)
Supplement: Supplementary file 1 — Additional File 1: Performance of potential biomarkers in the discovery phase. Additional File 2: Hemoglobin levels in artificial hemolyzed plasma samples. Additional File 3: miRNA foldchanges in artificial hemolyzed plasma samples. Additional File 4: Box plots of candidate biomarkers. Additional File 5: Box plot and ROC curve of additional analyzed miRNAs. Additional File 6: Performance of biomarkers utilizing bootstrap analysis. Additional File 7: Raw Data. [file 9280170.f1.pdf]

Additional File 1. Sensitivity and specificity of candidate biomarkers in the discovery phase (GM: geometric mean).

miRNA	Reference	Sensitivity (%)	Specificity (%)	True positive (N)	True negative (N)	False positive (N)	False negative (N)
miR-331	miR-146b-5p	100	95	21	20	1	0
miR-24	miR-146b-5p	90	95	19	20	1	2
miR-532	miR-146b-5p	90	95	19	20	1	2
miR-132	miR-146b-5p	86	95	18	20	1	3
miR-140-3p	miR-146b-5p	86	95	18	20	1	3
miR-191	miR-146b-5p	86	95	18	20	1	3
miR-628-5p	miR-146b-5p	86	95	18	20	1	3
miR-532	miR-20b	95	95	20	20	1	1
miR-660	miR-20b	90	95	19	20	1	2
miR-193a-5p	miR-20b	86	95	18	20	1	3
miR-381	miR-20b	86	95	18	20	1	3
miR-628-5p	miR-20b	86	95	18	20	1	3
miR-191	miR-28-3p	90	95	19	20	1	2
miR-132	miR-28-3p	86	95	18	20	1	3
miR-155	miR-28-3p	86	95	18	20	1	3
miR-331	GM (miR-20b/miR-146b-5p)	100	95	21	20	1	0
miR-140-3p	GM (miR-20b/miR-146b-5p)	95	95	20	20	1	1
miR-532	GM (miR-20b/miR-146b-5p)	95	95	20	20	1	1
miR-24	GM (miR-20b/miR-146b-5p)	90	95	19	20	1	2
miR-328	GM (miR-20b/miR-146b-5p)	90	95	19	20	1	2
miR-628-5p	GM (miR-20b/miR-146b-5p)	86	95	18	20	1	3
miR-660	GM (miR-20b/miR-146b-5p)	86	95	18	20	1	3
miR-140-3p	GM (miR-28-3p/miR-146b-5p)	95	95	20	20	1	1
miR-24	GM (miR-28-3p/miR-146b-5p)	95	95	20	20	1	1
miR-191	GM (miR-28-3p/miR-146b-5p)	90	95	19	20	1	2
miR-331	GM (miR-28-3p/miR-146b-5p)	90	95	19	20	1	2
miR-532	GM (miR-28-3p/miR-146b-5p)	90	95	19	20	1	2
miR-140-3p	GM (miR-20b/miR-28-3p)	95	95	20	20	1	1
miR-532	GM (miR-20b/miR-28-3p)	95	95	20	20	1	1
miR-24	GM (miR-20b/miR-28-3p)	90	95	19	20	1	2
miR-331	GM (miR-20b/miR-28-3p)	90	95	19	20	1	2
miR-191	GM (miR-20b/miR-28-3p)	86	95	18	20	1	3
miR-628-5p	GM (miR-20b/miR-28-3p)	86	95	18	20	1	3
miR-660	GM (miR-20b/miR-28-3p)	86	95	18	20	1	3
miR-140-3p	GM (miR-20b/miR-28-3p/miR-146b-5p)	95	95	20	20	1	1
miR-331	GM (miR-20b/miR-28-3p/miR-146b-5p)	95	95	20	20	1	1
miR-532	GM (miR-20b/miR-28-3p/miR-146b-5p)	95	95	20	20	1	1
miR-24	GM (miR-20b/miR-28-3p/miR-146b-5p)	90	95	19	20	1	2
miR-191	GM (miR-20b/miR-28-3p/miR-146b-5p)	86	95	18	20	1	3
miR-628-5p	GM (miR-20b/miR-28-3p/miR-146b-5p)	86	95	18	20	1	3

Additional File 2. Hemoglobin levels (g/l) in plasma samples spiked-in with lysed erythrocytes of three voluntary subjects.

**Subject**	**Grade of hemolysis (%)**					
	**0**	**0.125**	**0.25**	**0.5**	**1**	**2**
1	0.15	0.24	0.32	0.63	1.11	1.91
2	0.18	0.47	n.d.	0.82	1.28	2.23
3	0.09	0.21	0.39	0.62	1.07	1.89

n.d.: not determined

Additional File 3. Foldchanges of miRNAs in plasma samples spiked-in with lysed erythrocytes. Significant increased fold changes (> 2) are marked red.

miRNA	Subject	Grade of hemolysis (%)				
		0.125	0.25	0.5	1	2
miR-140-3p	1	1.1	2.3	2.2	3.8	9.5
	2	2.2	1.3	2.3	9.1	25.6
	3	1.8	3.9	4.5	22.5	22.8
miR-381	1	0.8	1.0	0.6	0.6	1.0
	2	0.3	0.2	0.3	0.3	0.2
	3	0.4	0.7	1.1	1.1	1.2
miR-155	1	0.5	0.6	0.6	0.3	1.4
	2	0.2	0.2	0.2	0.3	0.3
	3	1.0	1.5	1.8	3.3	7.9
miR-660	1	1.0	1.9	2.7	6.1	14.6
	2	3.7	2.4	4.3	15.8	23.3
	3	1.8	3.6	7.3	13.2	24.8
miR-24	1	0.3	0.6	0.9	0.8	1.4
	2	0.2	0.2	0.3	0.4	0.4
	3	1.4	1.2	1.3	2.0	5.4
miR-191	1	0.7	1.0	1.6	2.3	4.1
	2	0.3	0.4	0.6	1.3	2.1
	3	1.7	2.0	2.5	5.1	17.7
miR-331	1	0.5	0.7	1.0	2.1	5.6
	2	0.7	0.4	0.9	3.1	4.3
	3	0.6	1.3	2.3	5.6	27.5
miR-20b	1	0.5	1.6	4.0	7.5	20.1
	2	2.1	1.8	4.9	13.2	27.3
	3	3.0	7.4	10.4	20.5	148.1
miR-28-3p	1	0.3	0.4	0.5	0.3	0.9
	2	0.2	0.1	0.2	0.4	0.3
	3	0.6	0.8	1.0	1.9	3.8
miR-132-3p	1	0.8	0.7	1.2	0.5	2.0
	2	0.3	0.5	0.8	2.0	3.3
	3	1.0	0.7	1.1	1.5	1.1
miR-146b	1	0.3	0.6	0.9	0.8	1.4
	2	0.2	0.2	0.2	0.5	0.6
	3	0.7	1.0	0.9	1.6	8.1
miR-532	1	0.5	1.2	2.4	4.4	13.2
	2	1.7	1.3	2.5	6.8	13.6
	3	1.7	2.5	4.7	16.4	57.3
miR-328	1	0.2	0.4	1.0	0.6	2.2
	2	0.2	0.2	0.3	0.6	0.7
	3	1.4	2.1	3.2	5.0	11.4
miR-193a-5p	1	0.8	0.5	1.0	1.5	2.2
	2	1.0	0.9	1.0	1.2	1.6
	3	1.1	0.7	0.7	2.1	1.6
miR-628-5p	1	1.0	1.0	3.6	4.5	13.2
	2	0.2	0.2	0.4	1.6	1.1
	3	1.0	1.0	1.2	1.0	5.9

Additional File 4. Box plot of the four approaches in the discovery group using TLDAs. (A) miR-24 – miR-146b-5p, (B) miR-24 – GM (miR-146b-5p/miR-28-3p), (C) miR-132-3p – miR-146b-5p, and (D) miR-132-3p – miR-28-3p. Wilcoxon rank sum tests were performed to examine group differences.

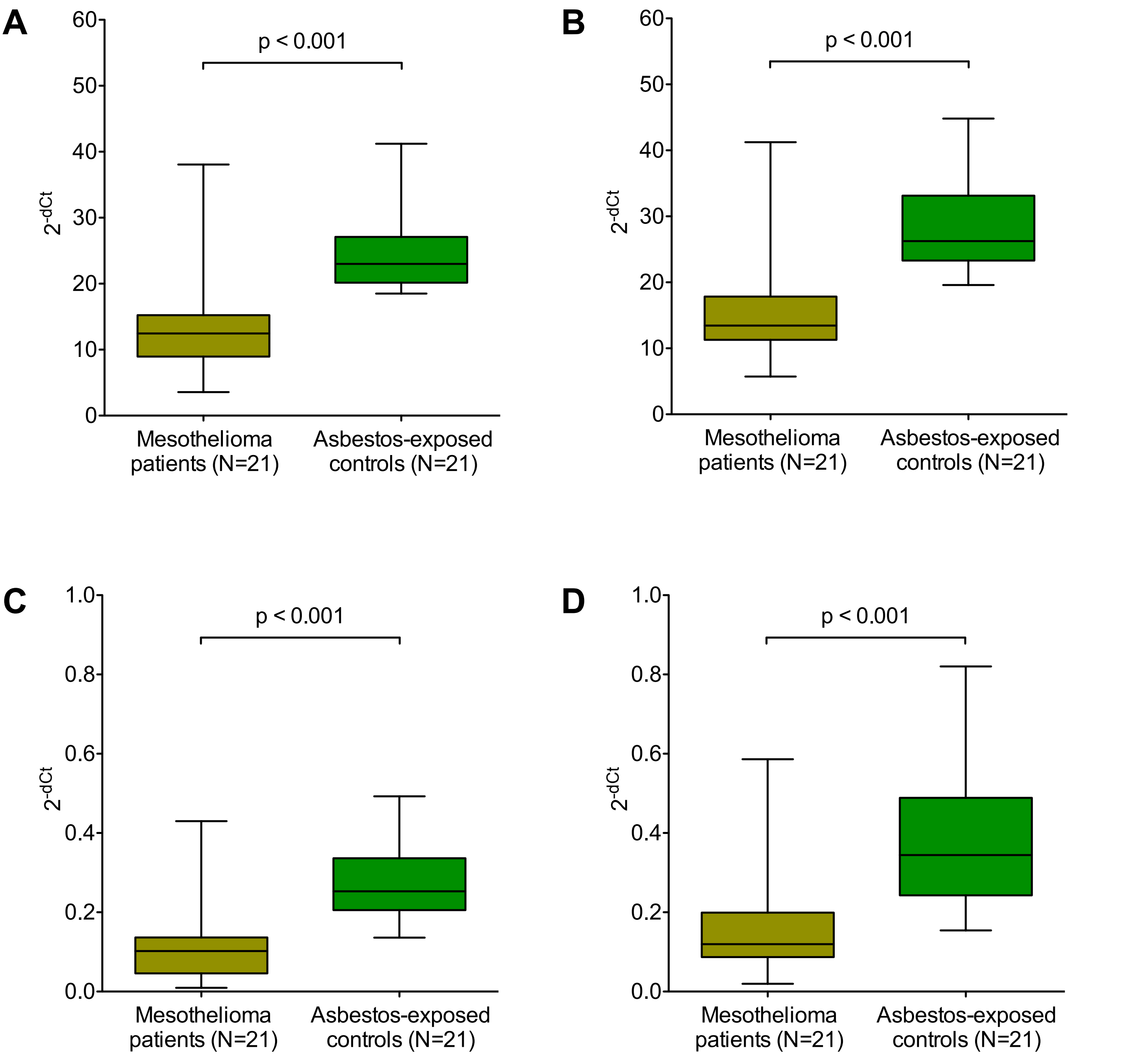

Additional File 5. Box plots and receiver operating characteristic (ROC) curves for miR-126 (A, B) and miR-625-3p (C, D) in the verification study group. Wilcoxon rank sum tests were performed to examine group differences.

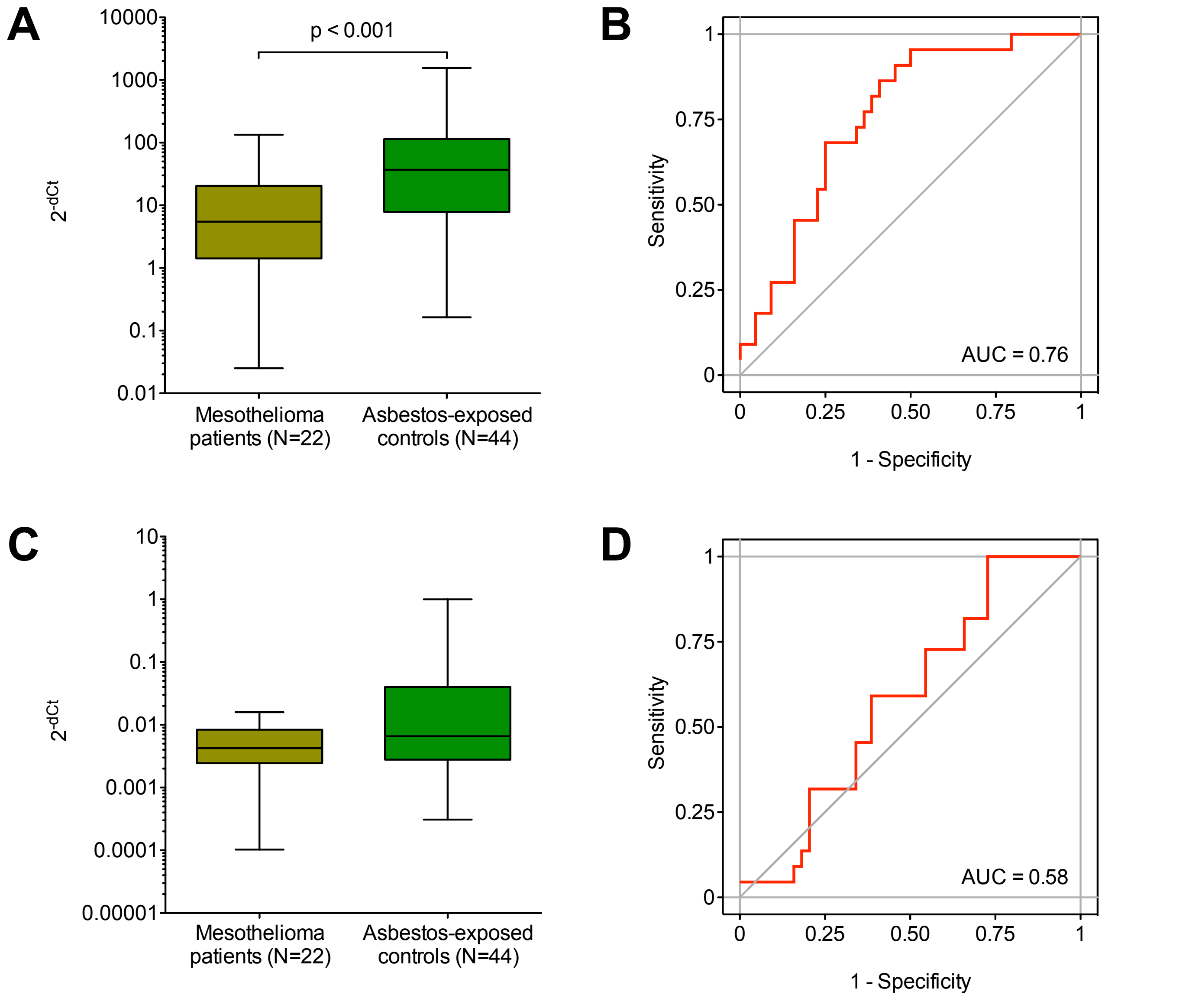

Additional File 6. Sensitivity and specificity of the *and* and *sequential* algorithms for miR-132-3p/miR-126, calculated for maximum Youden's Index (YI) and fixed false positive rates (FPR) of 0%, 5%, and 11%, utilizing bootstrap analysis with 500 samples.

**Algorithm**	**FPR (%)**	**Sensitivity (95% CI)**	**Specificity (95% CI)**
and	Maximum YI	80 (59 - 95)	85 (61 - 98)
	0	22 (5 - 64)	100
	5	43 (9 - 82)	95
	11	58 (23 - 91)	89
sequential	Maximum YI	95 (82 - 100)	73 (57 - 89)
	0	24 (9 - 50)	100
	5	37 (9 - 68)	95
	11	57 (23 - 86)	89

Additional File 7 A. Raw data of biomarker discovery utilizing commercial TaqMan Low Density Array Human MicroRNA Card A v2.0 (TLDA).

Age	Gender	Smoking status	Group	Subtype	ath-miR159a	hsa-let-7a	hsa-let-7b	hsa-let-7c	hsa-let-7d	hsa-let-7e	hsa-let-7f
54	Male	Ever	Mesothelioma	Biphasic	40,00	40,00	29,00	40,00	30,31	26,83	40,00
72	Male	Ever	Mesothelioma	Biphasic	40,00	40,00	31,02	40,00	34,05	29,39	40,00
68	Male	Never	Mesothelioma	Epithelioid	40,00	40,00	27,32	31,93	29,20	26,00	40,00
34	Male	Ever	Mesothelioma	Epithelioid	40,00	28,22	24,71	31,51	26,30	23,44	40,00
70	Male	Never	Mesothelioma	Biphasic	40,00	29,71	25,80	31,13	27,58	24,59	40,00
59	Male	Never	Mesothelioma	Epithelioid	40,00	40,00	26,74	40,00	28,03	25,17	40,00
74	Male	Never	Mesothelioma	Epithelioid	40,00	32,97	28,91	34,83	31,09	27,95	40,00
73	Male	Never	Mesothelioma	Epithelioid	40,00	29,23	24,94	31,29	27,08	25,41	40,00
72	Male	Never	Mesothelioma	Sarcomatoid	40,00	25,01	23,07	30,22	24,28	22,02	30,28
66	Male	Ever	Mesothelioma	Epithelioid	40,00	27,24	22,98	29,20	25,38	23,00	40,00
73	Male	Ever	Mesothelioma	Biphasic	40,00	31,15	25,70	33,08	28,84	26,24	40,00
53	Male	Never	Mesothelioma	Epithelioid	40,00	40,00	27,39	40,00	28,52	26,08	40,00
56	Male	Ever	Mesothelioma	Epithelioid	40,00	26,86	24,61	29,79	25,84	23,23	30,03
77	Male	Ever	Mesothelioma	Epithelioid	40,00	40,00	25,90	30,53	29,71	26,22	40,00
84	Male	Never	Mesothelioma	Epithelioid	40,00	32,41	27,43	33,63	29,13	25,95	40,00
76	Male	Ever	Mesothelioma	Sarcomatoid	40,00	27,24	25,87	34,29	27,41	24,55	40,00
72	Male	Ever	Mesothelioma	Epithelioid	40,00	31,00	27,50	33,61	28,71	25,83	40,00
77	Male	Ever	Mesothelioma	Sarcomatoid	40,00	40,00	29,76	40,00	31,46	28,92	40,00
85	Male	Ever	Mesothelioma	Epithelioid	40,00	29,22	26,65	31,71	28,01	25,17	40,00
78	Male	Never	Mesothelioma	Epithelioid	40,00	36,07	28,23	40,00	31,50	29,50	40,00
68	Male	Ever	Mesothelioma	Epithelioid	40,00	32,00	28,00	33,48	28,44	25,95	37,90
55	Male	Ever	Asbestos-exposed		40,00	29,57	26,86	34,03	27,76	25,86	38,39
72	Male	Ever	Asbestos-exposed		40,00	28,71	26,25	31,34	26,96	24,95	38,60
69	Male	Never	Asbestos-exposed		40,00	29,02	26,31	30,10	27,56	24,67	35,20
43	Male	Ever	Asbestos-exposed		40,00	27,19	25,88	30,68	26,56	24,58	32,03
75	Male	Never	Asbestos-exposed		40,00	32,12	27,85	31,62	28,73	26,25	40,00
57	Male	Never	Asbestos-exposed		40,00	28,09	26,21	31,43	27,17	24,64	31,77
72	Male	Never	Asbestos-exposed		40,00	29,84	25,44	30,90	27,58	24,75	33,51
71	Male	Never	Asbestos-exposed		40,00	33,16	27,38	33,49	29,37	27,63	40,00
72	Male	Never	Asbestos-exposed		40,00	32,04	29,03	33,41	28,59	26,23	40,00
67	Male	Ever	Asbestos-exposed		40,00	27,26	24,77	29,92	25,96	23,70	31,72
71	Male	Ever	Asbestos-exposed		40,00	27,50	24,88	32,16	26,13	24,23	38,84
53	Male	Never	Asbestos-exposed		40,00	27,29	25,62	30,83	26,08	23,48	33,74
57	Male	Ever	Asbestos-exposed		40,00	32,17	26,19	31,86	28,77	27,47	40,00
79	Male	Ever	Asbestos-exposed		40,00	31,99	27,62	33,34	29,37	27,55	40,00
82	Male	Never	Asbestos-exposed		40,00	30,39	26,26	31,61	28,37	24,97	39,50
77	Male	Ever	Asbestos-exposed		40,00	36,81	26,65	32,50	28,63	26,11	40,00
75	Male	Ever	Asbestos-exposed		40,00	30,78	27,63	31,64	28,51	25,95	39,59
78	Male	Ever	Asbestos-exposed		40,00	27,89	26,06	30,47	26,95	23,88	32,64
81	Male	Ever	Asbestos-exposed		40,00	31,13	26,49	34,80	27,76	26,22	40,00
79	Male	Never	Asbestos-exposed		40,00	32,23	26,40	31,13	28,71	27,37	40,00
68	Male	Ever	Asbestos-exposed		40,00	40,00	30,36	40,00	32,29	29,71	40,00

hsa-let-7g	hsa-miR-1	hsa-miR-100	hsa-miR-101	hsa-miR-103	hsa-miR-105	hsa-miR-106a	hsa-miR-106b	hsa-miR-107	hsa-miR-10a
28,22	40,00	40,00	31,17	34,38	40,00	23,63	28,87	40,00	32,43
31,84	40,00	40,00	40,00	40,00	40,00	25,44	33,42	40,00	40,00
27,09	31,46	33,06	33,40	29,48	40,00	21,85	27,24	40,00	35,78
24,76	33,59	34,93	30,02	26,83	40,00	19,74	26,15	33,26	40,00
26,34	40,00	40,00	31,12	29,22	40,00	21,21	27,57	34,90	40,00
26,71	40,00	40,00	31,56	29,20	40,00	21,76	27,85	34,50	40,00
28,78	40,00	33,95	33,50	31,78	40,00	23,07	29,67	40,00	40,00
26,39	33,73	40,00	29,84	28,62	40,00	20,77	25,92	40,00	32,03
23,91	31,07	30,46	27,48	24,42	40,00	18,64	23,64	30,21	31,33
24,05	30,33	30,18	27,99	25,41	40,00	18,40	23,84	40,00	31,74
26,88	31,90	33,08	30,09	31,16	40,00	20,94	26,10	40,00	40,00
28,06	40,00	40,00	29,15	29,23	40,00	21,74	26,67	34,84	33,30
25,28	32,71	32,63	29,03	27,23	40,00	19,88	25,09	34,90	35,62
27,74	38,88	34,49	28,82	40,00	40,00	21,19	26,95	40,00	33,23
28,58	40,00	40,00	31,22	30,11	40,00	22,50	27,64	40,00	34,58
26,70	33,35	40,00	31,20	28,32	40,00	22,04	26,91	34,35	35,94
28,13	32,92	35,27	32,85	29,07	40,00	22,56	27,60	40,00	40,00
30,43	40,00	40,00	40,00	36,03	40,00	25,46	30,55	40,00	40,00
26,95	40,00	33,07	30,98	28,37	40,00	22,53	27,65	32,59	32,56
32,82	40,00	35,34	40,00	34,86	40,00	24,21	29,66	40,00	32,37
28,31	40,00	34,08	31,97	29,46	40,00	23,14	27,90	40,00	33,27
28,25	32,74	34,11	40,00	30,27	40,00	21,98	27,01	38,56	31,44
28,09	40,00	33,51	31,04	28,12	40,00	21,80	26,48	34,52	33,60
27,12	33,56	31,61	29,90	29,16	40,00	21,48	25,56	40,00	31,85
26,44	34,28	30,95	29,98	26,96	40,00	20,82	24,98	34,92	29,88
29,10	40,00	32,01	31,77	30,20	40,00	22,84	27,72	40,00	31,31
27,01	33,52	31,47	30,06	28,10	40,00	20,63	25,10	40,00	30,42
27,28	34,94	36,46	30,35	29,74	40,00	21,35	26,43	40,00	32,64
28,71	40,00	32,47	37,49	31,18	40,00	23,65	28,11	40,00	33,35
28,69	40,00	34,31	31,35	29,42	40,00	21,64	26,68	40,00	35,33
26,20	30,25	30,92	29,29	28,30	40,00	20,34	25,26	33,98	32,14
26,09	35,57	30,08	29,76	27,76	40,00	19,91	25,06	35,50	30,51
25,84	33,82	34,00	29,56	26,93	40,00	20,00	24,48	34,63	30,35
27,72	33,30	31,90	31,49	30,79	40,00	20,99	25,51	40,00	32,32
29,66	40,00	40,00	32,34	31,62	40,00	22,74	27,20	40,00	33,16
27,70	34,73	35,05	30,95	31,19	40,00	22,30	26,00	40,00	32,29
28,64	40,00	32,78	31,65	31,35	40,00	21,46	26,60	40,00	31,75
29,05	40,00	32,46	31,36	29,46	40,00	22,80	26,70	37,10	30,78
26,63	31,79	31,66	29,99	28,83	40,00	20,98	25,59	35,17	30,97
27,92	34,66	33,75	40,00	30,35	40,00	21,34	25,89	40,00	32,92
28,76	40,00	31,83	29,69	32,61	40,00	22,02	26,53	40,00	31,20
32,08	40,00	40,00	34,41	36,35	40,00	23,75	28,07	40,00	38,15

hsa-miR-10b	hsa-miR-122	hsa-miR-125a-3p	hsa-miR-125a-5p	hsa-miR-125b	hsa-miR-126	hsa-miR-127	hsa-miR-127-5p	hsa-miR-128a
40,00	29,88	40,00	31,29	40,00	23,08	40,00	40,00	32,64
40,00	33,09	40,00	40,00	40,00	25,77	30,61	40,00	40,00
40,00	29,17	40,00	29,18	40,00	21,60	28,99	40,00	30,89
40,00	31,12	40,00	26,63	34,52	19,58	28,74	40,00	28,92
40,00	29,23	40,00	30,28	31,46	21,52	28,59	40,00	30,32
40,00	29,72	40,00	30,54	40,00	22,12	29,07	40,00	31,27
40,00	30,18	40,00	33,60	40,00	23,82	31,02	40,00	32,45
40,00	27,03	40,00	30,44	31,01	21,32	27,45	40,00	29,33
40,00	27,59	32,06	26,22	28,86	18,94	23,46	40,00	27,21
40,00	26,67	31,13	27,99	28,53	19,23	26,96	40,00	27,27
40,00	28,48	40,00	32,65	31,59	21,64	29,44	40,00	29,35
40,00	26,41	40,00	31,72	32,97	21,87	27,98	40,00	31,40
40,00	27,76	33,47	29,50	31,41	20,42	27,63	40,00	28,46
40,00	24,74	40,00	33,19	30,90	22,31	30,17	40,00	30,14
40,00	33,12	40,00	31,51	40,00	22,91	29,98	40,00	30,89
40,00	28,53	40,00	29,92	40,00	21,95	30,96	40,00	31,39
40,00	40,00	40,00	31,57	31,61	22,66	30,09	40,00	30,44
40,00	30,81	40,00	31,49	33,60	25,43	40,00	40,00	32,56
40,00	31,53	40,00	29,70	32,75	22,27	29,97	40,00	30,61
40,00	29,82	40,00	32,98	35,65	24,64	31,07	40,00	32,22
40,00	28,35	40,00	40,00	31,44	23,18	29,03	40,00	30,62
37,25	25,80	40,00	30,81	33,95	21,94	28,43	40,00	30,23
40,00	24,69	40,00	31,32	31,10	21,74	29,08	40,00	31,42
35,89	25,36	33,45	29,18	31,32	20,48	27,77	40,00	29,61
31,22	25,03	40,00	30,48	28,92	20,50	28,30	40,00	29,59
36,56	25,64	40,00	32,15	30,50	22,62	30,35	39,55	32,10
30,93	23,90	40,00	30,07	30,05	20,57	28,21	40,00	29,73
40,00	27,04	32,35	31,07	29,84	20,89	26,79	40,00	29,45
40,00	30,93	40,00	32,34	34,45	22,66	28,11	40,00	32,99
34,02	25,04	40,00	31,10	31,33	21,05	28,27	40,00	30,69
35,36	25,97	34,86	28,92	29,40	19,26	26,66	40,00	28,61
32,76	26,33	34,29	28,20	30,18	19,26	26,95	40,00	29,83
38,34	26,60	40,00	27,78	31,16	19,04	26,12	40,00	29,02
31,70	25,77	40,00	30,88	30,82	21,23	26,69	40,00	30,01
36,85	26,80	40,00	40,00	32,01	22,91	29,08	40,00	31,11
39,42	27,32	33,02	29,60	31,20	21,30	26,66	40,00	29,51
38,40	25,65	35,04	30,02	31,23	21,35	29,47	40,00	31,31
36,00	25,36	34,60	32,54	30,97	21,94	27,96	40,00	32,52
35,84	24,47	31,63	28,73	28,63	20,01	29,55	40,00	28,63
37,44	26,00	33,42	28,24	31,52	20,98	26,88	40,00	29,17
34,69	27,03	40,00	33,09	29,72	22,07	28,68	40,00	30,20
37,90	26,12	40,00	32,83	33,02	23,49	30,69	40,00	32,37

hsa-miR-129	hsa-miR-130a	hsa-miR-130b	hsa-miR-132	hsa-miR-133a	hsa-miR-133b	hsa-miR-135a	hsa-miR-135b	hsa-miR-136	hsa-miR-138
40,00	28,69	29,48	29,98	28,43	34,48	40,00	40,00	40,00	40,00
40,00	34,00	34,64	33,84	33,11	40,00	40,00	40,00	40,00	40,00
40,00	29,19	29,41	28,72	27,35	31,20	40,00	40,00	40,00	40,00
40,00	26,80	28,98	27,00	26,74	33,87	33,79	36,92	40,00	40,00
40,00	27,32	29,43	28,61	30,42	35,84	40,00	40,00	40,00	40,00
40,00	27,82	29,13	29,03	29,99	34,22	40,00	40,00	40,00	32,67
40,00	29,88	30,68	30,66	31,66	40,00	40,00	40,00	40,00	40,00
40,00	24,25	26,16	27,18	27,55	32,51	40,00	40,00	40,00	40,00
40,00	21,73	24,39	24,76	26,55	31,89	31,98	39,01	37,53	31,34
40,00	22,83	24,39	25,38	24,32	29,52	40,00	32,73	40,00	31,54
40,00	25,35	26,48	27,31	26,72	31,86	40,00	40,00	40,00	31,92
40,00	26,49	27,56	29,11	29,71	36,65	33,83	40,00	40,00	40,00
40,00	23,65	25,44	27,30	28,46	31,34	32,63	40,00	40,00	31,50
40,00	25,09	26,16	26,76	28,08	32,44	33,76	40,00	40,00	33,18
40,00	26,60	29,26	31,16	30,09	33,91	40,00	40,00	40,00	40,00
40,00	26,60	28,36	29,53	29,46	32,92	40,00	40,00	40,00	40,00
40,00	26,52	28,64	30,71	29,40	33,39	40,00	40,00	40,00	32,63
40,00	29,96	30,34	35,34	30,91	40,00	40,00	40,00	40,00	40,00
40,00	27,50	29,14	31,72	28,24	33,43	40,00	40,00	40,00	40,00
40,00	28,36	31,29	32,13	29,39	34,41	40,00	40,00	40,00	40,00
40,00	27,82	29,48	31,22	30,65	40,00	40,00	40,00	40,00	40,00
40,00	28,37	28,67	27,21	28,64	32,60	40,00	40,00	40,00	32,66
40,00	28,21	29,15	28,38	30,83	40,00	40,00	40,00	40,00	40,00
40,00	25,40	26,67	26,31	28,95	32,65	34,73	40,00	40,00	40,00
40,00	26,48	27,41	27,04	28,58	31,23	32,88	40,00	38,67	40,00
40,00	29,00	29,46	28,60	31,04	40,00	40,00	40,00	40,00	40,00
40,00	26,66	27,99	26,96	29,66	34,03	33,94	40,00	40,00	40,00
40,00	25,96	27,23	26,26	29,02	32,66	40,00	40,00	40,00	32,45
40,00	27,66	30,59	29,77	29,23	33,20	40,00	40,00	40,00	40,00
40,00	27,91	27,92	27,54	28,28	32,84	40,00	40,00	40,00	40,00
40,00	24,56	25,63	25,69	25,51	29,01	40,00	40,00	40,00	40,00
40,00	25,33	26,95	26,71	26,13	28,73	40,00	40,00	40,00	30,85
40,00	25,42	26,57	25,94	26,99	31,22	40,00	35,38	40,00	33,45
40,00	27,16	28,64	26,75	25,98	29,83	40,00	40,00	40,00	40,00
40,00	28,87	30,26	29,75	30,43	37,72	40,00	40,00	40,00	40,00
40,00	25,87	26,98	27,17	30,09	34,49	40,00	40,00	40,00	32,88
40,00	28,17	28,10	26,78	27,96	32,18	40,00	40,00	40,00	40,00
40,00	26,95	28,68	28,02	29,32	32,50	40,00	40,00	36,72	31,40
40,00	25,28	26,78	26,15	26,82	29,91	40,00	40,00	40,00	31,71
40,00	27,23	27,65	26,39	26,10	30,25	40,00	39,09	40,00	33,93
40,00	27,34	29,22	28,54	28,11	31,99	40,00	40,00	38,77	40,00
40,00	28,95	29,78	28,36	30,19	40,00	40,00	40,00	40,00	40,00

hsa-miR-139-3p	hsa-miR-139-5p	hsa-miR-140-3p	hsa-miR-141	hsa-miR-142-3p	hsa-miR-142-5p	hsa-miR-143	hsa-miR-145	hsa-miR-146a
33,79	28,32	31,66	40,00	27,15	31,51	30,02	29,05	21,13
40,00	31,31	34,28	40,00	29,15	40,00	40,00	31,99	24,15
29,97	26,96	31,90	40,00	25,28	30,37	30,16	27,21	20,54
27,71	23,97	28,85	40,00	23,39	29,50	30,29	26,96	19,16
29,51	26,12	30,96	40,00	25,05	29,74	30,09	27,80	20,42
31,32	25,76	31,37	40,00	25,11	30,96	31,27	27,97	21,41
31,99	28,44	32,46	40,00	28,09	31,34	34,51	29,92	22,43
40,00	25,72	29,03	35,20	24,36	28,39	26,11	24,87	20,53
28,54	22,98	27,69	32,95	20,93	25,56	25,49	23,94	18,01
28,79	23,53	26,82	31,51	22,41	26,80	27,44	24,64	18,33
31,35	25,50	28,79	34,51	25,07	28,49	30,30	27,96	20,27
32,66	25,88	31,15	34,36	25,02	29,98	29,76	27,26	21,18
28,49	23,45	29,61	34,02	22,68	27,89	29,92	25,98	20,30
29,96	26,17	29,49	33,66	27,40	29,71	29,95	29,28	20,34
33,04	27,11	30,94	40,00	25,12	29,72	29,94	27,94	21,95
34,39	26,13	31,93	34,55	23,68	29,57	30,10	27,05	22,35
32,31	26,37	31,63	35,04	23,91	29,88	29,98	27,44	22,32
34,73	28,76	32,96	40,00	27,40	34,58	32,69	29,59	24,55
30,37	25,85	32,05	40,00	24,34	29,67	32,26	27,00	22,14
34,63	28,51	32,42	40,00	27,57	32,95	32,19	28,10	23,55
40,00	26,64	32,34	40,00	24,30	29,21	31,58	28,17	23,30
30,45	26,82	33,13	33,04	25,57	30,48	31,12	27,02	21,43
31,23	26,91	33,18	40,00	24,24	30,84	29,25	25,89	22,37
29,12	25,15	31,81	40,00	23,71	29,00	31,21	26,70	19,78
39,73	24,96	31,03	32,82	22,62	28,92	28,13	24,96	21,62
32,56	26,82	34,43	40,00	25,38	31,90	30,97	28,27	22,97
31,01	25,68	31,24	34,32	23,30	29,12	30,02	25,92	20,98
34,87	27,16	31,24	34,87	24,60	29,04	29,32	27,15	20,24
40,00	27,81	33,39	40,00	26,98	33,37	29,98	26,34	22,10
31,47	25,14	31,63	40,00	25,53	30,41	30,08	26,62	20,01
31,25	25,08	30,34	35,58	22,75	27,91	27,89	23,96	18,84
40,00	25,75	29,48	40,00	23,62	28,90	26,44	23,50	21,80
29,40	24,55	30,34	34,00	22,63	27,86	27,75	24,59	19,11
30,44	25,26	31,33	34,51	26,17	30,09	27,99	25,94	19,59
34,54	26,83	33,57	40,00	26,19	31,43	30,79	27,56	22,51
29,02	26,24	33,24	40,00	24,80	29,48	28,02	25,27	20,49
33,18	26,02	31,89	40,00	26,25	30,93	28,09	25,41	19,54
32,32	26,44	34,06	40,00	24,63	30,68	31,56	27,65	21,40
29,05	25,01	30,98	33,39	23,05	28,10	27,71	25,44	18,83
28,06	24,94	32,03	34,40	25,90	29,85	29,88	26,27	19,25
37,71	27,11	32,96	34,57	25,81	33,37	29,97	27,35	22,79
32,98	27,77	34,73	40,00	29,13	35,20	31,90	29,09	21,43

hsa-miR-146b	hsa-miR-146b-3p	hsa-miR-147	hsa-miR-147b	hsa-miR-148a	hsa-miR-148b	hsa-miR-149	hsa-miR-150	hsa-miR-152	hsa-miR-154
26,75	40,00	40,00	40,00	30,90	31,13	40,00	27,31	29,02	40,00
29,49	40,00	40,00	40,00	30,94	32,10	40,00	28,10	30,89	40,00
25,38	40,00	40,00	40,00	29,28	30,17	40,00	25,22	28,97	40,00
23,22	40,00	40,00	40,00	30,36	30,01	40,00	25,11	27,62	40,00
25,32	40,00	40,00	40,00	29,89	31,05	40,00	26,45	28,32	40,00
25,74	40,00	40,00	40,00	30,43	30,79	40,00	25,73	28,54	40,00
27,61	40,00	40,00	40,00	32,09	32,07	40,00	25,71	29,74	40,00
25,17	40,00	40,00	40,00	27,25	30,12	40,00	23,06	27,18	37,18
22,21	31,02	40,00	40,00	25,43	27,84	40,00	23,36	24,74	31,83
22,58	40,00	40,00	40,00	26,10	28,12	40,00	21,08	25,58	34,95
25,42	40,00	40,00	40,00	28,15	30,00	40,00	24,09	26,96	40,00
25,98	40,00	40,00	40,00	28,01	31,33	40,00	26,02	28,16	40,00
24,36	33,58	40,00	40,00	26,51	29,00	40,00	23,99	26,42	32,98
25,55	37,99	40,00	40,00	27,94	30,10	40,00	24,38	26,90	40,00
26,45	40,00	40,00	40,00	29,26	32,49	40,00	26,85	29,01	40,00
26,15	40,00	40,00	40,00	28,63	30,73	40,00	27,32	28,05	40,00
25,99	32,75	40,00	40,00	28,60	30,50	40,00	28,44	28,41	40,00
28,26	40,00	40,00	40,00	40,00	35,05	40,00	26,52	30,29	40,00
25,93	40,00	40,00	40,00	29,24	33,31	40,00	26,14	29,41	40,00
27,60	40,00	40,00	40,00	31,92	32,65	40,00	26,82	28,24	40,00
27,23	32,44	40,00	40,00	29,61	31,80	40,00	27,73	29,68	40,00
25,96	40,00	40,00	40,00	28,87	31,02	40,00	24,42	28,67	34,19
26,39	40,00	40,00	40,00	29,12	30,66	40,00	25,21	28,90	37,57
24,74	40,00	40,00	40,00	26,97	29,45	40,00	23,46	27,08	40,00
25,25	40,00	40,00	40,00	27,79	29,56	40,00	24,38	27,28	40,00
27,02	40,00	40,00	40,00	29,26	31,81	40,00	24,20	29,81	40,00
24,89	35,54	40,00	40,00	27,77	29,86	40,00	22,92	27,03	40,00
25,04	40,00	40,00	40,00	27,88	29,81	40,00	24,29	26,59	39,26
26,89	40,00	40,00	40,00	30,46	31,30	40,00	25,50	28,80	40,00
25,16	38,92	40,00	40,00	29,30	29,80	40,00	25,11	27,54	39,58
23,71	40,00	40,00	40,00	27,34	27,99	40,00	23,78	25,78	40,00
24,41	33,01	40,00	40,00	27,14	28,86	40,00	24,70	27,79	35,29
23,51	40,00	40,00	40,00	26,80	28,78	40,00	23,68	26,57	40,00
24,58	33,53	40,00	40,00	28,45	29,34	39,65	23,81	27,26	40,00
27,48	36,39	40,00	40,00	30,39	31,51	40,00	24,26	28,94	40,00
25,29	40,00	40,00	40,00	27,98	28,69	40,00	24,55	26,35	38,16
25,16	40,00	40,00	40,00	28,39	30,14	40,00	24,55	27,95	40,00
26,35	40,00	40,00	40,00	28,46	30,78	40,00	24,28	27,84	40,00
24,01	40,00	40,00	40,00	27,74	28,40	40,00	24,05	25,77	40,00
24,82	40,00	40,00	40,00	27,70	29,20	40,00	24,84	26,27	38,73
26,18	40,00	40,00	40,00	29,31	32,29	40,00	24,42	28,42	40,00
27,34	40,00	40,00	40,00	30,30	31,35	40,00	25,00	28,58	40,00

hsa-miR-155	hsa-miR-15a	hsa-miR-15b	hsa-miR-16	hsa-miR-17	hsa-miR-181a	hsa-miR-181c	hsa-miR-182	hsa-miR-183	hsa-miR-184
29,44	40,00	31,95	24,69	23,57	32,32	37,45	33,98	40,00	40,00
32,04	40,00	33,72	26,30	25,66	40,00	40,00	40,00	40,00	40,00
27,61	40,00	28,34	23,65	21,51	31,14	32,49	35,88	40,00	40,00
25,13	33,21	25,87	21,57	19,81	31,29	38,76	32,24	40,00	40,00
27,03	31,76	27,87	22,54	21,11	31,16	30,65	40,00	40,00	40,00
27,43	31,68	28,07	22,85	21,63	31,51	33,69	40,00	40,00	40,00
29,87	35,05	31,80	24,35	23,08	35,46	40,00	40,00	40,00	40,00
27,85	40,00	27,02	20,20	20,66	29,23	34,63	34,34	40,00	40,00
25,28	29,19	24,15	18,51	18,47	26,89	26,91	34,87	40,00	40,00
26,24	29,10	24,47	18,38	18,21	27,85	34,32	40,00	37,33	40,00
28,77	32,00	28,11	20,57	20,81	30,59	35,75	40,00	40,00	40,00
29,76	31,97	27,78	22,15	21,47	32,06	33,90	35,23	40,00	40,00
27,45	30,88	26,21	20,18	19,92	28,97	30,05	40,00	40,00	33,28
28,79	40,00	30,88	20,82	21,04	31,50	40,00	40,00	36,11	40,00
29,49	40,00	29,05	23,74	22,70	32,28	40,00	40,00	40,00	40,00
28,67	33,90	27,22	22,48	22,00	30,85	40,00	33,89	40,00	40,00
29,23	40,00	28,48	23,32	22,66	31,42	40,00	40,00	40,00	40,00
32,18	40,00	33,00	24,99	25,42	32,97	40,00	40,00	40,00	40,00
28,61	32,20	27,90	22,81	22,53	31,43	40,00	36,54	40,00	40,00
31,74	40,00	32,56	23,84	24,66	32,98	33,95	40,00	40,00	40,00
29,88	36,25	28,46	23,72	23,07	32,63	37,03	40,00	40,00	40,00
29,28	38,70	27,63	22,53	21,94	30,51	32,64	40,00	40,00	40,00
29,85	37,80	27,04	21,18	21,63	31,91	32,06	40,00	36,33	40,00
28,96	39,05	26,13	21,77	21,44	29,37	30,85	40,00	40,00	40,00
29,62	33,36	24,81	21,56	20,73	30,53	32,59	40,00	40,00	33,09
30,05	40,00	28,50	22,56	22,80	31,65	34,91	40,00	40,00	40,00
28,80	34,74	26,05	20,32	20,55	30,17	29,61	36,01	40,00	40,00
30,69	35,89	26,57	21,58	21,12	30,31	30,76	40,00	40,00	40,00
30,33	40,00	28,49	24,53	23,64	30,97	34,30	40,00	40,00	40,00
29,62	36,36	26,49	22,94	21,53	30,89	33,26	40,00	40,00	34,20
28,12	37,48	24,65	20,96	20,11	28,01	30,41	40,00	40,00	32,24
28,46	39,79	25,01	20,93	19,82	30,52	32,56	40,00	38,90	32,15
27,40	37,60	24,31	20,98	19,76	29,20	30,56	33,92	40,00	40,00
28,97	40,00	26,67	21,53	20,72	30,64	31,91	35,55	39,81	33,48
30,57	34,40	28,11	22,82	22,58	31,59	33,74	40,00	40,00	40,00
29,68	40,00	26,79	22,76	22,05	30,78	32,94	40,00	40,00	34,08
28,88	40,00	27,92	22,22	21,42	30,46	33,94	40,00	38,03	40,00
30,03	37,80	27,56	22,92	22,62	32,09	33,79	40,00	37,70	40,00
28,78	37,20	25,75	21,35	20,98	29,29	29,81	40,00	40,00	28,09
29,25	37,75	26,10	22,18	21,18	30,24	31,75	40,00	40,00	40,00
32,12	40,00	28,06	21,91	22,25	32,77	40,00	36,53	40,00	40,00
30,57	40,00	30,48	24,17	23,67	31,51	34,86	40,00	40,00	40,00

hsa-miR-185	hsa-miR-186	hsa-miR-188-3p	hsa-miR-18a	hsa-miR-18b	hsa-miR-190	hsa-miR-191	hsa-miR-192	hsa-miR-193a-3p
30,60	26,48	40,00	33,07	40,00	40,00	21,36	30,48	40,00
32,63	30,13	40,00	39,77	40,00	40,00	23,95	35,32	40,00
28,46	25,62	40,00	30,15	40,00	40,00	20,01	29,99	40,00
26,93	23,15	40,00	30,10	40,00	40,00	17,49	29,55	40,00
28,49	24,94	40,00	29,84	36,07	40,00	19,21	30,40	40,00
28,24	25,67	40,00	30,01	40,00	40,00	19,90	30,47	40,00
30,00	27,44	40,00	31,30	40,00	40,00	22,23	30,91	40,00
26,10	24,53	40,00	30,54	40,00	40,00	20,99	28,50	40,00
23,94	21,78	40,00	25,74	31,12	35,22	17,71	26,94	40,00
23,97	22,18	40,00	27,79	40,00	33,76	17,96	26,80	40,00
25,92	24,36	40,00	30,59	40,00	40,00	21,19	28,25	40,00
27,26	24,98	40,00	29,55	40,00	33,73	20,89	28,36	40,00
25,60	23,91	40,00	27,95	33,38	32,20	19,30	28,66	40,00
26,87	25,00	40,00	30,68	40,00	40,00	22,18	27,96	38,90
27,25	26,23	40,00	32,31	40,00	40,00	21,57	30,02	40,00
28,22	25,87	40,00	29,31	40,00	40,00	20,73	30,44	40,00
27,82	25,48	40,00	31,73	40,00	40,00	20,98	30,24	40,00
30,22	28,24	40,00	36,30	40,00	40,00	23,32	32,80	40,00
27,95	25,69	40,00	30,18	40,00	40,00	20,15	30,29	40,00
28,72	27,42	40,00	31,66	40,00	40,00	22,95	30,91	40,00
28,22	26,18	40,00	29,67	37,69	33,37	21,41	30,09	40,00
28,07	25,43	40,00	28,77	37,89	40,00	22,20	29,14	40,00
28,15	26,15	40,00	28,37	38,36	40,00	22,10	28,27	40,00
27,21	24,74	40,00	28,73	36,97	40,00	21,04	28,25	40,00
26,48	25,58	40,00	27,31	33,05	33,69	20,91	28,31	40,00
30,04	27,11	40,00	30,19	40,00	40,00	23,74	29,46	40,00
26,99	25,25	40,00	27,62	33,35	40,00	21,23	26,63	40,00
27,27	26,33	40,00	29,03	35,28	40,00	21,76	28,68	40,00
29,64	27,18	40,00	31,01	40,00	40,00	23,21	31,86	40,00
28,00	24,90	40,00	29,64	37,48	40,00	20,85	28,31	40,00
26,03	24,16	40,00	27,32	33,75	40,00	20,11	28,20	40,00
26,14	24,93	40,00	27,14	34,28	40,00	20,31	27,89	40,00
26,11	23,21	40,00	27,52	32,45	34,46	19,36	27,80	40,00
26,97	24,28	40,00	28,76	34,68	40,00	20,41	27,48	40,00
29,07	26,54	40,00	31,17	33,45	40,00	22,92	28,98	40,00
27,21	25,24	40,00	29,43	40,00	40,00	21,65	28,64	40,00
27,62	25,08	40,00	29,74	37,48	40,00	20,97	28,64	40,00
27,94	26,31	40,00	29,45	40,00	40,00	22,23	29,64	40,00
26,84	24,27	40,00	28,11	36,02	33,47	20,46	28,07	40,00
26,89	23,93	40,00	30,09	38,19	40,00	20,70	28,36	40,00
27,93	26,85	40,00	29,30	33,45	40,00	22,90	28,98	40,00
29,64	27,25	40,00	31,37	40,00	40,00	24,25	29,63	40,00

hsa-miR-193a-5p	hsa-miR-193b	hsa-miR-194	hsa-miR-195	hsa-miR-196b	hsa-miR-197	hsa-miR-198	hsa-miR-199a	hsa-miR-199a-3p
35,78	32,99	40,00	30,50	40,00	26,18	40,00	40,00	26,40
35,63	40,00	40,00	32,14	33,30	29,60	40,00	40,00	29,67
33,25	32,66	32,46	29,65	33,22	25,13	40,00	33,90	25,07
31,40	40,00	31,88	26,88	30,62	23,78	40,00	31,36	23,24
31,18	35,14	40,00	28,54	40,00	25,21	40,00	40,00	24,79
31,89	35,75	32,28	28,48	40,00	26,22	40,00	40,00	25,56
32,58	33,76	40,00	29,76	40,00	27,65	40,00	40,00	27,56
29,62	30,51	32,47	26,25	34,84	25,19	40,00	30,85	24,83
30,23	30,74	28,79	24,33	30,95	22,67	40,00	30,02	23,98
29,73	30,10	30,66	24,19	31,49	23,62	40,00	29,20	22,27
32,78	31,92	33,42	26,45	40,00	25,33	40,00	33,75	24,46
30,93	29,52	32,68	28,36	40,00	25,96	40,00	40,00	25,69
31,05	30,74	30,71	26,63	30,59	23,92	40,00	30,76	24,13
28,83	29,46	29,20	26,90	40,00	25,72	40,00	31,99	26,59
33,47	40,00	40,00	29,30	40,00	27,23	40,00	35,28	26,68
36,41	30,09	35,27	27,87	35,01	26,68	40,00	33,59	25,97
35,68	34,62	33,55	28,78	40,00	27,17	40,00	40,00	26,27
37,02	40,00	40,00	31,50	40,00	28,14	40,00	40,00	29,47
35,80	30,94	40,00	28,83	40,00	25,85	40,00	33,46	26,11
36,01	33,99	40,00	29,76	40,00	27,39	40,00	40,00	28,84
33,55	31,02	32,72	29,09	33,54	26,72	40,00	33,66	27,39
29,79	29,90	40,00	27,63	33,88	25,69	40,00	36,41	25,73
29,46	31,36	31,32	27,37	33,43	26,69	40,00	32,30	25,59
28,67	29,37	31,72	27,58	34,41	24,53	40,00	31,93	23,91
28,14	28,94	31,07	26,69	33,80	25,08	40,00	31,58	23,71
30,54	30,36	31,99	27,96	40,00	27,67	40,00	40,00	25,87
27,28	27,39	31,36	25,88	40,00	25,27	40,00	31,04	23,83
29,46	30,27	40,00	26,94	40,00	25,08	40,00	32,58	23,93
30,58	31,18	33,52	29,23	32,67	26,74	40,00	40,00	26,83
29,78	28,73	31,87	28,19	33,66	25,59	40,00	33,06	25,66
29,67	29,92	30,78	26,29	32,96	24,72	40,00	31,37	23,38
30,03	30,33	30,84	25,98	31,16	25,51	37,16	29,86	23,53
29,46	28,61	30,18	26,39	31,39	23,75	40,00	31,08	23,46
29,82	29,36	30,79	26,90	40,00	24,59	40,00	35,04	26,94
30,16	30,37	34,04	28,75	40,00	26,20	40,00	35,91	26,09
29,45	30,99	31,01	27,97	31,96	24,43	40,00	33,63	24,53
30,29	31,11	31,82	27,81	33,69	24,59	40,00	34,97	24,77
28,98	29,62	32,66	28,24	33,58	26,04	40,00	31,96	26,13
28,35	29,10	30,62	27,24	34,91	24,03	40,00	30,25	23,64
30,94	30,11	32,26	27,59	33,68	23,54	40,00	34,24	24,64
30,26	29,94	32,20	26,49	40,00	26,21	40,00	32,93	25,62
30,50	31,26	34,50	29,53	40,00	27,05	40,00	32,46	28,40

hsa-miR-199b	hsa-miR-19a	hsa-miR-19b	hsa-miR-200a	hsa-miR-200b	hsa-miR-200c	hsa-miR-202	hsa-miR-203	hsa-miR-204	hsa-miR-205
40,00	26,99	22,40	40,00	40,00	33,20	40,00	40,00	40,00	40,00
40,00	29,89	25,06	40,00	40,00	40,00	40,00	40,00	40,00	40,00
35,29	25,31	20,54	40,00	31,84	33,32	40,00	40,00	40,00	40,00
34,30	24,54	19,34	34,14	35,71	29,96	40,00	40,00	32,95	40,00
40,00	25,84	21,01	40,00	40,00	30,13	40,00	40,00	40,00	40,00
37,43	26,60	21,61	40,00	40,00	31,98	40,00	40,00	31,18	40,00
40,00	27,36	22,55	40,00	40,00	40,00	40,00	40,00	34,37	40,00
32,27	27,02	21,66	40,00	36,65	31,45	40,00	30,08	33,29	40,00
32,29	23,54	18,92	40,00	33,76	27,97	40,00	40,00	28,21	40,00
32,01	24,23	19,13	32,87	33,31	29,90	40,00	30,23	29,30	32,34
33,89	25,89	21,28	40,00	37,83	33,31	35,44	40,00	33,69	40,00
32,96	25,95	21,19	40,00	40,00	33,75	40,00	40,00	40,00	40,00
33,30	25,31	20,77	32,82	33,95	29,37	40,00	40,00	32,08	40,00
40,00	26,14	21,36	40,00	40,00	40,00	40,00	33,21	32,40	40,00
37,59	27,40	22,17	40,00	40,00	34,05	40,00	32,95	34,52	40,00
35,22	27,24	22,17	40,00	40,00	32,27	40,00	40,00	30,51	40,00
31,99	27,35	21,88	40,00	40,00	31,69	40,00	40,00	33,82	40,00
40,00	29,68	24,90	40,00	40,00	40,00	40,00	40,00	40,00	40,00
35,34	27,87	22,32	40,00	40,00	32,36	40,00	40,00	33,53	33,03
40,00	29,62	24,32	40,00	40,00	40,00	40,00	40,00	33,38	40,00
40,00	27,20	22,34	40,00	40,00	33,93	40,00	33,48	33,68	40,00
35,47	26,69	21,44	39,77	36,22	35,87	40,00	40,00	32,12	40,00
35,70	27,01	20,99	40,00	40,00	33,29	32,85	34,17	31,12	40,00
35,62	25,90	20,14	40,00	36,18	31,82	40,00	40,00	29,41	40,00
32,28	25,59	20,21	33,10	33,32	30,63	37,20	31,34	30,87	33,23
40,00	27,63	22,20	40,00	40,00	32,86	40,00	40,00	33,70	40,00
32,47	25,08	19,79	40,00	38,52	31,68	36,84	40,00	28,88	40,00
35,00	25,97	20,64	40,00	36,52	31,57	38,78	32,38	30,98	40,00
40,00	28,08	22,77	40,00	39,56	40,00	33,38	40,00	36,33	40,00
40,00	27,00	21,01	36,01	35,63	31,44	31,83	40,00	33,85	40,00
32,69	24,50	19,12	40,00	33,25	29,61	40,00	31,57	30,81	40,00
32,76	25,05	19,65	38,36	34,43	29,53	34,90	40,00	29,68	33,27
32,97	24,93	19,33	40,00	33,98	28,99	40,00	34,47	28,02	40,00
40,00	25,95	20,38	40,00	35,03	31,43	40,00	40,00	31,41	32,65
40,00	28,03	22,06	40,00	40,00	36,20	38,97	31,56	36,02	40,00
34,61	27,13	21,33	37,88	34,90	35,80	39,46	34,53	35,71	40,00
37,76	26,60	20,82	39,33	36,29	32,27	40,00	40,00	32,85	40,00
34,34	27,61	21,64	38,88	40,00	32,52	35,79	40,00	31,88	40,00
33,49	25,46	19,78	40,00	35,47	30,39	37,86	32,73	31,54	40,00
37,45	25,98	19,98	36,48	34,94	33,74	39,15	31,64	36,96	40,00
34,81	27,21	21,69	40,00	37,42	34,13	30,70	33,91	30,74	35,25
39,44	29,01	22,89	40,00	40,00	37,08	40,00	32,09	29,87	33,12

hsa-miR-208	hsa-miR-208b	hsa-miR-20a	hsa-miR-20b	hsa-miR-21	hsa-miR-210	hsa-miR-211	hsa-miR-212	hsa-miR-214	hsa-miR-215
40,00	40,00	24,95	27,42	27,41	32,18	40,00	36,39	40,00	40,00
40,00	40,00	27,28	29,49	29,07	34,46	40,00	40,00	37,03	40,00
40,00	40,00	22,77	25,36	25,93	31,29	40,00	34,14	40,00	34,71
40,00	40,00	21,10	23,57	24,98	29,74	35,36	31,74	40,00	40,00
40,00	40,00	22,45	25,06	25,53	32,60	40,00	32,78	37,32	40,00
40,00	40,00	23,24	25,46	26,68	32,97	40,00	34,12	40,00	34,51
40,00	40,00	24,14	26,47	26,84	40,00	40,00	40,00	35,82	40,00
40,00	40,00	22,07	25,02	24,67	29,45	33,41	34,44	31,62	40,00
40,00	40,00	19,83	22,44	21,98	26,51	40,00	30,86	31,48	32,02
40,00	40,00	19,45	22,75	22,73	27,43	33,85	30,88	31,33	40,00
40,00	40,00	21,87	24,92	24,20	30,86	32,56	40,00	33,53	40,00
40,00	40,00	22,82	25,96	24,60	40,00	40,00	32,60	40,00	40,00
40,00	40,00	20,99	23,92	22,86	29,89	40,00	31,75	33,51	32,94
40,00	40,00	21,85	25,16	24,26	32,91	40,00	32,46	33,82	34,36
40,00	40,00	23,96	27,05	25,89	33,08	40,00	40,00	37,45	40,00
40,00	40,00	22,94	26,00	24,89	40,00	40,00	34,62	36,72	40,00
40,00	40,00	23,99	26,90	25,47	40,00	40,00	40,00	40,00	40,00
40,00	40,00	26,83	29,63	28,94	40,00	33,91	40,00	40,00	40,00
40,00	40,00	24,57	26,81	25,48	40,00	40,00	33,04	35,53	40,00
40,00	40,00	26,75	28,91	27,38	38,45	40,00	40,00	38,35	32,87
40,00	40,00	24,12	26,58	25,75	36,15	40,00	31,87	40,00	35,67
40,00	40,00	23,46	25,70	24,64	33,60	39,02	31,12	35,20	32,71
40,00	40,00	22,93	25,51	24,71	36,68	40,00	33,52	33,81	32,28
40,00	40,00	23,39	25,86	23,51	33,55	40,00	40,00	32,66	32,79
40,00	40,00	21,85	24,50	23,07	31,50	33,85	32,68	32,09	30,91
40,00	40,00	23,77	26,49	25,81	39,95	37,19	35,90	38,44	40,00
40,00	40,00	21,72	24,04	23,69	31,73	40,00	40,00	32,40	32,94
40,00	40,00	22,55	25,09	23,33	32,01	40,00	33,07	35,05	40,00
40,00	40,00	25,28	27,22	25,99	38,64	38,70	40,00	40,00	40,00
40,00	40,00	24,18	25,98	25,09	31,00	38,79	35,47	33,69	37,69
40,00	40,00	21,92	24,54	22,76	29,57	40,00	30,84	36,12	33,92
40,00	40,00	21,07	23,51	23,64	30,05	35,31	33,56	32,46	31,22
40,00	40,00	22,04	24,30	23,17	29,22	39,18	36,55	33,39	33,52
40,00	40,00	22,88	25,14	24,82	32,34	33,07	35,95	32,01	33,88
40,00	40,00	24,21	26,33	25,68	35,89	31,82	40,00	40,00	30,26
40,00	40,00	23,94	26,04	23,83	32,40	40,00	31,80	34,17	38,58
40,00	32,42	23,85	25,51	24,98	31,38	40,00	33,15	37,80	34,69
40,00	40,00	25,07	27,05	24,40	33,89	40,00	38,01	34,42	36,18
40,00	40,00	22,96	25,29	22,64	28,44	40,00	33,32	33,51	35,13
40,00	40,00	23,96	25,83	24,46	31,08	38,25	31,35	32,46	36,72
40,00	40,00	22,76	25,86	24,97	36,05	31,76	34,45	33,31	32,68
40,00	40,00	26,53	28,23	26,29	37,89	40,00	40,00	34,58	40,00

hsa-miR-216a	hsa-miR-216b	hsa-miR-217	hsa-miR-218	hsa-miR-219	hsa-miR-219-1-3p	hsa-miR-219-2-3p	hsa-miR-22	hsa-miR-220
40,00	40,00	40,00	40,00	40,00	40,00	40,00	40,00	40,00
40,00	40,00	40,00	40,00	40,00	40,00	40,00	40,00	40,00
40,00	40,00	40,00	40,00	40,00	40,00	40,00	40,00	40,00
40,00	40,00	40,00	40,00	33,18	40,00	40,00	40,00	40,00
40,00	40,00	40,00	40,00	40,00	40,00	40,00	40,00	40,00
40,00	40,00	40,00	40,00	40,00	40,00	40,00	40,00	40,00
40,00	40,00	40,00	40,00	40,00	40,00	40,00	40,00	40,00
40,00	40,00	40,00	33,62	40,00	40,00	40,00	29,08	40,00
40,00	40,00	40,00	40,00	40,00	40,00	40,00	25,43	40,00
40,00	40,00	40,00	40,00	40,00	40,00	40,00	25,89	40,00
40,00	40,00	40,00	40,00	40,00	40,00	40,00	29,12	40,00
40,00	40,00	40,00	33,39	40,00	40,00	40,00	40,00	40,00
40,00	40,00	40,00	40,00	40,00	40,00	40,00	28,29	40,00
40,00	40,00	40,00	40,00	40,00	40,00	40,00	28,71	40,00
40,00	40,00	40,00	40,00	40,00	35,69	40,00	40,00	40,00
40,00	40,00	40,00	40,00	40,00	40,00	40,00	40,00	40,00
40,00	40,00	40,00	40,00	40,00	40,00	40,00	40,00	40,00
40,00	40,00	40,00	40,00	40,00	40,00	40,00	40,00	40,00
40,00	40,00	40,00	33,48	40,00	40,00	40,00	40,00	40,00
40,00	40,00	40,00	34,38	40,00	40,00	40,00	40,00	40,00
40,00	40,00	40,00	40,00	40,00	40,00	40,00	39,55	40,00
40,00	40,00	40,00	40,00	40,00	40,00	40,00	32,23	40,00
40,00	40,00	40,00	31,89	40,00	40,00	40,00	32,31	40,00
40,00	40,00	40,00	30,39	40,00	40,00	40,00	28,89	40,00
40,00	40,00	40,00	32,99	40,00	40,00	40,00	26,80	40,00
40,00	40,00	40,00	40,00	40,00	40,00	40,00	35,10	40,00
40,00	40,00	40,00	33,25	40,00	40,00	40,00	28,32	40,00
40,00	40,00	40,00	33,95	40,00	40,00	40,00	27,64	40,00
40,00	40,00	40,00	40,00	40,00	40,00	40,00	30,42	40,00
40,00	34,87	40,00	40,00	40,00	40,00	40,00	29,36	40,00
40,00	40,00	40,00	40,00	40,00	40,00	40,00	26,16	40,00
40,00	40,00	40,00	40,00	40,00	40,00	40,00	25,76	40,00
40,00	40,00	40,00	40,00	40,00	40,00	40,00	28,21	40,00
40,00	40,00	40,00	32,64	40,00	40,00	40,00	28,29	40,00
40,00	40,00	40,00	33,38	40,00	40,00	40,00	34,49	40,00
40,00	40,00	40,00	40,00	40,00	40,00	40,00	29,96	40,00
40,00	40,00	40,00	34,09	40,00	40,00	40,00	31,13	40,00
40,00	40,00	40,00	33,26	40,00	40,00	40,00	31,99	40,00
40,00	35,32	40,00	33,77	40,00	40,00	40,00	28,94	40,00
40,00	40,00	40,00	34,28	40,00	40,00	40,00	28,74	40,00
40,00	40,00	40,00	40,00	40,00	40,00	40,00	30,91	40,00
40,00	40,00	40,00	40,00	40,00	40,00	40,00	34,15	40,00

hsa-miR-220b	hsa-miR-220c	hsa-miR-221	hsa-miR-222	hsa-miR-223	hsa-miR-224	hsa-miR-23a	hsa-miR-23b	hsa-miR-24	hsa-miR-25
40,00	40,00	27,58	23,24	19,03	33,26	36,49	40,00	21,50	29,28
40,00	40,00	28,97	25,44	21,49	40,00	40,00	40,00	24,97	31,48
40,00	40,00	25,83	22,22	17,02	34,42	40,00	40,00	21,43	28,14
40,00	40,00	23,49	20,80	15,00	30,52	32,44	40,00	19,34	26,03
40,00	40,00	24,17	21,47	16,94	30,72	33,70	40,00	21,39	27,04
40,00	40,00	24,68	23,02	17,21	30,83	32,23	40,00	22,10	27,61
40,00	40,00	26,39	23,60	19,27	32,23	33,78	40,00	23,65	28,36
40,00	40,00	24,89	21,61	16,86	29,43	30,36	40,00	21,43	24,49
40,00	40,00	21,97	19,15	14,21	32,16	27,62	40,00	19,28	22,90
40,00	40,00	22,23	19,81	14,38	28,08	27,67	40,00	19,12	22,74
40,00	40,00	24,29	22,05	17,13	30,08	30,53	40,00	21,49	25,18
40,00	40,00	25,40	22,73	17,60	34,49	33,09	40,00	22,63	26,95
40,00	40,00	23,67	21,62	16,13	28,84	30,41	40,00	20,63	24,67
40,00	40,00	25,10	22,16	17,78	30,78	33,47	40,00	22,14	25,41
40,00	40,00	25,78	23,52	18,18	30,14	33,34	40,00	22,72	27,64
40,00	40,00	25,15	23,59	17,86	29,96	32,07	40,00	22,66	26,91
40,00	40,00	25,40	23,28	18,00	30,43	33,35	40,00	22,86	27,51
40,00	40,00	28,55	26,01	20,54	40,00	40,00	40,00	25,34	29,26
40,00	40,00	26,04	23,25	17,83	31,94	33,17	40,00	23,33	28,10
40,00	40,00	27,52	25,03	20,08	40,00	34,50	40,00	25,77	27,84
40,00	40,00	26,18	23,85	18,02	40,00	34,43	40,00	24,02	28,20
40,00	40,00	24,63	23,03	17,38	30,81	37,57	40,00	21,42	25,85
40,00	40,00	25,03	23,58	17,71	29,92	34,15	40,00	21,90	24,96
40,00	40,00	23,82	21,79	16,34	27,43	32,63	40,00	20,24	24,75
40,00	40,00	22,95	22,19	16,18	28,86	34,29	40,00	20,00	24,63
40,00	40,00	25,57	24,12	18,47	32,17	36,10	40,00	22,29	26,01
40,00	40,00	23,55	22,15	16,33	27,67	36,74	40,00	20,56	24,09
40,00	40,00	23,30	22,32	16,09	27,92	36,95	40,00	20,08	24,90
40,00	40,00	26,02	24,07	18,77	40,00	36,45	40,00	22,36	27,94
40,00	40,00	25,46	22,60	16,74	30,23	40,00	40,00	20,54	26,46
40,00	40,00	23,07	21,14	15,35	26,93	33,97	40,00	19,37	23,98
40,00	40,00	23,49	21,32	15,62	29,87	32,67	38,40	19,05	24,83
40,00	40,00	23,32	21,00	14,96	28,77	34,13	40,00	19,20	24,97
40,00	40,00	23,87	21,94	15,87	30,11	35,77	40,00	20,06	24,96
40,00	40,00	25,99	24,21	18,30	30,94	36,89	40,00	22,25	26,19
40,00	40,00	24,36	23,11	16,90	31,20	35,19	40,00	20,85	24,92
40,00	40,00	25,44	22,76	16,54	27,95	38,37	40,00	20,52	25,83
40,00	40,00	25,12	23,05	17,74	28,42	37,59	40,00	22,12	25,29
40,00	40,00	23,57	21,65	15,96	28,93	33,96	37,55	19,80	24,67
40,00	40,00	24,06	21,62	15,96	29,94	39,51	40,00	20,04	25,07
40,00	40,00	25,68	22,99	17,94	34,58	40,00	40,00	21,95	24,94
40,00	40,00	27,10	24,18	19,12	33,41	40,00	40,00	22,84	27,13

hsa-miR-26a	hsa-miR-26b	hsa-miR-27a	hsa-miR-27b	hsa-miR-28	hsa-miR-28-3p	hsa-miR-296	hsa-miR-296-3p	hsa-miR-298	hsa-miR-299-3p
27,61	29,92	28,02	29,78	33,00	26,99	40,00	40,00	40,00	40,00
29,38	33,78	31,43	40,00	34,95	28,95	40,00	40,00	40,00	40,00
25,49	28,40	27,45	29,36	30,34	26,25	33,57	40,00	40,00	40,00
23,07	26,20	26,29	28,30	27,64	23,86	40,00	40,00	40,00	40,00
24,89	27,95	27,71	28,38	28,99	25,31	34,24	40,00	40,00	40,00
25,42	28,23	27,84	30,33	29,61	25,96	34,41	40,00	40,00	40,00
27,65	31,10	29,27	31,22	30,89	27,94	40,00	40,00	40,00	40,00
24,49	27,02	24,59	25,84	29,01	25,04	40,00	40,00	40,00	40,00
21,47	24,22	22,32	24,05	26,26	23,22	29,86	40,00	40,00	40,00
22,32	24,77	22,83	23,99	26,82	23,11	32,69	40,00	40,00	40,00
25,45	27,84	24,83	26,30	29,89	25,95	32,27	35,87	40,00	40,00
25,45	27,97	25,86	27,18	29,21	25,86	40,00	40,00	40,00	40,00
23,46	25,90	24,35	25,02	27,89	24,92	32,26	40,00	40,00	40,00
27,24	28,42	25,26	27,93	32,34	25,99	34,00	40,00	40,00	40,00
25,52	28,04	27,10	28,14	29,68	27,00	40,00	40,00	40,00	40,00
24,99	27,31	26,31	27,68	28,94	26,95	34,23	40,00	40,00	40,00
25,13	28,03	26,55	27,78	29,01	26,95	40,00	40,00	40,00	40,00
28,46	30,58	30,17	30,78	33,62	29,35	40,00	40,00	40,00	40,00
25,32	27,72	27,19	29,36	29,27	27,05	32,80	40,00	40,00	40,00
27,72	29,88	28,64	29,39	32,88	28,98	40,00	40,00	40,00	40,00
25,88	27,97	27,52	29,58	30,57	27,89	35,61	40,00	40,00	40,00
25,78	27,58	26,69	29,25	28,85	26,25	31,70	40,00	40,00	40,00
25,66	26,95	26,83	28,50	28,33	26,70	31,24	40,00	40,00	40,00
24,65	26,09	25,78	26,88	28,38	25,45	32,91	40,00	40,00	40,00
23,92	25,67	24,93	26,68	26,75	25,30	31,49	40,00	40,00	40,00
26,89	28,08	27,58	28,43	29,48	28,31	32,55	40,00	40,00	40,00
24,46	26,01	25,07	26,58	28,62	25,95	30,03	40,00	40,00	40,00
25,37	27,68	25,06	26,34	28,60	25,56	32,41	40,00	40,00	40,00
27,36	29,00	27,66	28,92	29,90	27,07	33,25	40,00	40,00	40,00
25,35	27,18	26,30	28,95	28,96	25,43	33,52	40,00	40,00	40,00
23,13	25,23	24,59	25,85	26,97	24,27	30,92	40,00	40,00	40,00
23,64	25,38	24,76	26,35	26,96	24,65	32,09	40,00	40,00	40,00
22,97	25,14	24,65	25,81	26,83	23,86	31,83	40,00	40,00	40,00
25,49	26,59	26,04	27,47	28,23	25,06	31,98	40,00	40,00	40,00
27,09	28,63	27,79	28,86	30,52	27,66	32,71	40,00	40,00	40,00
25,47	26,67	25,91	26,65	28,91	25,85	31,91	40,00	40,00	40,00
26,30	28,04	27,00	28,71	29,71	25,34	31,63	40,00	40,00	40,00
25,78	27,34	26,53	27,56	28,55	26,97	31,29	40,00	40,00	40,00
23,94	25,82	25,39	26,51	27,36	24,62	30,19	40,00	40,00	40,00
25,76	27,43	25,88	26,98	28,54	24,41	32,01	40,00	40,00	40,00
26,60	28,09	26,54	29,16	29,95	26,79	32,05	40,00	40,00	40,00
29,65	29,20	28,10	29,34	34,64	26,93	34,49	40,00	40,00	40,00

hsa-miR-299-5p	hsa-miR-29a	hsa-miR-29b	hsa-miR-29c	hsa-miR-301	hsa-miR-301b	hsa-miR-302a	hsa-miR-302b	hsa-miR-302c	hsa-miR-30b
40,00	29,67	40,00	34,42	33,96	40,00	40,00	40,00	40,00	26,67
40,00	32,23	40,00	37,28	34,88	40,00	40,00	40,00	40,00	28,95
40,00	27,58	40,00	33,20	29,41	40,00	40,00	40,00	40,00	24,90
40,00	26,80	40,00	31,10	27,54	32,54	40,00	40,00	40,00	22,47
40,00	29,03	40,00	33,77	28,60	32,65	40,00	40,00	40,00	24,38
40,00	27,92	40,00	32,77	29,69	40,00	40,00	40,00	40,00	24,93
40,00	29,95	40,00	34,05	32,80	40,00	40,00	40,00	40,00	27,43
40,00	26,81	34,39	31,94	29,31	33,61	40,00	40,00	40,00	24,79
40,00	24,37	31,31	28,43	26,69	28,79	40,00	40,00	40,00	21,89
40,00	24,88	40,00	29,37	27,35	29,33	40,00	40,00	40,00	22,58
40,00	27,82	32,96	31,29	30,36	33,97	40,00	40,00	40,00	25,32
40,00	27,58	40,00	32,06	30,34	33,78	40,00	40,00	40,00	25,39
40,00	26,09	33,04	31,35	29,07	31,65	40,00	40,00	40,00	23,69
40,00	28,21	40,00	32,01	31,10	31,31	40,00	40,00	40,00	27,60
40,00	27,86	40,00	32,84	32,23	32,55	40,00	40,00	40,00	25,33
40,00	28,77	32,99	32,83	29,21	40,00	40,00	40,00	40,00	25,14
40,00	27,71	40,00	31,87	30,59	40,00	40,00	40,00	40,00	25,64
40,00	30,60	40,00	35,99	32,06	40,00	40,00	40,00	40,00	27,73
40,00	28,07	34,02	32,81	29,17	32,82	40,00	40,00	40,00	25,33
40,00	30,06	40,00	36,89	32,45	40,00	40,00	40,00	40,00	28,36
40,00	28,94	32,80	33,22	30,38	40,00	40,00	40,00	40,00	25,85
40,00	27,90	40,00	32,79	29,96	31,19	40,00	40,00	40,00	25,59
40,00	28,00	40,00	31,98	28,89	32,26	40,00	40,00	40,00	24,91
40,00	26,60	40,00	32,09	28,18	32,34	40,00	40,00	40,00	24,04
40,00	25,86	32,15	30,08	27,86	30,29	40,00	40,00	40,00	22,99
40,00	28,83	40,00	33,11	30,69	33,81	40,00	40,00	40,00	25,95
40,00	26,36	40,00	30,36	28,17	30,99	40,00	40,00	40,00	23,57
40,00	26,35	40,00	31,30	29,26	31,24	40,00	40,00	40,00	25,01
40,00	29,91	40,00	33,65	30,30	32,90	40,00	40,00	40,00	26,50
40,00	26,96	34,75	32,23	29,96	31,44	40,00	40,00	40,00	24,48
40,00	25,75	31,31	30,78	27,37	31,75	40,00	40,00	40,00	22,75
40,00	25,20	34,35	29,73	28,61	32,42	40,00	40,00	40,00	23,46
40,00	24,88	32,59	29,72	27,16	31,15	40,00	40,00	40,00	22,41
40,00	26,26	40,00	30,59	28,91	31,82	40,00	40,00	40,00	24,99
40,00	28,66	40,00	33,63	30,83	40,00	36,11	40,00	40,00	25,80
40,00	27,76	34,96	32,41	28,45	33,78	40,00	40,00	40,00	25,60
40,00	27,82	40,00	32,50	29,32	34,37	40,00	40,00	40,00	25,32
40,00	28,45	37,22	32,80	28,99	33,82	40,00	40,00	40,00	24,80
40,00	26,40	33,27	31,53	28,15	32,60	40,00	40,00	40,00	23,22
40,00	27,48	40,00	32,27	28,96	32,66	40,00	40,00	40,00	24,65
40,00	27,93	40,00	32,19	30,41	35,85	40,00	40,00	40,00	25,85
40,00	29,66	40,00	34,64	31,43	39,26	40,00	40,00	40,00	28,23

hsa-miR-30c	hsa-miR-31	hsa-miR-32	hsa-miR-320	hsa-miR-323-3p	hsa-miR-324-3p	hsa-miR-324-5p	hsa-miR-325	hsa-miR-326	hsa-miR-328
27,00	40,00	40,00	21,25	31,73	34,39	31,03	40,00	40,00	26,45
28,70	40,00	40,00	24,25	34,05	40,00	40,00	40,00	40,00	31,04
24,58	40,00	40,00	22,06	30,52	30,96	30,78	40,00	40,00	25,47
22,69	40,00	31,55	19,23	30,25	29,97	30,02	40,00	40,00	23,71
24,53	40,00	40,00	20,32	29,62	30,82	30,62	40,00	40,00	25,46
24,93	40,00	34,32	21,30	31,19	30,96	31,88	40,00	40,00	25,93
26,77	40,00	40,00	22,66	37,49	32,77	33,30	40,00	40,00	27,66
25,18	40,00	40,00	20,49	29,92	28,36	29,15	40,00	40,00	25,06
21,95	32,21	31,61	18,72	25,95	26,62	26,16	40,00	35,37	22,57
23,08	35,23	33,57	18,83	29,54	26,96	26,63	40,00	34,59	22,76
25,78	40,00	40,00	20,20	30,91	28,84	30,21	40,00	40,00	24,96
25,58	40,00	40,00	21,49	30,40	30,34	29,88	40,00	36,01	25,74
24,04	40,00	34,05	19,97	29,68	28,81	28,29	40,00	40,00	24,14
28,21	34,76	34,69	20,00	31,02	29,57	30,87	40,00	38,68	25,24
25,97	40,00	40,00	22,44	35,42	30,33	30,77	40,00	40,00	26,66
25,68	40,00	33,90	21,95	31,74	29,93	30,92	40,00	40,00	26,31
25,97	40,00	40,00	22,02	34,46	30,21	30,24	40,00	40,00	26,32
27,74	40,00	40,00	22,95	32,95	40,00	40,00	40,00	40,00	27,85
25,37	40,00	40,00	21,14	32,42	30,40	31,11	40,00	40,00	25,88
27,77	40,00	40,00	22,84	31,98	32,69	40,00	40,00	40,00	27,72
25,94	40,00	40,00	23,03	31,37	30,80	32,58	40,00	36,15	26,84
24,93	40,00	40,00	22,22	30,66	29,75	29,67	40,00	40,00	24,23
24,64	40,00	40,00	23,46	30,61	29,35	29,64	40,00	40,00	25,27
23,63	40,00	40,00	20,91	29,46	28,29	29,47	40,00	37,71	23,13
22,95	34,43	40,00	22,02	30,41	27,37	28,31	40,00	34,11	23,57
25,37	40,00	40,00	23,89	32,68	30,56	40,00	40,00	36,44	26,21
23,25	33,12	40,00	21,50	29,58	28,05	30,38	40,00	33,78	23,60
24,18	40,00	40,00	21,13	27,95	27,06	28,68	40,00	33,68	23,25
25,52	40,00	40,00	22,51	29,12	29,91	31,90	40,00	34,61	25,10
23,87	33,86	40,00	22,08	29,84	28,63	29,89	40,00	40,00	24,99
21,97	37,51	40,00	20,70	28,34	26,73	28,08	40,00	33,46	22,91
23,06	31,15	36,56	21,10	30,12	26,11	27,93	40,00	32,61	23,89
21,71	37,59	34,45	21,06	27,74	27,31	27,91	40,00	34,27	23,31
24,54	32,54	40,00	21,31	28,43	27,97	28,94	40,00	35,13	23,44
25,62	40,00	40,00	23,18	31,79	30,29	30,90	40,00	40,00	25,15
24,87	34,51	40,00	21,12	28,63	28,80	31,85	40,00	37,33	23,41
24,79	33,31	40,00	20,92	30,32	28,98	30,88	40,00	34,73	23,65
24,63	36,39	40,00	22,62	30,66	29,53	30,08	40,00	36,98	24,85
22,64	38,83	40,00	20,45	29,99	27,60	28,97	40,00	33,94	23,11
24,20	34,34	40,00	20,49	27,98	27,81	30,02	40,00	40,00	22,93
25,27	34,94	40,00	22,76	30,37	28,98	30,37	40,00	35,08	24,70
27,15	36,01	40,00	22,60	30,31	30,35	40,00	40,00	36,97	26,16

hsa-miR-329	hsa-miR-330	hsa-miR-330-5p	hsa-miR-331	hsa-miR-331-5p	hsa-miR-335	hsa-miR-337-5p	hsa-miR-338-3p	hsa-miR-339-3p
40,00	37,51	40,00	28,54	40,00	29,37	40,00	40,00	31,57
40,00	40,00	40,00	40,00	40,00	32,03	40,00	40,00	36,77
40,00	33,62	40,00	27,08	40,00	28,30	40,00	32,80	28,60
33,78	32,75	40,00	25,66	40,00	26,45	40,00	31,03	28,36
37,30	33,45	40,00	27,74	40,00	27,75	40,00	40,00	28,40
40,00	34,47	40,00	28,26	40,00	28,91	40,00	33,16	28,49
40,00	40,00	40,00	32,40	40,00	29,91	40,00	40,00	30,98
38,64	32,93	40,00	27,18	40,00	27,33	28,43	31,23	31,33
31,52	30,06	40,00	24,25	40,00	24,57	26,23	29,86	24,84
31,22	30,15	40,00	24,96	31,69	25,14	29,10	30,38	27,63
40,00	33,95	40,00	28,31	40,00	27,18	29,62	32,43	31,10
33,97	38,43	40,00	27,97	40,00	28,06	31,95	40,00	28,74
35,40	31,66	40,00	26,60	40,00	26,66	30,04	32,58	26,56
40,00	34,25	40,00	29,76	40,00	26,95	31,63	31,85	27,81
40,00	34,87	40,00	28,50	40,00	29,23	32,13	40,00	31,85
40,00	33,35	40,00	27,76	40,00	28,64	40,00	40,00	28,97
40,00	34,88	40,00	28,10	40,00	29,03	31,63	32,26	33,25
40,00	35,97	40,00	31,37	40,00	30,53	40,00	40,00	37,39
37,97	34,04	40,00	27,92	40,00	28,58	40,00	35,91	27,99
40,00	35,71	40,00	30,24	40,00	32,58	40,00	40,00	30,99
40,00	35,81	40,00	28,93	40,00	30,15	40,00	40,00	28,98
40,00	32,36	40,00	26,65	40,00	28,34	31,31	40,00	28,83
40,00	32,48	40,00	26,78	40,00	28,45	40,00	35,37	30,36
36,30	32,22	40,00	25,22	40,00	26,67	32,98	35,83	28,55
34,94	31,61	40,00	24,31	36,15	26,33	32,10	40,00	28,47
40,00	34,18	40,00	28,60	34,13	28,87	40,00	40,00	31,05
40,00	31,94	40,00	25,05	35,77	26,81	32,54	40,00	28,72
34,61	31,47	40,00	25,53	36,45	26,53	31,80	33,67	29,05
40,00	33,48	40,00	27,66	40,00	28,00	35,22	40,00	29,96
37,94	32,88	40,00	25,50	40,00	27,40	31,88	40,00	28,16
33,62	31,83	40,00	24,06	38,68	24,87	31,31	36,04	27,59
38,75	32,20	40,00	24,65	37,92	25,98	34,33	40,00	26,87
35,40	31,56	40,00	23,38	36,55	25,37	30,36	32,46	26,92
35,18	31,15	40,00	25,49	38,62	26,49	30,76	40,00	27,55
40,00	33,15	40,00	27,79	40,00	28,43	40,00	39,19	29,81
37,65	33,11	40,00	26,68	40,00	26,26	30,89	34,28	28,95
40,00	31,33	35,40	26,00	40,00	27,15	40,00	35,65	27,74
36,62	33,92	40,00	26,30	40,00	27,95	33,75	35,22	30,20
40,00	31,41	40,00	24,21	38,03	26,02	32,19	32,08	27,27
34,62	31,79	40,00	25,95	39,85	26,26	33,59	34,50	27,34
37,78	33,71	40,00	26,73	34,57	27,59	31,97	33,97	29,55
35,27	34,68	40,00	29,15	40,00	28,72	35,48	40,00	30,11

hsa-miR-339-5p	hsa-miR-33b	hsa-miR-340	hsa-miR-342-3p	hsa-miR-342-5p	hsa-miR-345	hsa-miR-346	hsa-miR-34a	hsa-miR-34c	hsa-miR-361
40,00	40,00	30,79	26,37	40,00	29,36	40,00	40,00	40,00	33,54
40,00	40,00	40,00	28,84	40,00	33,95	40,00	40,00	40,00	40,00
40,00	40,00	29,53	25,31	40,00	28,62	40,00	40,00	40,00	30,14
31,83	40,00	27,72	23,39	40,00	26,95	40,00	40,00	40,00	29,28
40,00	40,00	28,66	24,26	40,00	28,69	40,00	40,00	40,00	31,30
40,00	40,00	29,95	25,56	40,00	29,58	40,00	31,36	40,00	32,02
40,00	40,00	30,29	26,33	40,00	29,76	40,00	40,00	40,00	34,04
40,00	40,00	28,65	25,40	40,00	27,06	40,00	31,16	40,00	28,40
27,84	40,00	25,70	22,67	36,11	25,54	40,00	26,29	40,00	26,32
28,60	40,00	26,36	22,66	40,00	25,44	40,00	30,34	40,00	26,44
40,00	35,61	28,70	25,02	40,00	28,08	40,00	33,49	40,00	27,97
40,00	40,00	28,42	25,32	40,00	28,13	40,00	32,21	40,00	32,36
29,81	40,00	28,24	24,46	35,34	27,05	40,00	34,50	40,00	27,72
40,00	40,00	27,77	25,00	40,00	28,09	40,00	31,16	40,00	30,04
40,00	40,00	29,42	27,47	40,00	28,55	40,00	33,71	40,00	30,04
40,00	40,00	30,34	25,73	40,00	28,82	40,00	31,99	40,00	31,41
40,00	40,00	31,17	27,21	40,00	28,30	40,00	40,00	40,00	29,85
40,00	40,00	33,08	26,84	40,00	29,89	40,00	40,00	40,00	31,98
40,00	40,00	29,25	25,55	40,00	29,37	40,00	40,00	40,00	30,05
40,00	40,00	32,37	26,17	40,00	29,62	40,00	40,00	40,00	34,06
36,49	40,00	29,57	27,29	40,00	30,34	40,00	33,37	40,00	30,50
34,59	40,00	29,10	24,82	40,00	28,33	40,00	31,03	40,00	30,05
32,43	40,00	29,51	24,99	40,00	28,75	40,00	29,86	40,00	31,71
31,48	32,48	28,87	24,19	35,97	27,60	40,00	36,14	40,00	28,48
30,00	40,00	27,62	24,82	40,00	27,51	40,00	31,98	39,25	28,59
38,05	40,00	30,26	25,85	39,69	29,75	40,00	30,56	40,00	30,41
31,44	34,61	27,94	23,74	40,00	27,48	40,00	28,87	40,00	28,84
31,74	40,00	28,43	25,22	40,00	27,81	40,00	31,34	40,00	28,03
33,37	40,00	30,57	25,77	40,00	28,63	40,00	40,00	40,00	34,38
30,71	40,00	28,75	24,00	40,00	28,00	40,00	31,69	40,00	29,62
28,71	40,00	26,59	24,17	38,26	26,00	40,00	40,00	40,00	27,40
27,90	40,00	27,74	25,47	40,00	27,02	40,00	29,57	40,00	28,27
28,25	40,00	26,70	23,71	38,63	26,77	40,00	31,86	40,00	28,17
33,29	40,00	28,29	23,91	36,73	26,51	40,00	30,48	36,59	29,25
32,47	40,00	29,65	25,32	40,00	29,14	40,00	40,00	40,00	30,74
36,09	40,00	29,31	23,61	36,39	28,76	40,00	32,95	40,00	29,75
32,66	40,00	28,86	24,08	37,80	27,43	40,00	40,00	40,00	30,24
32,29	40,00	29,90	25,51	40,00	28,53	40,00	31,69	40,00	30,07
29,68	40,00	27,45	23,59	39,37	26,82	40,00	33,15	40,00	28,11
31,08	40,00	28,27	23,43	36,54	27,34	40,00	40,00	40,00	28,64
37,17	40,00	30,35	27,84	39,93	29,01	40,00	32,78	40,00	30,79
40,00	40,00	31,66	25,66	40,00	29,67	40,00	31,22	40,00	31,73

hsa-miR-362	hsa-miR-362-3p	hsa-miR-363	hsa-miR-365	hsa-miR-367	hsa-miR-369-3p	hsa-miR-369-5p	hsa-miR-370	hsa-miR-371-3p
40,00	40,00	40,00	40,00	40,00	40,00	40,00	40,00	40,00
40,00	40,00	40,00	40,00	40,00	40,00	40,00	34,49	40,00
40,00	32,04	40,00	31,87	40,00	40,00	40,00	32,14	40,00
32,22	32,61	40,00	40,00	40,00	40,00	40,00	31,20	40,00
40,00	40,00	40,00	40,00	40,00	40,00	40,00	29,62	40,00
40,00	40,00	33,37	34,24	40,00	40,00	40,00	33,31	40,00
40,00	40,00	40,00	34,74	40,00	40,00	40,00	34,93	40,00
40,00	40,00	33,95	40,00	40,00	40,00	40,00	29,24	40,00
31,49	33,96	30,91	31,54	40,00	32,76	32,05	24,86	40,00
40,00	40,00	29,77	40,00	40,00	40,00	32,71	29,02	40,00
33,37	34,37	30,96	40,00	40,00	40,00	40,00	33,38	40,00
35,43	40,00	40,00	31,93	40,00	34,71	40,00	29,51	40,00
32,14	40,00	34,51	31,72	40,00	40,00	40,00	30,10	40,00
40,00	40,00	31,55	32,70	40,00	40,00	40,00	35,94	40,00
40,00	40,00	33,04	40,00	40,00	40,00	40,00	31,29	40,00
40,00	33,80	40,00	40,00	40,00	40,00	40,00	40,00	40,00
34,94	32,88	33,22	40,00	40,00	33,37	40,00	31,92	40,00
40,00	40,00	40,00	40,00	40,00	40,00	40,00	35,71	40,00
40,00	40,00	32,41	33,83	40,00	40,00	40,00	31,55	40,00
40,00	40,00	34,04	34,02	40,00	40,00	40,00	33,20	40,00
40,00	40,00	34,60	34,26	40,00	40,00	40,00	30,25	40,00
40,00	40,00	32,10	31,03	40,00	40,00	40,00	37,22	40,00
33,97	40,00	33,23	31,50	40,00	40,00	40,00	36,25	40,00
33,80	34,83	37,65	31,24	40,00	34,72	40,00	38,47	40,00
35,54	40,00	32,13	30,39	40,00	40,00	36,61	37,71	40,00
40,00	40,00	35,66	34,10	40,00	40,00	40,00	40,00	40,00
40,00	40,00	35,44	29,55	40,00	40,00	40,00	33,66	40,00
34,18	33,96	31,65	32,62	40,00	40,00	39,82	32,90	40,00
40,00	33,34	40,00	36,46	40,00	40,00	40,00	36,93	40,00
40,00	40,00	34,29	33,45	40,00	40,00	38,80	29,13	40,00
31,68	32,23	34,51	33,12	40,00	40,00	39,72	28,36	40,00
40,00	31,87	33,04	34,07	40,00	40,00	40,00	31,01	40,00
32,33	31,84	39,18	30,93	40,00	33,07	38,67	27,65	40,00
35,76	40,00	33,15	31,43	40,00	40,00	38,23	29,48	40,00
40,00	40,00	39,30	34,89	40,00	40,00	34,85	40,00	40,00
40,00	40,00	40,00	33,33	40,00	40,00	40,00	36,99	40,00
40,00	40,00	40,00	31,68	40,00	40,00	40,00	40,00	40,00
40,00	40,00	38,30	32,23	40,00	40,00	40,00	40,00	40,00
34,73	35,17	34,03	32,23	40,00	40,00	40,00	32,93	40,00
33,88	40,00	31,79	33,81	40,00	40,00	35,38	30,53	40,00
40,00	34,13	33,44	32,74	40,00	34,82	34,45	39,19	40,00
40,00	40,00	40,00	34,25	40,00	40,00	40,00	40,00	40,00

hsa-miR-372	hsa-miR-373	hsa-miR-374	hsa-miR-375	hsa-miR-376a	hsa-miR-376b	hsa-miR-376c	hsa-miR-377	hsa-miR-380-3p	hsa-miR-381
40,00	40,00	29,21	40,00	28,77	40,00	28,65	40,00	40,00	40,00
40,00	40,00	32,55	33,27	33,07	40,00	32,68	40,00	40,00	40,00
40,00	40,00	27,96	30,02	28,36	40,00	28,38	40,00	40,00	27,43
40,00	40,00	25,77	31,29	28,61	40,00	28,21	33,20	40,00	40,00
40,00	40,00	28,04	32,99	28,72	40,00	27,58	40,00	40,00	28,51
40,00	40,00	28,45	40,00	30,43	40,00	30,20	40,00	40,00	27,54
40,00	40,00	30,23	30,98	31,79	40,00	31,27	40,00	40,00	25,72
40,00	40,00	27,98	28,92	27,22	40,00	26,50	40,00	40,00	40,00
40,00	40,00	24,79	28,19	24,03	35,52	22,94	32,22	40,00	24,95
40,00	40,00	25,77	30,57	27,04	40,00	26,78	40,00	40,00	40,00
40,00	40,00	28,10	28,97	27,10	40,00	26,17	40,00	40,00	40,00
40,00	40,00	28,24	30,97	28,72	40,00	27,47	40,00	40,00	26,17
40,00	40,00	26,40	29,24	27,45	40,00	26,65	40,00	40,00	25,53
40,00	40,00	30,10	40,00	29,50	40,00	28,51	40,00	40,00	25,56
40,00	40,00	28,72	32,83	31,61	40,00	30,19	40,00	40,00	40,00
40,00	40,00	27,67	30,89	32,56	40,00	30,68	40,00	40,00	24,62
40,00	40,00	27,98	40,00	29,51	40,00	29,44	40,00	40,00	33,48
40,00	40,00	30,70	40,00	32,17	40,00	32,23	40,00	40,00	40,00
40,00	40,00	28,21	31,03	30,28	40,00	29,01	40,00	40,00	26,62
40,00	40,00	30,19	29,10	31,39	40,00	31,00	40,00	40,00	24,34
40,00	40,00	28,33	31,93	31,37	40,00	28,85	40,00	40,00	24,76
39,39	40,00	29,13	27,70	29,52	40,00	30,08	40,00	40,00	22,47
38,73	40,00	27,78	27,66	32,27	40,00	31,02	40,00	40,00	23,66
40,00	33,10	27,39	29,71	28,44	40,00	28,32	40,00	40,00	22,89
37,54	40,00	26,47	27,44	28,91	40,00	28,71	40,00	40,00	22,01
40,00	40,00	29,80	28,32	30,14	40,00	30,53	40,00	40,00	22,52
37,99	33,86	26,80	25,95	28,25	40,00	28,72	40,00	40,00	21,01
37,81	33,33	27,99	28,61	27,00	40,00	26,52	40,00	40,00	22,09
39,00	40,00	30,78	29,51	28,95	40,00	28,75	40,00	40,00	22,09
40,00	40,00	27,72	28,71	27,76	40,00	26,92	40,00	40,00	24,69
38,22	40,00	25,78	28,83	26,70	40,00	26,05	40,00	40,00	21,83
38,88	32,34	25,62	30,02	27,06	40,00	26,59	40,00	40,00	23,35
34,00	33,99	25,16	28,06	25,85	40,00	24,93	40,00	40,00	22,58
40,00	40,00	27,84	28,45	26,69	40,00	25,98	34,05	38,41	23,59
40,00	40,00	29,58	27,29	29,37	40,00	30,22	40,00	40,00	23,17
39,58	33,91	28,98	28,42	26,41	40,00	26,05	40,00	40,00	22,20
40,00	40,00	29,05	28,42	29,17	40,00	28,04	40,00	40,00	24,32
38,90	33,82	28,42	28,28	30,26	40,00	30,28	40,00	40,00	22,46
40,00	32,74	26,82	27,92	29,41	40,00	29,32	40,00	40,00	23,93
40,00	40,00	28,06	29,41	26,58	40,00	25,41	40,00	40,00	23,51
31,52	40,00	30,25	27,98	28,68	40,00	27,63	40,00	40,00	21,41
40,00	40,00	31,49	26,98	29,24	40,00	28,65	40,00	40,00	23,13

hsa-miR-382	hsa-miR-383	hsa-miR-384	hsa-miR-409-5p	hsa-miR-410	hsa-miR-411	hsa-miR-412	hsa-miR-422a	hsa-miR-423-5p	hsa-miR-424
31,42	40,00	40,00	40,00	32,76	32,22	40,00	40,00	31,90	40,00
40,00	40,00	40,00	40,00	34,69	40,00	40,00	40,00	33,03	40,00
31,26	40,00	40,00	40,00	32,46	33,37	40,00	40,00	29,54	40,00
29,29	40,00	40,00	40,00	31,45	29,79	40,00	40,00	28,31	40,00
28,30	40,00	40,00	34,67	30,84	32,16	40,00	40,00	29,17	40,00
40,00	40,00	40,00	40,00	34,45	33,07	40,00	40,00	30,10	40,00
40,00	40,00	40,00	40,00	35,36	40,00	40,00	40,00	31,02	40,00
27,01	40,00	40,00	38,03	29,45	29,58	40,00	38,96	28,08	33,25
23,56	40,00	40,00	32,61	25,47	26,08	40,00	39,20	25,78	30,44
27,10	40,00	40,00	40,00	29,86	28,80	40,00	37,63	26,31	31,67
29,14	40,00	40,00	40,00	30,01	32,05	40,00	40,00	28,85	33,56
31,61	40,00	40,00	35,63	30,54	32,78	40,00	40,00	29,69	40,00
27,66	40,00	40,00	40,00	29,27	32,12	40,00	40,00	27,14	32,14
31,37	40,00	40,00	40,00	31,52	37,94	40,00	33,26	27,88	40,00
34,52	40,00	40,00	40,00	31,43	31,56	40,00	40,00	29,27	40,00
40,00	40,00	40,00	40,00	32,74	36,83	40,00	40,00	28,73	33,26
40,00	40,00	40,00	40,00	33,09	31,15	40,00	40,00	29,57	40,00
40,00	40,00	40,00	40,00	34,33	32,46	40,00	40,00	31,20	40,00
40,00	40,00	40,00	40,00	31,96	35,23	40,00	40,00	29,26	33,08
33,67	40,00	40,00	40,00	33,92	40,00	40,00	40,00	30,07	40,00
35,25	40,00	40,00	40,00	32,87	37,05	40,00	40,00	29,72	32,23
29,66	40,00	40,00	40,00	31,93	40,00	40,00	40,00	28,93	40,00
31,15	40,00	40,00	40,00	34,49	40,00	40,00	33,89	28,89	40,00
27,86	40,00	40,00	40,00	30,94	36,76	40,00	40,00	27,10	38,21
29,00	40,00	40,00	38,94	32,06	39,37	40,00	40,00	27,30	33,23
32,71	38,02	40,00	40,00	32,21	40,00	40,00	40,00	30,34	40,00
28,95	40,00	40,00	40,00	31,55	40,00	40,00	40,00	27,59	40,00
25,22	40,00	40,00	40,00	29,73	37,31	40,00	40,00	26,99	40,00
27,56	40,00	40,00	40,00	32,67	40,00	40,00	40,00	28,11	40,00
28,52	40,00	40,00	40,00	30,67	38,06	40,00	40,00	29,94	40,00
25,82	40,00	40,00	40,00	28,81	35,58	40,00	40,00	26,43	33,49
25,80	40,00	40,00	40,00	31,14	35,17	40,00	40,00	27,19	40,00
25,67	40,00	40,00	40,00	28,33	31,77	40,00	33,17	27,57	33,18
26,06	40,00	40,00	35,99	28,92	35,07	40,00	40,00	28,37	32,61
27,66	40,00	40,00	40,00	31,96	38,11	40,00	37,95	30,08	40,00
25,55	40,00	40,00	40,00	29,59	38,07	40,00	32,82	27,15	37,34
28,86	40,00	40,00	40,00	31,47	40,00	40,00	40,00	28,16	40,00
30,69	40,00	40,00	40,00	33,05	39,18	40,00	36,68	28,02	33,38
29,59	40,00	40,00	40,00	32,43	39,14	40,00	31,99	26,64	33,43
25,94	40,00	40,00	40,00	29,70	35,77	40,00	40,00	27,23	40,00
28,21	40,00	40,00	40,00	30,49	32,19	40,00	36,84	28,60	34,13
30,31	40,00	40,00	40,00	33,43	40,00	40,00	40,00	29,60	40,00

hsa-miR-425-5p	hsa-miR-429	hsa-miR-431	hsa-miR-433	hsa-miR-448	hsa-miR-449	hsa-miR-449b	hsa-miR-450a	hsa-miR-450b-3p
29,56	40,00	40,00	31,90	40,00	40,00	40,00	40,00	40,00
32,45	40,00	40,00	30,89	40,00	40,00	40,00	40,00	40,00
40,00	40,00	40,00	31,44	40,00	40,00	40,00	40,00	40,00
27,49	34,25	40,00	29,91	40,00	40,00	40,00	32,51	40,00
31,12	34,38	36,38	30,10	40,00	40,00	40,00	40,00	40,00
40,00	40,00	40,00	32,94	40,00	40,00	40,00	40,00	40,00
40,00	40,00	40,00	33,12	40,00	40,00	40,00	40,00	40,00
28,22	40,00	40,00	28,90	40,00	40,00	40,00	40,00	40,00
26,86	40,00	27,22	25,50	40,00	34,36	36,70	31,89	40,00
26,23	40,00	40,00	28,73	40,00	31,84	40,00	40,00	40,00
28,70	40,00	40,00	30,23	40,00	40,00	40,00	40,00	40,00
40,00	40,00	40,00	30,47	40,00	40,00	40,00	40,00	40,00
40,00	40,00	40,00	28,84	40,00	40,00	40,00	40,00	40,00
40,00	40,00	40,00	30,70	40,00	40,00	40,00	40,00	40,00
30,23	40,00	40,00	31,55	40,00	40,00	40,00	40,00	40,00
31,73	40,00	40,00	31,96	40,00	40,00	40,00	40,00	40,00
30,05	40,00	40,00	31,76	40,00	40,00	40,00	40,00	40,00
29,96	40,00	40,00	31,53	40,00	40,00	40,00	40,00	40,00
40,00	40,00	40,00	33,16	40,00	40,00	40,00	32,19	40,00
34,26	40,00	40,00	33,66	40,00	40,00	40,00	40,00	40,00
35,61	40,00	39,78	31,26	40,00	40,00	40,00	40,00	40,00
33,33	33,21	40,00	33,36	40,00	40,00	40,00	40,00	40,00
33,99	40,00	40,00	36,13	40,00	40,00	40,00	40,00	40,00
30,47	32,58	38,62	31,10	40,00	40,00	40,00	40,00	40,00
29,91	40,00	40,00	40,00	36,01	40,00	40,00	40,00	40,00
33,69	40,00	40,00	34,17	40,00	40,00	40,00	40,00	40,00
29,45	40,00	40,00	34,45	40,00	40,00	34,17	40,00	40,00
29,65	40,00	32,38	31,11	40,00	40,00	40,00	40,00	40,00
34,86	40,00	39,06	31,74	40,00	40,00	40,00	40,00	40,00
31,92	40,00	36,69	33,52	40,00	40,00	40,00	40,00	40,00
26,61	40,00	35,10	29,37	40,00	40,00	37,01	40,00	40,00
29,97	35,74	35,69	36,25	36,04	40,00	40,00	40,00	40,00
26,64	35,87	31,61	28,59	40,00	37,83	34,43	40,00	40,00
29,34	40,00	32,16	29,69	40,00	40,00	40,00	40,00	40,00
33,18	40,00	40,00	36,32	40,00	40,00	40,00	40,00	40,00
30,54	35,00	34,72	27,87	40,00	38,84	40,00	40,00	40,00
29,86	40,00	40,00	32,60	40,00	36,68	40,00	40,00	40,00
34,54	35,00	40,00	33,46	40,00	40,00	40,00	40,00	40,00
28,57	40,00	39,66	31,46	40,00	40,00	40,00	40,00	40,00
27,57	40,00	33,06	28,23	40,00	40,00	40,00	40,00	40,00
25,21	40,00	37,72	40,00	31,72	40,00	40,00	35,22	40,00
34,88	40,00	40,00	30,56	40,00	40,00	40,00	40,00	40,00

hsa-miR-450b-5p	hsa-miR-452	hsa-miR-453	hsa-miR-454	hsa-miR-455	hsa-miR-455-3p	hsa-miR-483-5p	hsa-miR-484	hsa-miR-485-3p
40,00	40,00	40,00	28,96	40,00	40,00	26,99	21,37	31,22
40,00	40,00	40,00	30,76	40,00	40,00	25,95	26,16	31,11
40,00	34,74	40,00	26,94	40,00	40,00	27,37	20,92	28,77
40,00	33,56	40,00	24,41	40,00	40,00	29,42	19,93	28,95
40,00	32,26	40,00	26,21	40,00	40,00	27,40	21,44	27,99
40,00	40,00	40,00	27,12	40,00	40,00	28,48	22,61	30,84
40,00	40,00	40,00	30,58	40,00	40,00	28,53	23,66	40,00
40,00	32,98	40,00	28,38	40,00	40,00	27,20	21,77	29,98
40,00	32,28	40,00	25,17	40,00	40,00	27,14	19,17	25,53
33,85	31,20	40,00	26,13	40,00	40,00	27,97	19,53	28,77
40,00	36,42	40,00	29,15	40,00	40,00	28,82	21,21	31,04
40,00	34,86	40,00	29,06	40,00	40,00	27,71	21,72	31,45
40,00	30,99	40,00	26,58	40,00	40,00	27,69	21,11	28,75
40,00	33,87	40,00	30,57	40,00	40,00	26,65	21,87	33,32
40,00	34,50	40,00	29,34	40,00	40,00	30,07	22,71	30,55
40,00	34,79	40,00	27,34	40,00	40,00	28,65	23,57	40,00
40,00	40,00	40,00	28,26	40,00	40,00	30,95	22,66	33,09
40,00	40,00	40,00	30,48	40,00	40,00	29,31	24,97	33,50
40,00	40,00	40,00	27,85	40,00	40,00	27,64	22,31	30,50
40,00	40,00	40,00	31,35	40,00	40,00	27,87	24,20	40,00
40,00	31,92	40,00	28,92	40,00	40,00	28,52	23,00	33,07
40,00	32,25	40,00	29,95	40,00	40,00	28,21	21,44	30,68
40,00	40,00	40,00	28,30	40,00	40,00	29,86	22,34	30,70
40,00	31,20	40,00	28,08	35,11	40,00	28,06	20,45	29,81
40,00	30,85	40,00	27,75	40,00	40,00	28,99	20,82	40,00
40,00	35,32	40,00	29,23	40,00	40,00	30,50	23,18	32,26
40,00	31,53	40,00	27,82	40,00	40,00	27,38	20,61	31,86
40,00	32,27	40,00	29,19	40,00	40,00	29,08	21,40	31,71
40,00	40,00	40,00	29,67	40,00	40,00	28,34	22,48	31,70
40,00	31,40	40,00	28,50	40,00	40,00	27,18	20,70	31,64
40,00	30,16	40,00	26,79	34,48	40,00	27,90	19,57	30,51
40,00	33,85	40,00	26,54	40,00	40,00	27,84	21,07	32,18
32,63	30,91	38,02	26,27	40,00	40,00	27,19	19,32	28,45
40,00	35,02	40,00	28,79	40,00	40,00	28,01	20,19	29,88
40,00	34,94	40,00	31,01	40,00	40,00	29,38	22,32	29,64
40,00	34,33	40,00	30,68	40,00	40,00	28,61	21,33	28,48
40,00	31,04	40,00	29,05	40,00	40,00	28,57	20,20	31,82
40,00	31,86	40,00	29,76	40,00	40,00	28,20	21,87	31,66
40,00	32,50	40,00	27,44	40,00	40,00	27,07	19,94	33,09
40,00	32,95	40,00	28,86	40,00	40,00	27,46	19,33	28,79
40,00	40,00	40,00	30,73	40,00	40,00	29,45	21,92	32,38
40,00	34,80	40,00	31,52	40,00	40,00	27,58	22,03	33,10

hsa-miR-485-5p	hsa-miR-486	hsa-miR-486-3p	hsa-miR-487a	hsa-miR-487b	hsa-miR-488	hsa-miR-489	hsa-miR-490	hsa-miR-491-3p
40,00	27,91	32,81	40,00	32,08	40,00	40,00	40,00	40,00
40,00	29,12	34,86	40,00	33,75	40,00	40,00	40,00	40,00
40,00	28,54	35,64	40,00	30,06	40,00	40,00	40,00	40,00
40,00	26,50	31,64	40,00	29,44	40,00	40,00	40,00	40,00
40,00	27,14	32,58	40,00	30,26	40,00	40,00	40,00	40,00
40,00	26,82	31,91	40,00	31,76	40,00	40,00	40,00	40,00
40,00	27,92	35,62	40,00	40,00	40,00	40,00	40,00	40,00
40,00	26,42	33,30	33,00	31,14	40,00	33,27	40,00	40,00
40,00	24,20	31,08	34,33	26,40	40,00	40,00	32,53	40,00
40,00	24,04	30,02	33,80	30,39	40,00	40,00	40,00	40,00
40,00	26,21	40,00	34,51	31,42	40,00	40,00	40,00	40,00
40,00	27,78	40,00	40,00	32,45	40,00	40,00	40,00	40,00
40,00	26,17	40,00	40,00	31,57	40,00	40,00	40,00	40,00
40,00	25,89	33,20	40,00	33,33	40,00	40,00	40,00	40,00
40,00	28,84	40,00	36,09	40,00	40,00	40,00	40,00	40,00
40,00	27,56	40,00	40,00	32,80	40,00	40,00	40,00	40,00
40,00	28,25	35,85	40,00	40,00	40,00	40,00	40,00	40,00
40,00	27,81	40,00	40,00	40,00	40,00	40,00	40,00	40,00
40,00	27,60	35,70	40,00	35,15	40,00	40,00	40,00	40,00
40,00	27,06	37,93	40,00	40,00	40,00	40,00	40,00	40,00
40,00	28,61	40,00	33,59	34,03	40,00	40,00	40,00	40,00
40,00	26,16	31,57	40,00	32,69	40,00	40,00	40,00	40,00
40,00	25,41	30,28	40,00	40,00	40,00	40,00	40,00	40,00
40,00	25,92	30,49	34,64	31,79	40,00	40,00	40,00	40,00
40,00	26,73	31,59	38,30	31,80	40,00	33,21	40,00	40,00
40,00	26,87	33,94	40,00	33,62	40,00	40,00	40,00	40,00
40,00	24,67	29,43	40,00	31,63	40,00	40,00	40,00	40,00
40,00	26,95	31,63	37,62	30,88	40,00	40,00	40,00	40,00
40,00	28,86	40,00	40,00	31,80	40,00	40,00	40,00	40,00
40,00	27,75	35,35	40,00	30,62	40,00	40,00	40,00	40,00
40,00	26,02	30,51	38,60	29,56	40,00	34,14	40,00	40,00
40,00	28,09	31,73	40,00	29,57	40,00	40,00	40,00	40,00
38,56	27,03	32,57	40,00	28,46	40,00	40,00	40,00	40,00
40,00	26,03	30,83	40,00	29,59	40,00	40,00	40,00	40,00
40,00	26,65	33,56	40,00	33,00	40,00	40,00	40,00	40,00
40,00	26,83	34,00	34,68	29,97	40,00	36,03	40,00	40,00
40,00	26,62	32,08	37,87	31,80	40,00	40,00	40,00	40,00
40,00	26,44	30,50	40,00	32,85	40,00	40,00	40,00	40,00
40,00	25,78	31,88	40,00	32,87	40,00	40,00	40,00	40,00
40,00	26,46	31,32	34,62	30,31	40,00	40,00	40,00	40,00
40,00	26,96	30,48	40,00	31,51	40,00	40,00	40,00	40,00
40,00	26,85	31,67	38,12	32,93	40,00	33,51	40,00	40,00

hsa-miR-492	hsa-miR-493	hsa-miR-494	hsa-miR-499-3p	hsa-miR-500	hsa-miR-501	hsa-miR-501-3p	hsa-miR-502	hsa-miR-502-3p	hsa-miR-503
40,00	33,38	32,51	40,00	40,00	40,00	40,00	40,00	34,64	40,00
40,00	40,00	40,00	40,00	40,00	40,00	40,00	40,00	40,00	40,00
40,00	40,00	31,72	40,00	40,00	40,00	40,00	40,00	40,00	40,00
40,00	31,95	31,24	40,00	40,00	32,34	40,00	40,00	32,98	40,00
40,00	32,57	31,05	40,00	40,00	40,00	40,00	40,00	40,00	40,00
40,00	40,00	40,00	40,00	40,00	40,00	40,00	40,00	40,00	40,00
40,00	40,00	33,45	40,00	40,00	40,00	40,00	40,00	40,00	40,00
40,00	33,80	29,09	40,00	40,00	40,00	40,00	40,00	32,59	40,00
40,00	27,81	25,18	40,00	31,87	40,00	35,47	37,56	31,76	40,00
40,00	31,67	27,98	40,00	31,05	33,16	40,00	40,00	31,57	40,00
40,00	30,97	31,47	40,00	40,00	40,00	40,00	40,00	33,22	40,00
40,00	33,12	30,49	40,00	40,00	40,00	40,00	40,00	40,00	40,00
40,00	30,36	29,31	40,00	40,00	40,00	40,00	40,00	40,00	40,00
40,00	32,90	32,09	40,00	40,00	40,00	40,00	40,00	40,00	40,00
40,00	40,00	33,50	40,00	40,00	40,00	40,00	40,00	40,00	40,00
40,00	40,00	30,80	40,00	40,00	40,00	40,00	40,00	33,19	40,00
40,00	33,88	32,62	40,00	35,60	40,00	34,92	40,00	33,72	40,00
40,00	40,00	33,49	40,00	40,00	40,00	40,00	40,00	34,80	40,00
40,00	40,00	31,11	40,00	40,00	40,00	40,00	40,00	40,00	40,00
40,00	34,19	40,00	40,00	40,00	40,00	40,00	37,16	40,00	40,00
40,00	34,46	33,28	40,00	40,00	40,00	40,00	40,00	34,58	40,00
40,00	40,00	31,56	40,00	40,00	40,00	40,00	40,00	40,00	40,00
40,00	40,00	33,08	40,00	40,00	40,00	40,00	40,00	34,97	40,00
40,00	40,00	30,58	40,00	40,00	40,00	35,13	40,00	33,27	40,00
40,00	40,00	30,28	40,00	31,75	40,00	40,00	40,00	34,18	40,00
40,00	40,00	31,71	40,00	40,00	40,00	40,00	40,00	33,41	40,00
40,00	34,47	31,35	40,00	40,00	40,00	36,57	40,00	33,16	40,00
40,00	33,23	29,56	40,00	35,88	40,00	35,92	40,00	32,10	40,00
40,00	40,00	30,87	40,00	40,00	40,00	40,00	40,00	34,39	40,00
40,00	32,62	30,63	40,00	40,00	40,00	36,66	34,42	33,34	40,00
40,00	34,65	29,05	40,00	38,76	40,00	40,00	33,44	32,16	40,00
40,00	34,00	29,90	40,00	34,27	40,00	35,40	37,40	33,55	40,00
40,00	31,21	27,97	40,00	34,26	40,00	40,00	40,00	32,40	40,00
40,00	33,18	29,01	40,00	40,00	40,00	40,00	40,00	32,67	40,00
40,00	40,00	32,46	40,00	40,00	40,00	40,00	40,00	36,36	40,00
40,00	33,69	29,42	40,00	40,00	40,00	40,00	40,00	34,76	40,00
40,00	34,27	33,35	40,00	36,41	40,00	40,00	40,00	40,00	40,00
40,00	40,00	31,21	40,00	40,00	40,00	38,73	40,00	34,73	40,00
40,00	33,05	32,56	40,00	37,75	40,00	40,00	40,00	32,70	40,00
40,00	34,74	29,14	40,00	40,00	40,00	40,00	40,00	40,00	40,00
38,99	40,00	30,21	40,00	40,00	40,00	40,00	40,00	35,99	40,00
40,00	40,00	36,02	40,00	40,00	40,00	40,00	40,00	38,66	40,00

hsa-miR-504	hsa-miR-505	hsa-miR-506	hsa-miR-507	hsa-miR-508	hsa-miR-508-5p	hsa-miR-509-3-5p	hsa-miR-509-5p	hsa-miR-510
40,00	40,00	40,00	40,00	40,00	40,00	40,00	40,00	40,00
40,00	40,00	40,00	40,00	40,00	40,00	40,00	40,00	40,00
40,00	33,54	40,00	40,00	40,00	40,00	40,00	40,00	40,00
40,00	40,00	40,00	40,00	40,00	40,00	40,00	40,00	40,00
40,00	32,95	40,00	40,00	40,00	40,00	40,00	40,00	40,00
40,00	40,00	40,00	40,00	40,00	40,00	40,00	40,00	40,00
40,00	33,13	40,00	40,00	40,00	40,00	40,00	40,00	40,00
40,00	40,00	40,00	40,00	40,00	40,00	40,00	40,00	40,00
40,00	29,93	40,00	40,00	40,00	40,00	40,00	40,00	40,00
40,00	30,91	40,00	40,00	40,00	40,00	40,00	40,00	40,00
40,00	40,00	40,00	40,00	40,00	40,00	40,00	40,00	40,00
40,00	33,49	40,00	40,00	40,00	40,00	40,00	35,72	40,00
40,00	32,57	40,00	40,00	40,00	40,00	40,00	40,00	40,00
40,00	32,05	40,00	40,00	40,00	40,00	40,00	40,00	40,00
40,00	40,00	40,00	40,00	40,00	40,00	40,00	40,00	40,00
40,00	34,01	40,00	40,00	40,00	40,00	40,00	40,00	40,00
40,00	40,00	40,00	40,00	40,00	40,00	40,00	40,00	40,00
40,00	40,00	40,00	40,00	40,00	40,00	40,00	40,00	40,00
34,15	34,55	40,00	40,00	40,00	40,00	40,00	40,00	40,00
40,00	40,00	40,00	40,00	40,00	40,00	40,00	40,00	40,00
40,00	40,00	40,00	40,00	40,00	40,00	40,00	40,00	40,00
40,00	40,00	40,00	40,00	40,00	40,00	40,00	40,00	40,00
40,00	31,76	40,00	40,00	40,00	40,00	40,00	40,00	40,00
40,00	33,46	40,00	40,00	40,00	40,00	40,00	40,00	40,00
40,00	32,41	40,00	40,00	40,00	40,00	40,00	40,00	40,00
40,00	34,62	40,00	40,00	40,00	40,00	40,00	40,00	40,00
40,00	31,19	40,00	40,00	40,00	40,00	40,00	40,00	40,00
40,00	35,35	40,00	40,00	40,00	40,00	40,00	40,00	40,00
40,00	40,00	40,00	40,00	40,00	40,00	40,00	40,00	40,00
40,00	40,00	40,00	40,00	40,00	40,00	40,00	40,00	40,00
40,00	30,47	40,00	40,00	40,00	40,00	40,00	40,00	40,00
40,00	31,32	40,00	40,00	40,00	40,00	40,00	35,92	40,00
40,00	33,85	40,00	40,00	40,00	40,00	40,00	40,00	40,00
40,00	32,40	40,00	40,00	40,00	40,00	40,00	40,00	40,00
40,00	40,00	40,00	40,00	40,00	40,00	40,00	40,00	40,00
40,00	32,76	40,00	40,00	40,00	40,00	40,00	38,08	40,00
40,00	33,44	40,00	40,00	40,00	40,00	40,00	28,98	40,00
40,00	40,00	40,00	40,00	40,00	40,00	40,00	40,00	40,00
40,00	33,73	40,00	40,00	40,00	40,00	40,00	40,00	40,00
40,00	34,59	40,00	40,00	40,00	40,00	40,00	40,00	40,00
40,00	35,85	40,00	40,00	40,00	40,00	40,00	40,00	40,00
40,00	40,00	40,00	40,00	40,00	40,00	40,00	40,00	40,00

hsa-miR-511	hsa-miR-512-3p	hsa-miR-512-5p	hsa-miR-513-5p	hsa-miR-515-3p	hsa-miR-515-5p	hsa-miR-516a-5p	hsa-miR-516b	hsa-miR-517a
40,00	40,00	40,00	40,00	40,00	40,00	40,00	40,00	40,00
40,00	40,00	40,00	40,00	40,00	40,00	40,00	40,00	40,00
40,00	40,00	40,00	40,00	40,00	40,00	40,00	40,00	36,37
40,00	40,00	40,00	40,00	40,00	40,00	40,00	40,00	40,00
40,00	40,00	40,00	40,00	40,00	40,00	40,00	40,00	40,00
33,08	40,00	40,00	40,00	40,00	40,00	40,00	40,00	40,00
40,00	40,00	40,00	40,00	40,00	40,00	40,00	40,00	40,00
40,00	40,00	40,00	40,00	40,00	40,00	40,00	40,00	35,09
32,89	40,00	40,00	40,00	40,00	40,00	40,00	40,00	36,48
40,00	34,41	40,00	40,00	40,00	40,00	40,00	40,00	36,91
40,00	40,00	40,00	40,00	40,00	40,00	40,00	40,00	40,00
40,00	40,00	40,00	40,00	40,00	40,00	40,00	40,00	40,00
40,00	40,00	40,00	40,00	40,00	40,00	40,00	40,00	40,00
40,00	40,00	40,00	40,00	40,00	40,00	40,00	40,00	34,82
40,00	40,00	40,00	40,00	40,00	40,00	40,00	40,00	40,00
40,00	37,01	40,00	40,00	40,00	40,00	40,00	40,00	40,00
40,00	40,00	40,00	40,00	40,00	40,00	40,00	40,00	40,00
40,00	40,00	40,00	40,00	40,00	40,00	40,00	40,00	40,00
31,93	40,00	40,00	40,00	40,00	40,00	40,00	40,00	40,00
40,00	40,00	40,00	40,00	40,00	40,00	40,00	40,00	40,00
40,00	40,00	40,00	40,00	40,00	40,00	40,00	40,00	40,00
32,02	40,00	40,00	40,00	40,00	40,00	40,00	40,00	40,00
33,30	40,00	40,00	40,00	40,00	40,00	40,00	40,00	40,00
33,74	40,00	40,00	40,00	40,00	40,00	40,00	40,00	40,00
33,23	33,92	40,00	40,00	40,00	40,00	40,00	40,00	40,00
40,00	40,00	40,00	40,00	40,00	40,00	40,00	40,00	40,00
40,00	35,46	40,00	40,00	40,00	40,00	40,00	40,00	40,00
33,83	40,00	40,00	40,00	40,00	40,00	40,00	40,00	40,00
40,00	40,00	40,00	40,00	40,00	40,00	40,00	40,00	40,00
35,72	40,00	40,00	40,00	40,00	40,00	40,00	40,00	40,00
32,94	40,00	40,00	40,00	40,00	40,00	40,00	40,00	39,36
33,51	39,95	40,00	40,00	40,00	40,00	40,00	40,00	33,68
32,87	40,00	40,00	40,00	40,00	40,00	40,00	40,00	40,00
33,80	38,91	40,00	40,00	40,00	40,00	40,00	40,00	40,00
32,84	40,00	40,00	40,00	40,00	40,00	40,00	40,00	40,00
40,00	40,00	40,00	40,00	40,00	40,00	40,00	40,00	40,00
40,00	40,00	40,00	40,00	40,00	40,00	40,00	40,00	37,27
34,35	40,00	40,00	40,00	40,00	39,22	40,00	40,00	34,18
32,96	40,00	40,00	40,00	40,00	40,00	40,00	40,00	40,00
40,00	40,00	40,00	40,00	40,00	40,00	40,00	40,00	39,16
34,22	40,00	40,00	40,00	40,00	40,00	40,00	40,00	40,00
40,00	40,00	40,00	40,00	40,00	40,00	40,00	40,00	40,00

hsa-miR-517b	hsa-miR-517c	hsa-miR-518a-3p	hsa-miR-518a-5p	hsa-miR-518b	hsa-miR-518c	hsa-miR-518d	hsa-miR-518d-5p	hsa-miR-518e
40,00	40,00	40,00	40,00	40,00	40,00	40,00	40,00	40,00
40,00	40,00	40,00	40,00	40,00	40,00	40,00	40,00	40,00
40,00	37,18	40,00	40,00	40,00	40,00	40,00	40,00	40,00
40,00	40,00	40,00	40,00	40,00	40,00	40,00	40,00	40,00
40,00	40,00	40,00	40,00	40,00	40,00	40,00	40,00	40,00
40,00	40,00	40,00	40,00	40,00	40,00	39,24	40,00	40,00
40,00	40,00	40,00	40,00	40,00	40,00	40,00	40,00	40,00
40,00	40,00	40,00	40,00	40,00	40,00	40,00	40,00	40,00
40,00	40,00	40,00	40,00	40,00	40,00	40,00	40,00	40,00
40,00	40,00	40,00	40,00	40,00	40,00	40,00	40,00	40,00
40,00	38,28	40,00	40,00	40,00	40,00	40,00	40,00	40,00
40,00	40,00	40,00	40,00	40,00	40,00	40,00	40,00	40,00
40,00	40,00	40,00	40,00	40,00	40,00	37,05	40,00	40,00
40,00	36,78	40,00	40,00	40,00	40,00	40,00	40,00	40,00
40,00	40,00	40,00	40,00	40,00	40,00	36,07	40,00	40,00
40,00	40,00	40,00	40,00	40,00	40,00	40,00	40,00	40,00
40,00	40,00	40,00	40,00	40,00	40,00	40,00	40,00	40,00
40,00	40,00	40,00	40,00	40,00	40,00	40,00	40,00	40,00
40,00	40,00	40,00	40,00	31,04	40,00	40,00	40,00	40,00
40,00	40,00	40,00	40,00	30,84	40,00	40,00	40,00	40,00
40,00	40,00	40,00	40,00	32,89	40,00	40,00	40,00	40,00
40,00	40,00	40,00	40,00	30,78	40,00	40,00	40,00	40,00
40,00	40,00	40,00	40,00	28,39	40,00	40,00	40,00	40,00
40,00	33,94	40,00	40,00	29,38	40,00	40,00	40,00	40,00
40,00	40,00	40,00	40,00	28,54	40,00	40,00	40,00	40,00
40,00	40,00	40,00	40,00	29,40	40,00	36,78	40,00	40,00
40,00	36,52	40,00	40,00	29,75	40,00	40,00	40,00	33,08
40,00	40,00	40,00	40,00	34,38	40,00	40,00	40,00	40,00
40,00	40,00	40,00	40,00	33,16	40,00	40,00	40,00	40,00
40,00	40,00	40,00	40,00	35,13	40,00	40,00	40,00	34,20
40,00	34,30	40,00	40,00	32,76	40,00	40,00	40,00	40,00
33,05	40,00	40,00	37,51	32,88	40,00	40,00	40,00	40,00
40,00	40,00	40,00	40,00	31,44	40,00	40,00	40,00	40,00
40,00	40,00	40,00	40,00	32,84	40,00	40,00	40,00	40,00
40,00	40,00	40,00	40,00	29,69	40,00	40,00	40,00	40,00
40,00	40,00	40,00	40,00	29,94	40,00	40,00	40,00	40,00
40,00	40,00	40,00	40,00	32,37	40,00	40,00	40,00	40,00
40,00	40,00	40,00	40,00	31,80	40,00	38,32	40,00	40,00
40,00	35,39	40,00	40,00	30,02	40,00	40,00	40,00	40,00
40,00	40,00	40,00	40,00	32,81	40,00	40,00	40,00	40,00
40,00	40,00	40,00	40,00	28,21	40,00	40,00	40,00	40,00
40,00	40,00	40,00	40,00	33,47	40,00	40,00	40,00	40,00

hsa-miR-518f	hsa-miR-519	hsa-miR-519c	hsa-miR-519d	hsa-miR-519e	hsa-miR-520a	hsa-miR-520a#	hsa-miR-520b	hsa-miR-520d-5p
40,00	40,00	40,00	40,00	40,00	40,00	40,00	40,00	40,00
40,00	40,00	40,00	40,00	40,00	40,00	40,00	40,00	40,00
40,00	40,00	40,00	40,00	40,00	40,00	40,00	40,00	40,00
40,00	40,00	40,00	40,00	40,00	40,00	40,00	40,00	40,00
40,00	40,00	40,00	40,00	40,00	40,00	40,00	40,00	40,00
40,00	40,00	40,00	40,00	40,00	40,00	40,00	40,00	40,00
40,00	39,61	40,00	40,00	40,00	40,00	40,00	40,00	40,00
40,00	40,00	40,00	40,00	40,00	40,00	40,00	40,00	40,00
40,00	33,35	40,00	40,00	40,00	40,00	40,00	40,00	40,00
40,00	40,00	40,00	32,81	40,00	40,00	40,00	40,00	40,00
40,00	40,00	40,00	40,00	40,00	40,00	40,00	40,00	40,00
40,00	40,00	40,00	40,00	40,00	36,68	40,00	40,00	34,61
32,78	39,72	40,00	40,00	40,00	40,00	40,00	40,00	40,00
40,00	40,00	40,00	40,00	40,00	40,00	40,00	40,00	40,00
40,00	40,00	40,00	40,00	40,00	40,00	40,00	40,00	35,65
40,00	40,00	40,00	40,00	40,00	40,00	40,00	40,00	36,95
40,00	40,00	40,00	40,00	40,00	40,00	40,00	40,00	40,00
40,00	40,00	40,00	40,00	40,00	40,00	40,00	38,10	40,00
40,00	40,00	40,00	40,00	40,00	40,00	40,00	40,00	40,00
40,00	40,00	40,00	40,00	40,00	40,00	40,00	40,00	40,00
40,00	40,00	40,00	40,00	40,00	40,00	40,00	40,00	40,00
40,00	36,57	40,00	40,00	40,00	40,00	40,00	40,00	40,00
40,00	40,00	40,00	40,00	40,00	40,00	40,00	40,00	40,00
40,00	37,36	40,00	40,00	40,00	40,00	40,00	40,00	40,00
40,00	40,00	40,00	40,00	40,00	40,00	40,00	40,00	40,00
40,00	40,00	40,00	40,00	40,00	40,00	40,00	40,00	39,18
40,00	40,00	40,00	40,00	40,00	40,00	40,00	36,85	36,36
34,13	40,00	40,00	40,00	40,00	40,00	40,00	40,00	33,63
40,00	40,00	40,00	40,00	40,00	40,00	40,00	40,00	40,00
40,00	40,00	40,00	40,00	40,00	40,00	40,00	40,00	40,00
40,00	40,00	40,00	40,00	40,00	40,00	40,00	40,00	40,00
34,34	40,00	40,00	40,00	40,00	40,00	40,00	40,00	40,00
40,00	40,00	40,00	40,00	40,00	40,00	40,00	40,00	40,00
40,00	40,00	40,00	33,92	40,00	40,00	40,00	40,00	34,33
40,00	40,00	40,00	40,00	40,00	40,00	40,00	40,00	40,00
40,00	40,00	40,00	40,00	40,00	40,00	40,00	40,00	40,00
40,00	38,93	40,00	40,00	40,00	40,00	40,00	40,00	40,00
40,00	40,00	40,00	40,00	40,00	40,00	40,00	40,00	40,00
40,00	40,00	40,00	40,00	40,00	40,00	40,00	40,00	40,00
40,00	40,00	40,00	40,00	40,00	40,00	40,00	40,00	40,00
33,14	40,00	40,00	29,85	40,00	40,00	39,02	40,00	40,00
40,00	40,00	40,00	40,00	40,00	40,00	40,00	40,00	40,00

hsa-miR-520e	hsa-miR-520f	hsa-miR-520g	hsa-miR-521	hsa-miR-522	hsa-miR-523	hsa-miR-524-5p	hsa-miR-525	hsa-miR-525-3p
40,00	40,00	40,00	40,00	40,00	40,00	40,00	40,00	40,00
40,00	40,00	40,00	40,00	40,00	40,00	40,00	40,00	40,00
40,00	40,00	40,00	40,00	40,00	40,00	40,00	40,00	40,00
40,00	40,00	40,00	39,40	40,00	40,00	40,00	40,00	40,00
40,00	40,00	40,00	40,00	40,00	40,00	40,00	40,00	40,00
40,00	40,00	40,00	40,00	40,00	40,00	40,00	40,00	40,00
40,00	40,00	40,00	40,00	40,00	40,00	40,00	40,00	40,00
40,00	40,00	40,00	40,00	38,80	40,00	40,00	40,00	40,00
40,00	40,00	40,00	40,00	40,00	40,00	40,00	40,00	40,00
40,00	40,00	40,00	40,00	40,00	40,00	40,00	40,00	40,00
40,00	40,00	36,76	40,00	40,00	37,31	40,00	40,00	40,00
40,00	40,00	40,00	40,00	36,55	40,00	40,00	40,00	40,00
40,00	40,00	40,00	40,00	40,00	40,00	40,00	40,00	40,00
40,00	40,00	40,00	40,00	40,00	40,00	40,00	40,00	40,00
40,00	40,00	40,00	40,00	40,00	40,00	40,00	40,00	40,00
40,00	40,00	40,00	40,00	40,00	40,00	40,00	40,00	40,00
40,00	40,00	40,00	40,00	40,00	40,00	40,00	40,00	40,00
40,00	40,00	40,00	40,00	40,00	37,05	40,00	40,00	40,00
40,00	40,00	38,16	40,00	40,00	39,93	40,00	40,00	40,00
40,00	40,00	40,00	40,00	40,00	40,00	40,00	40,00	40,00
40,00	40,00	40,00	40,00	40,00	39,53	40,00	40,00	40,00
40,00	40,00	40,00	40,00	40,00	38,68	40,00	40,00	40,00
40,00	40,00	40,00	40,00	40,00	37,09	40,00	40,00	40,00
40,00	40,00	40,00	40,00	40,00	37,85	40,00	40,00	40,00
40,00	40,00	40,00	40,00	40,00	38,23	40,00	39,98	40,00
40,00	40,00	40,00	40,00	40,00	40,00	40,00	40,00	40,00
40,00	40,00	40,00	40,00	40,00	40,00	40,00	38,94	40,00
40,00	40,00	40,00	40,00	40,00	35,81	40,00	36,46	40,00
40,00	40,00	40,00	40,00	40,00	36,82	40,00	40,00	40,00
40,00	40,00	40,00	40,00	40,00	38,69	40,00	40,00	40,00
40,00	40,00	40,00	40,00	40,00	36,26	40,00	40,00	40,00
40,00	40,00	40,00	40,00	40,00	40,00	40,00	40,00	40,00
40,00	40,00	40,00	40,00	40,00	38,11	40,00	40,00	40,00
40,00	40,00	40,00	40,00	40,00	39,86	40,00	40,00	40,00
40,00	40,00	40,00	40,00	40,00	39,64	40,00	40,00	40,00
40,00	40,00	40,00	40,00	40,00	38,10	40,00	40,00	40,00
40,00	40,00	40,00	40,00	40,00	38,77	40,00	40,00	36,14
40,00	40,00	40,00	40,00	40,00	38,73	40,00	40,00	40,00
40,00	40,00	40,00	40,00	40,00	39,11	40,00	40,00	40,00
40,00	40,00	40,00	40,00	40,00	37,37	40,00	40,00	40,00
40,00	40,00	40,00	40,00	40,00	34,97	40,00	40,00	40,00
40,00	40,00	40,00	40,00	40,00	40,00	40,00	40,00	40,00

hsa-miR-526b	hsa-miR-532	hsa-miR-532-3p	hsa-miR-539	hsa-miR-541	hsa-miR-542-3p	hsa-miR-542-5p	hsa-miR-544	hsa-miR-545
40,00	30,56	31,08	31,27	40,00	40,00	40,00	40,00	31,03
40,00	31,58	32,26	30,00	40,00	40,00	40,00	40,00	40,00
40,00	29,14	31,85	28,28	40,00	40,00	40,00	40,00	32,53
40,00	27,06	30,36	27,51	40,00	40,00	40,00	40,00	34,89
40,00	29,26	31,24	27,72	40,00	40,00	40,00	40,00	40,00
40,00	29,75	32,33	28,83	40,00	40,00	40,00	40,00	33,03
40,00	32,42	31,62	31,29	40,00	40,00	40,00	40,00	40,00
40,00	28,69	30,28	29,62	33,49	40,00	40,00	40,00	31,19
40,00	25,82	27,14	24,32	32,04	32,96	35,91	33,70	29,65
40,00	25,98	27,46	27,81	40,00	40,00	35,85	40,00	28,95
40,00	29,18	29,53	30,80	40,00	40,00	40,00	40,00	40,00
40,00	30,41	30,58	29,38	40,00	40,00	40,00	40,00	40,00
40,00	27,36	28,61	28,37	40,00	40,00	40,00	40,00	31,03
40,00	28,85	31,20	32,58	40,00	40,00	40,00	40,00	34,42
40,00	30,11	32,79	31,02	40,00	40,00	40,00	40,00	30,94
40,00	30,61	32,08	32,13	40,00	40,00	40,00	40,00	40,00
40,00	30,99	31,91	32,41	40,00	40,00	40,00	40,00	40,00
40,00	40,00	33,96	40,00	40,00	40,00	40,00	40,00	40,00
40,00	30,22	31,26	30,73	40,00	40,00	40,00	40,00	32,51
40,00	32,55	31,86	32,29	40,00	40,00	40,00	40,00	40,00
40,00	31,28	33,21	30,19	40,00	40,00	40,00	40,00	40,00
40,00	29,04	30,89	31,33	40,00	40,00	40,00	40,00	40,00
40,00	27,31	29,99	31,68	40,00	40,00	40,00	40,00	40,00
40,00	27,64	29,76	30,14	40,00	40,00	36,22	40,00	31,83
40,00	26,74	28,66	33,16	40,00	40,00	40,00	40,00	33,20
40,00	29,18	31,10	33,62	40,00	40,00	40,00	40,00	35,87
40,00	26,04	28,46	33,13	40,00	40,00	40,00	40,00	40,00
40,00	26,89	29,23	29,23	40,00	40,00	40,00	40,00	34,69
40,00	29,73	31,42	30,30	40,00	40,00	40,00	40,00	32,72
40,00	28,31	29,07	30,32	40,00	40,00	40,00	40,00	40,00
40,00	26,35	28,17	29,24	40,00	40,00	37,81	40,00	32,76
40,00	26,25	28,28	29,65	40,00	40,00	40,00	40,00	33,61
40,00	26,79	27,72	26,69	40,00	40,00	40,00	40,00	33,70
40,00	26,62	28,50	28,99	38,24	40,00	40,00	40,00	33,71
40,00	28,61	30,89	33,30	40,00	40,00	40,00	40,00	40,00
40,00	28,59	30,22	28,31	40,00	40,00	40,00	40,00	31,98
40,00	28,00	29,49	30,08	40,00	40,00	40,00	40,00	40,00
40,00	28,40	30,09	31,82	40,00	40,00	40,00	40,00	32,21
40,00	27,06	28,99	33,52	40,00	40,00	35,78	40,00	40,00
40,00	27,48	28,75	27,93	38,02	40,00	40,00	40,00	34,28
24,44	28,59	29,75	30,92	40,00	40,00	40,00	40,00	35,77
40,00	30,09	32,50	32,58	40,00	40,00	40,00	40,00	40,00

hsa-miR-548a	hsa-miR-548a-5p	hsa-miR-548b	hsa-miR-548b-5p	hsa-miR-548c	hsa-miR-548c-5p	hsa-miR-548d	hsa-miR-548d-5p	hsa-miR-551b
40,00	40,00	40,00	40,00	40,00	40,00	40,00	40,00	40,00
40,00	40,00	40,00	40,00	40,00	40,00	40,00	40,00	40,00
40,00	40,00	40,00	40,00	40,00	40,00	40,00	40,00	40,00
40,00	40,00	40,00	40,00	33,68	40,00	40,00	40,00	40,00
40,00	40,00	40,00	40,00	40,00	40,00	40,00	34,98	40,00
40,00	40,00	40,00	40,00	40,00	40,00	40,00	40,00	40,00
40,00	40,00	40,00	40,00	40,00	40,00	40,00	40,00	40,00
40,00	40,00	40,00	40,00	40,00	40,00	40,00	40,00	40,00
40,00	38,34	40,00	38,61	33,51	33,43	40,00	32,35	32,54
40,00	40,00	40,00	40,00	40,00	40,00	33,73	40,00	33,76
40,00	40,00	40,00	40,00	40,00	40,00	40,00	40,00	40,00
40,00	40,00	40,00	40,00	36,72	40,00	33,52	40,00	40,00
40,00	40,00	40,00	40,00	40,00	33,34	40,00	33,99	40,00
40,00	40,00	40,00	40,00	40,00	40,00	40,00	40,00	40,00
40,00	40,00	40,00	40,00	40,00	40,00	40,00	40,00	40,00
40,00	40,00	40,00	40,00	40,00	33,28	40,00	36,21	33,74
40,00	40,00	40,00	40,00	40,00	40,00	40,00	37,98	40,00
40,00	40,00	40,00	40,00	40,00	40,00	40,00	40,00	40,00
40,00	40,00	40,00	40,00	33,79	40,00	40,00	40,00	40,00
40,00	40,00	40,00	40,00	33,73	40,00	40,00	40,00	40,00
40,00	40,00	40,00	40,00	34,64	40,00	40,00	40,00	40,00
40,00	40,00	40,00	40,00	40,00	40,00	40,00	40,00	40,00
40,00	40,00	40,00	40,00	40,00	40,00	40,00	40,00	40,00
40,00	40,00	40,00	40,00	40,00	34,14	40,00	33,70	40,00
40,00	40,00	40,00	40,00	40,00	40,00	40,00	33,46	40,00
40,00	40,00	40,00	40,00	33,96	40,00	40,00	40,00	40,00
36,16	40,00	40,00	40,00	40,00	40,00	40,00	40,00	40,00
40,00	40,00	40,00	40,00	40,00	34,43	40,00	40,00	34,55
33,65	40,00	40,00	40,00	40,00	40,00	40,00	40,00	40,00
40,00	40,00	40,00	40,00	40,00	40,00	40,00	40,00	33,21
40,00	40,00	40,00	40,00	40,00	34,17	40,00	40,00	32,83
40,00	40,00	40,00	40,00	40,00	40,00	40,00	40,00	31,55
40,00	40,00	40,00	40,00	40,00	33,44	40,00	40,00	33,72
40,00	40,00	40,00	40,00	40,00	40,00	40,00	40,00	40,00
40,00	40,00	40,00	40,00	40,00	33,96	40,00	35,77	40,00
40,00	40,00	40,00	40,00	40,00	34,71	40,00	34,18	40,00
40,00	40,00	40,00	40,00	40,00	40,00	40,00	40,00	40,00
40,00	40,00	40,00	40,00	40,00	40,00	40,00	40,00	40,00
40,00	40,00	40,00	39,47	34,78	33,09	34,49	33,85	34,74
40,00	40,00	40,00	40,00	34,79	40,00	40,00	40,00	40,00
40,00	40,00	40,00	40,00	34,81	40,00	40,00	40,00	40,00
40,00	40,00	40,00	40,00	34,87	40,00	40,00	40,00	40,00

hsa-miR-556-3p	hsa-miR-556-5p	hsa-miR-561	hsa-miR-570	hsa-miR-574-3p	hsa-miR-576-3p	hsa-miR-576-5p	hsa-miR-579	hsa-miR-582-3p
40,00	40,00	40,00	40,00	25,80	40,00	40,00	40,00	40,00
40,00	40,00	40,00	40,00	29,22	40,00	40,00	40,00	40,00
40,00	40,00	40,00	34,30	25,22	40,00	40,00	33,71	40,00
40,00	40,00	40,00	40,00	23,73	34,19	32,86	40,00	40,00
40,00	40,00	40,00	40,00	25,00	40,00	40,00	40,00	40,00
40,00	40,00	40,00	40,00	25,87	33,56	40,00	40,00	40,00
40,00	40,00	40,00	40,00	26,99	40,00	40,00	40,00	40,00
40,00	40,00	40,00	40,00	25,42	32,14	34,44	40,00	40,00
40,00	40,00	35,43	34,61	22,47	31,15	40,00	30,01	40,00
40,00	40,00	40,00	33,32	23,62	40,00	37,25	29,97	40,00
40,00	40,00	36,76	40,00	25,80	39,63	40,00	32,28	40,00
40,00	40,00	40,00	40,00	25,88	33,56	40,00	33,34	40,00
40,00	40,00	36,60	40,00	24,95	33,44	40,00	31,82	40,00
40,00	40,00	37,40	40,00	26,01	40,00	40,00	31,87	40,00
40,00	40,00	40,00	40,00	27,34	40,00	40,00	40,00	40,00
40,00	40,00	40,00	40,00	27,25	40,00	40,00	40,00	40,00
40,00	40,00	40,00	40,00	27,00	40,00	40,00	32,83	40,00
40,00	40,00	40,00	40,00	28,31	40,00	40,00	40,00	40,00
40,00	40,00	40,00	40,00	26,12	33,36	40,00	33,37	40,00
40,00	40,00	40,00	40,00	28,29	40,00	40,00	40,00	40,00
40,00	40,00	40,00	40,00	26,78	40,00	40,00	33,57	40,00
40,00	40,00	40,00	40,00	26,59	38,61	40,00	33,67	40,00
40,00	40,00	40,00	40,00	27,25	40,00	40,00	40,00	40,00
40,00	40,00	40,00	40,00	25,55	33,26	38,51	31,96	34,74
40,00	40,00	40,00	40,00	26,33	40,00	40,00	32,38	40,00
40,00	40,00	40,00	40,00	28,43	33,69	40,00	33,57	40,00
40,00	40,00	40,00	40,00	26,09	33,75	40,00	30,77	40,00
40,00	40,00	37,83	40,00	25,89	33,62	40,00	31,19	40,00
40,00	40,00	40,00	40,00	27,91	35,96	40,00	40,00	40,00
40,00	40,00	40,00	40,00	25,94	40,00	40,00	34,44	40,00
40,00	40,00	40,00	40,00	25,26	31,56	40,00	30,75	40,00
40,00	40,00	40,00	40,00	25,80	33,01	40,00	32,54	40,00
40,00	40,00	40,00	40,00	24,91	31,98	40,00	34,61	40,00
40,00	40,00	40,00	40,00	25,73	32,18	36,75	34,20	40,00
40,00	40,00	40,00	40,00	27,90	36,39	40,00	33,35	40,00
40,00	40,00	40,00	40,00	25,76	32,91	35,04	32,56	40,00
40,00	40,00	40,00	40,00	24,68	40,00	40,00	40,00	40,00
40,00	40,00	40,00	40,00	26,66	34,50	40,00	40,00	40,00
40,00	40,00	40,00	40,00	24,97	31,60	40,00	40,00	40,00
40,00	40,00	40,00	35,49	24,17	33,40	40,00	32,82	40,00
40,00	40,00	40,00	40,00	27,80	28,78	40,00	36,36	40,00
40,00	40,00	40,00	40,00	27,75	34,68	40,00	35,40	40,00

hsa-miR-582-5p	hsa-miR-589	hsa-miR-590-5p	hsa-miR-597	hsa-miR-598	hsa-miR-615-5p	hsa-miR-616	hsa-miR-618	hsa-miR-624	hsa-miR-625
40,00	40,00	28,33	40,00	30,88	40,00	40,00	40,00	40,00	35,99
40,00	40,00	32,15	40,00	40,00	40,00	40,00	40,00	40,00	33,93
40,00	40,00	27,98	40,00	30,50	40,00	40,00	34,01	40,00	36,13
40,00	40,00	26,30	40,00	29,06	40,00	33,70	40,00	40,00	30,73
40,00	40,00	28,16	40,00	31,22	40,00	40,00	34,97	40,00	33,52
40,00	40,00	28,64	40,00	31,26	40,00	31,58	35,29	40,00	33,35
40,00	40,00	29,38	40,00	32,50	40,00	40,00	31,25	40,00	40,00
40,00	40,00	27,32	40,00	30,49	40,00	40,00	33,61	40,00	32,67
40,00	34,39	24,57	40,00	28,12	40,00	32,76	31,36	40,00	28,91
40,00	33,41	24,89	31,65	28,53	40,00	32,76	34,46	40,00	30,11
39,09	40,00	26,21	33,25	30,24	40,00	40,00	33,71	40,00	32,44
40,00	40,00	27,35	34,87	31,43	40,00	40,00	34,46	40,00	33,43
40,00	40,00	25,92	40,00	29,31	40,00	32,60	32,62	40,00	31,72
40,00	40,00	26,79	40,00	32,53	40,00	40,00	32,78	40,00	34,36
40,00	40,00	28,18	35,65	33,39	40,00	40,00	40,00	40,00	33,98
40,00	40,00	27,77	40,00	31,56	40,00	33,95	34,78	40,00	34,80
40,00	40,00	27,75	40,00	30,47	40,00	40,00	40,00	40,00	40,00
40,00	40,00	31,02	40,00	40,00	40,00	40,00	40,00	40,00	40,00
40,00	40,00	28,83	40,00	32,42	40,00	33,75	35,87	40,00	33,63
40,00	40,00	30,16	40,00	40,00	40,00	40,00	29,94	40,00	40,00
40,00	40,00	28,36	40,00	34,53	40,00	40,00	31,30	40,00	33,91
40,00	40,00	28,19	40,00	30,09	40,00	40,00	31,51	40,00	35,62
40,00	34,36	29,16	33,71	33,48	40,00	40,00	36,55	40,00	35,75
40,00	35,37	27,33	33,55	29,81	40,00	40,00	33,08	40,00	32,32
40,00	33,17	27,18	32,51	30,73	40,00	40,00	34,38	40,00	33,96
40,00	40,00	28,84	31,89	32,12	40,00	40,00	35,05	40,00	34,74
40,00	40,00	27,13	30,94	29,96	40,00	40,00	32,41	40,00	34,03
40,00	40,00	27,17	34,40	30,27	40,00	35,93	33,65	40,00	35,10
40,00	40,00	28,97	40,00	32,53	40,00	40,00	30,60	40,00	34,43
40,00	40,00	27,51	33,45	31,33	40,00	40,00	33,89	40,00	33,21
40,00	35,67	25,88	31,25	29,89	40,00	40,00	30,43	40,00	31,40
40,00	34,46	26,54	34,67	29,93	40,00	40,00	34,71	40,00	33,00
40,00	40,00	25,86	33,51	28,74	40,00	33,03	33,29	40,00	31,86
40,00	34,21	26,94	38,07	29,94	40,00	40,00	33,75	40,00	32,77
40,00	33,56	28,93	40,00	31,95	40,00	40,00	36,60	40,00	40,00
40,00	40,00	27,79	33,00	29,91	40,00	34,55	33,53	40,00	33,09
40,00	40,00	27,40	40,00	30,74	40,00	35,39	34,50	40,00	33,40
40,00	34,62	28,66	40,00	31,82	40,00	40,00	35,23	40,00	35,09
40,00	34,96	26,64	33,04	29,83	40,00	40,00	35,80	40,00	32,93
40,00	40,00	27,17	40,00	29,57	40,00	40,00	33,05	40,00	35,62
40,00	40,00	28,52	31,36	31,01	40,00	40,00	35,17	40,00	33,80
40,00	40,00	29,75	40,00	32,35	40,00	40,00	31,51	40,00	34,77

hsa-miR-627	hsa-miR-628-5p	hsa-miR-629	hsa-miR-636	hsa-miR-642	hsa-miR-651	hsa-miR-652	hsa-miR-653	hsa-miR-654	hsa-miR-654-3p
40,00	40,00	40,00	40,00	40,00	40,00	33,62	40,00	40,00	40,00
40,00	40,00	40,00	34,11	40,00	40,00	40,00	40,00	40,00	40,00
40,00	29,66	33,37	31,52	40,00	40,00	29,88	40,00	40,00	40,00
40,00	28,03	37,67	31,06	40,00	40,00	27,96	40,00	40,00	40,00
40,00	29,81	40,00	33,74	40,00	40,00	28,12	40,00	40,00	40,00
40,00	29,37	33,77	34,21	40,00	40,00	30,11	40,00	40,00	40,00
40,00	29,36	40,00	34,48	40,00	40,00	30,75	40,00	40,00	40,00
34,13	31,02	33,08	33,77	40,00	40,00	26,93	40,00	40,00	33,14
30,91	26,58	28,64	30,06	40,00	40,00	23,76	40,00	30,87	29,63
33,24	28,34	33,44	30,88	31,76	40,00	24,23	40,00	40,00	32,81
31,96	30,97	34,26	32,64	40,00	40,00	26,97	40,00	40,00	40,00
40,00	28,81	34,74	40,00	40,00	40,00	28,09	40,00	40,00	40,00
40,00	27,81	30,56	32,97	40,00	40,00	25,61	40,00	40,00	32,13
32,61	28,72	30,94	31,72	34,20	40,00	27,37	40,00	40,00	40,00
40,00	32,62	38,94	40,00	40,00	40,00	29,57	40,00	40,00	40,00
40,00	26,88	31,28	32,18	40,00	40,00	28,35	40,00	40,00	40,00
40,00	30,26	38,31	35,20	40,00	40,00	30,03	40,00	40,00	40,00
40,00	32,62	40,00	40,00	40,00	40,00	38,03	40,00	40,00	40,00
33,94	29,51	33,82	40,00	40,00	40,00	28,56	40,00	40,00	32,62
40,00	27,43	40,00	35,55	40,00	40,00	32,04	40,00	40,00	40,00
40,00	27,19	33,94	40,00	40,00	40,00	31,85	40,00	40,00	40,00
34,43	25,03	35,16	39,83	33,14	40,00	27,97	40,00	38,24	40,00
40,00	24,63	31,97	35,34	33,60	40,00	27,83	40,00	40,00	40,00
35,13	22,24	40,00	32,89	40,00	40,00	26,88	40,00	38,04	33,78
40,00	24,01	33,82	34,19	32,16	40,00	25,67	40,00	39,96	40,00
40,00	22,85	40,00	40,00	40,00	40,00	29,21	40,00	40,00	40,00
40,00	24,85	32,30	32,60	32,47	40,00	26,83	40,00	38,80	40,00
40,00	24,55	32,19	40,00	34,32	40,00	26,90	40,00	33,20	34,04
40,00	22,96	33,69	35,08	32,72	40,00	30,51	40,00	40,00	40,00
40,00	26,89	36,47	32,55	34,27	40,00	27,40	40,00	38,77	33,97
34,10	23,20	31,68	34,20	33,41	40,00	25,97	40,00	34,01	32,15
33,36	25,24	33,42	33,75	33,15	40,00	24,85	40,00	34,86	40,00
35,31	23,56	40,00	33,81	31,84	40,00	26,05	40,00	32,55	32,82
40,00	24,96	35,29	33,55	33,06	40,00	25,78	40,00	32,38	40,00
40,00	23,67	34,34	35,26	40,00	40,00	28,11	40,00	40,00	40,00
36,91	23,08	33,51	34,22	35,14	40,00	27,56	40,00	38,01	32,76
34,02	24,33	32,68	34,92	34,03	40,00	27,54	40,00	40,00	40,00
40,00	23,02	31,52	33,57	40,00	40,00	28,69	40,00	40,00	40,00
37,76	27,20	31,09	32,59	34,08	40,00	26,43	40,00	40,00	40,00
31,97	25,50	35,27	33,36	35,25	40,00	26,22	40,00	32,80	40,00
40,00	11,81	34,47	33,04	35,11	40,00	28,46	40,00	34,44	40,00
40,00	24,08	34,60	37,76	32,80	40,00	31,01	40,00	40,00	40,00

hsa-miR-655	hsa-miR-660	hsa-miR-671-3p	hsa-miR-672	hsa-miR-674	hsa-miR-708	hsa-miR-744	hsa-miR-758	hsa-miR-871	hsa-miR-872
40,00	36,37	40,00	40,00	40,00	40,00	30,60	40,00	40,00	40,00
40,00	40,00	40,00	40,00	40,00	40,00	40,00	40,00	40,00	40,00
40,00	29,56	32,11	40,00	40,00	40,00	28,21	33,26	40,00	40,00
37,84	32,40	29,35	40,00	40,00	40,00	26,31	40,00	40,00	40,00
38,72	34,52	32,34	40,00	40,00	40,00	27,69	31,26	40,00	40,00
40,00	30,85	31,36	40,00	40,00	40,00	29,98	33,59	40,00	40,00
40,00	31,53	40,00	40,00	40,00	37,73	30,24	33,36	40,00	40,00
34,28	30,64	31,97	40,00	40,00	40,00	26,73	34,76	40,00	40,00
31,76	25,89	28,86	40,00	40,00	40,00	24,46	31,24	40,00	40,00
33,56	27,70	28,45	40,00	40,00	40,00	24,49	34,42	40,00	40,00
34,68	30,65	31,40	40,00	40,00	40,00	27,12	40,00	40,00	40,00
40,00	29,45	32,31	40,00	40,00	40,00	28,06	33,59	40,00	40,00
36,79	27,61	32,43	40,00	40,00	40,00	26,47	31,97	40,00	40,00
40,00	28,23	40,00	40,00	40,00	40,00	29,55	35,43	40,00	40,00
38,98	33,00	31,28	40,00	40,00	40,00	29,12	40,00	40,00	40,00
40,00	29,19	32,41	40,00	40,00	40,00	29,44	32,95	40,00	40,00
40,00	33,25	31,79	40,00	40,00	40,00	28,60	40,00	40,00	40,00
40,00	36,64	40,00	40,00	40,00	40,00	29,80	40,00	40,00	40,00
40,00	31,02	32,85	40,00	40,00	38,89	28,07	32,25	40,00	40,00
40,00	29,99	40,00	40,00	40,00	40,00	34,26	32,93	40,00	40,00
40,00	30,52	31,91	40,00	40,00	40,00	29,96	36,13	40,00	40,00
40,00	28,88	32,36	40,00	40,00	40,00	29,61	31,14	40,00	40,00
40,00	27,73	32,05	40,00	40,00	40,00	27,93	32,97	40,00	40,00
40,00	27,31	31,59	40,00	40,00	40,00	27,38	31,22	40,00	40,00
40,00	26,84	30,29	40,00	40,00	40,00	26,51	30,90	40,00	40,00
40,00	29,03	40,00	40,00	40,00	40,00	29,89	31,65	40,00	40,00
40,00	26,81	30,25	40,00	40,00	35,66	27,63	32,10	40,00	40,00
40,00	26,97	32,14	40,00	40,00	40,00	27,70	31,81	40,00	40,00
40,00	28,61	32,43	40,00	40,00	40,00	29,64	31,31	40,00	40,00
40,00	27,52	31,49	40,00	40,00	33,59	27,48	32,19	40,00	40,00
38,75	26,40	30,75	40,00	40,00	40,00	26,05	30,94	40,00	40,00
40,00	26,62	31,04	36,19	40,00	40,00	25,39	30,47	40,00	40,00
36,69	27,13	29,20	40,00	40,00	34,19	25,72	30,42	40,00	40,00
40,00	27,22	31,80	40,00	40,00	40,00	26,72	32,17	40,00	40,00
40,00	29,00	33,27	40,00	40,00	40,00	29,71	31,67	40,00	40,00
36,20	27,71	31,40	40,00	40,00	40,00	28,11	30,40	40,00	40,00
40,00	28,58	30,23	40,00	40,00	40,00	28,53	32,53	40,00	40,00
40,00	27,71	34,73	40,00	40,00	40,00	28,94	32,94	40,00	40,00
40,00	27,50	30,79	40,00	40,00	40,00	27,09	31,98	40,00	40,00
40,00	28,01	30,17	40,00	40,00	40,00	27,25	31,48	40,00	40,00
34,69	27,93	33,19	40,00	40,00	40,00	28,80	30,78	40,00	40,00
40,00	28,13	33,15	40,00	40,00	40,00	30,20	32,31	40,00	40,00

hsa-miR-873	hsa-miR-874	hsa-miR-875-3p	hsa-miR-876-3p	hsa-miR-876-5p	hsa-miR-885-3p	hsa-miR-885-5p	hsa-miR-886-3p	hsa-miR-886-5p
40,00	33,66	40,00	40,00	40,00	40,00	29,18	40,00	31,80
40,00	40,00	40,00	40,00	40,00	40,00	33,78	34,21	40,00
40,00	40,00	40,00	40,00	40,00	40,00	28,06	34,17	32,21
40,00	40,00	40,00	40,00	40,00	40,00	34,92	40,00	29,78
40,00	40,00	40,00	40,00	40,00	40,00	30,03	40,00	30,73
40,00	40,00	40,00	40,00	40,00	40,00	30,75	40,00	31,81
40,00	40,00	40,00	40,00	40,00	40,00	31,09	40,00	32,50
40,00	40,00	40,00	40,00	40,00	40,00	28,85	32,66	29,36
40,00	40,00	40,00	40,00	40,00	40,00	29,40	31,94	28,38
40,00	40,00	40,00	40,00	40,00	40,00	29,44	34,06	28,59
40,00	40,00	40,00	40,00	40,00	40,00	31,21	40,00	30,21
40,00	40,00	40,00	40,00	40,00	40,00	28,55	40,00	31,34
40,00	40,00	40,00	40,00	40,00	40,00	28,73	40,00	30,36
40,00	40,00	40,00	40,00	40,00	40,00	27,99	32,76	31,45
40,00	40,00	40,00	40,00	40,00	40,00	34,53	40,00	31,25
40,00	40,00	40,00	40,00	40,00	40,00	29,92	40,00	30,66
40,00	40,00	40,00	40,00	40,00	40,00	40,00	34,06	30,50
40,00	40,00	40,00	40,00	40,00	40,00	32,35	40,00	34,02
40,00	40,00	40,00	40,00	40,00	40,00	30,63	40,00	31,51
40,00	40,00	40,00	40,00	40,00	40,00	31,99	40,00	32,09
40,00	40,00	40,00	40,00	40,00	40,00	30,08	40,00	32,99
40,00	40,00	40,00	40,00	40,00	40,00	28,21	31,88	31,71
40,00	40,00	40,00	40,00	40,00	40,00	28,07	40,00	30,90
40,00	40,00	40,00	40,00	40,00	40,00	27,77	32,68	30,54
40,00	33,31	40,00	40,00	40,00	40,00	27,69	38,24	30,97
40,00	34,80	40,00	40,00	40,00	40,00	28,14	33,77	40,00
40,00	32,79	40,00	40,00	40,00	40,00	26,27	40,00	30,80
40,00	40,00	40,00	40,00	40,00	40,00	30,80	40,00	30,30
40,00	31,31	40,00	40,00	40,00	40,00	31,24	40,00	34,12
40,00	40,00	40,00	40,00	40,00	40,00	27,55	40,00	32,70
40,00	40,00	40,00	40,00	40,00	40,00	27,50	40,00	30,63
40,00	27,54	40,00	40,00	40,00	40,00	30,58	33,14	29,87
40,00	40,00	40,00	36,72	40,00	40,00	28,74	32,86	31,07
40,00	40,00	40,00	40,00	40,00	40,00	28,14	33,08	29,45
40,00	40,00	40,00	40,00	40,00	40,00	28,92	33,31	33,48
40,00	35,37	40,00	40,00	40,00	40,00	30,21	34,60	33,09
40,00	40,00	40,00	40,00	40,00	40,00	29,13	33,35	31,11
40,00	40,00	40,00	40,00	40,00	40,00	27,91	33,07	31,21
40,00	31,32	40,00	40,00	40,00	40,00	25,49	32,17	29,63
40,00	34,13	40,00	40,00	40,00	40,00	28,93	32,08	29,13
40,00	40,00	40,00	40,00	40,00	40,00	29,06	34,71	33,07
40,00	33,72	40,00	40,00	40,00	40,00	29,12	32,43	33,16

hsa-miR-887	hsa-miR-888	hsa-miR-889	hsa-miR-890	hsa-miR-891a	hsa-miR-891b	hsa-miR-892a	hsa-miR-9	hsa-miR-92a	hsa-miR-95
40,00	40,00	33,48	40,00	40,00	40,00	40,00	40,00	25,82	40,00
40,00	40,00	40,00	40,00	40,00	40,00	40,00	40,00	27,58	40,00
40,00	40,00	32,99	40,00	40,00	40,00	40,00	40,00	24,92	40,00
40,00	40,00	40,00	40,00	40,00	40,00	40,00	32,85	22,90	40,00
40,00	40,00	40,00	40,00	40,00	40,00	40,00	40,00	24,00	33,31
40,00	40,00	34,91	40,00	40,00	40,00	40,00	40,00	24,40	40,00
40,00	40,00	33,11	40,00	40,00	40,00	40,00	40,00	25,58	32,51
40,00	40,00	32,37	40,00	40,00	40,00	40,00	40,00	21,47	40,00
40,00	40,00	28,47	40,00	40,00	40,00	40,00	34,67	19,83	38,68
40,00	40,00	31,17	40,00	40,00	40,00	40,00	40,00	20,02	31,76
40,00	40,00	35,19	40,00	40,00	40,00	40,00	40,00	21,92	40,00
40,00	40,00	32,31	40,00	40,00	40,00	40,00	40,00	23,53	33,33
40,00	40,00	31,39	40,00	40,00	40,00	40,00	40,00	21,28	34,67
40,00	40,00	40,00	40,00	40,00	40,00	40,00	40,00	22,08	32,09
40,00	40,00	34,51	40,00	40,00	40,00	40,00	40,00	24,13	40,00
40,00	40,00	40,00	40,00	40,00	40,00	40,00	40,00	23,38	31,62
40,00	40,00	40,00	40,00	40,00	40,00	40,00	40,00	23,93	40,00
40,00	40,00	40,00	40,00	40,00	40,00	40,00	40,00	24,60	40,00
40,00	40,00	40,00	40,00	40,00	40,00	40,00	40,00	23,45	40,00
40,00	40,00	40,00	40,00	40,00	40,00	40,00	40,00	24,14	31,65
40,00	40,00	33,15	40,00	40,00	40,00	40,00	40,00	24,67	40,00
40,00	40,00	36,32	40,00	40,00	40,00	40,00	39,37	22,50	32,56
40,00	40,00	40,00	40,00	40,00	40,00	40,00	40,00	21,98	34,20
40,00	40,00	36,68	40,00	40,00	40,00	40,00	37,06	21,38	34,90
40,00	40,00	33,01	40,00	40,00	40,00	40,00	39,33	21,98	32,09
40,00	40,00	35,01	40,00	40,00	40,00	40,00	40,00	23,13	31,07
40,00	40,00	33,77	40,00	40,00	40,00	40,00	36,65	20,91	31,72
40,00	40,00	31,98	40,00	40,00	40,00	40,00	36,82	21,89	34,43
40,00	40,00	32,23	40,00	40,00	40,00	40,00	40,00	23,04	40,00
40,00	40,00	33,14	40,00	40,00	40,00	40,00	37,14	23,25	40,00
40,00	40,00	31,08	40,00	40,00	40,00	40,00	35,73	20,99	33,47
40,00	40,00	31,30	40,00	40,00	40,00	40,00	40,00	21,92	31,73
40,00	40,00	29,62	40,00	36,34	40,00	40,00	36,44	21,99	40,00
40,00	40,00	32,39	40,00	37,41	40,00	40,00	38,99	21,77	34,81
40,00	40,00	34,78	40,00	40,00	40,00	40,00	40,00	22,97	40,00
40,00	40,00	31,61	40,00	40,00	40,00	40,00	40,00	21,63	34,70
40,00	40,00	37,23	40,00	40,00	40,00	40,00	40,00	22,35	34,53
40,00	40,00	35,40	40,00	40,00	40,00	40,00	40,00	22,29	33,05
40,00	40,00	34,98	40,00	40,00	40,00	40,00	40,00	20,97	40,00
40,00	40,00	30,98	40,00	40,00	40,00	40,00	40,00	21,63	33,51
40,00	40,00	32,19	40,00	40,00	40,00	40,00	40,00	22,15	37,56
40,00	40,00	40,00	40,00	40,00	40,00	40,00	40,00	23,40	34,56

hsa-miR-98	hsa-miR-99a	hsa-miR-99b	mmu-miR-124a	mmu-miR-129-3p	mmu-miR-134	mmu-miR-137	mmu-miR-140	mmu-miR-153
40,00	40,00	33,75	40,00	40,00	27,43	40,00	27,71	40,00
40,00	40,00	40,00	40,00	40,00	30,42	40,00	30,42	40,00
40,00	40,00	31,89	40,00	40,00	30,64	40,00	25,64	40,00
40,00	40,00	28,93	40,00	40,00	26,76	40,00	23,86	40,00
40,00	34,38	31,44	40,00	40,00	27,89	40,00	25,82	40,00
40,00	33,40	31,05	40,00	40,00	32,70	40,00	26,73	40,00
40,00	33,99	33,42	40,00	40,00	34,91	40,00	27,79	40,00
40,00	32,98	30,08	40,00	40,00	26,91	40,00	26,78	40,00
40,00	30,31	25,49	40,00	40,00	26,27	40,00	23,83	40,00
40,00	30,25	27,76	40,00	40,00	27,83	40,00	24,22	40,00
40,00	32,03	30,64	40,00	40,00	28,40	40,00	26,76	40,00
40,00	40,00	30,67	40,00	40,00	33,12	40,00	27,15	40,00
40,00	33,08	28,10	40,00	40,00	30,86	40,00	26,18	40,00
40,00	33,21	31,85	40,00	40,00	32,57	40,00	27,74	40,00
40,00	40,00	32,01	40,00	40,00	40,00	40,00	27,40	40,00
40,00	32,60	29,58	40,00	40,00	35,97	40,00	27,13	40,00
40,00	40,00	31,82	40,00	40,00	30,88	40,00	27,55	40,00
40,00	40,00	31,50	40,00	40,00	32,04	40,00	32,99	40,00
40,00	35,21	30,76	40,00	40,00	33,17	40,00	28,13	40,00
40,00	40,00	35,35	40,00	40,00	37,69	40,00	30,05	40,00
40,00	34,17	32,75	40,00	40,00	34,63	40,00	27,29	40,00
40,00	37,75	29,43	40,00	40,00	36,53	40,00	27,08	40,00
36,78	32,65	29,43	40,00	40,00	38,51	40,00	26,57	40,00
33,25	32,38	27,49	40,00	40,00	34,52	40,00	26,33	40,00
32,69	30,37	29,08	40,00	40,00	38,74	40,00	25,71	40,00
40,00	32,86	30,81	40,00	40,00	37,73	40,00	27,09	40,00
37,27	32,42	28,58	40,00	40,00	37,05	40,00	25,66	40,00
35,71	39,16	29,50	40,00	40,00	32,51	40,00	25,60	40,00
34,24	40,00	30,47	40,00	40,00	35,63	40,00	28,16	40,00
40,00	37,82	29,35	40,00	40,00	32,99	40,00	26,97	40,00
36,36	31,17	27,86	40,00	40,00	32,00	40,00	24,95	40,00
31,66	31,53	28,15	40,00	40,00	31,67	40,00	24,87	40,00
35,32	33,41	27,19	40,00	40,00	31,32	40,00	24,82	40,00
40,00	33,57	29,27	40,00	40,00	32,24	40,00	26,45	40,00
36,11	40,00	30,42	40,00	40,00	34,91	40,00	28,24	40,00
36,96	38,07	27,16	40,00	40,00	33,03	40,00	27,06	40,00
35,44	32,08	29,05	40,00	40,00	33,77	40,00	26,56	40,00
40,00	32,45	29,76	40,00	40,00	37,24	40,00	27,05	40,00
37,59	31,19	27,22	40,00	40,00	35,95	40,00	25,63	40,00
40,00	31,91	27,43	40,00	40,00	30,62	40,00	26,44	40,00
40,00	30,55	30,15	40,00	40,00	31,09	40,00	27,80	40,00
40,00	40,00	31,02	40,00	40,00	36,04	40,00	29,51	40,00

mmu-miR-187	mmu-miR-374-5p	mmu-miR-379	mmu-miR-451	mmu-miR-491	mmu-miR-495	mmu-miR-496	mmu-miR-499	mmu-miR-615
40,00	25,23	40,00	30,96	33,18	30,34	40,00	40,00	40,00
40,00	25,70	40,00	31,96	40,00	40,00	40,00	40,00	40,00
40,00	26,56	34,50	29,26	30,33	30,13	40,00	40,00	40,00
40,00	23,77	31,91	28,10	29,28	28,64	40,00	40,00	40,00
40,00	26,98	33,14	29,93	31,63	27,86	40,00	40,00	37,38
40,00	27,38	31,08	27,44	31,54	31,10	40,00	40,00	40,00
40,00	30,08	40,00	28,19	37,50	40,00	40,00	40,00	40,00
40,00	24,79	30,70	23,98	31,43	28,15	40,00	40,00	40,00
35,11	24,19	25,59	22,33	27,22	25,01	34,60	40,00	40,00
40,00	22,30	28,60	20,96	28,18	28,44	40,00	34,71	40,00
40,00	24,11	30,76	24,21	31,49	28,72	40,00	40,00	40,00
38,26	26,91	32,33	26,27	31,29	28,82	40,00	40,00	40,00
40,00	25,87	30,26	26,41	30,87	28,20	40,00	40,00	40,00
40,00	28,57	40,00	23,73	32,32	30,01	40,00	40,00	40,00
40,00	25,13	40,00	28,31	33,25	30,75	40,00	40,00	40,00
40,00	27,30	31,25	27,51	31,35	32,29	40,00	40,00	40,00
40,00	24,90	40,00	28,08	31,56	31,76	40,00	40,00	40,00
40,00	40,00	40,00	29,29	40,00	34,56	40,00	40,00	40,00
40,00	27,40	36,94	27,24	32,80	30,97	40,00	40,00	40,00
40,00	32,01	33,85	26,03	37,33	32,60	40,00	40,00	40,00
40,00	27,09	32,09	26,26	33,85	30,31	40,00	40,00	40,00
40,00	26,98	33,75	23,88	32,26	30,07	40,00	40,00	40,00
40,00	27,26	32,78	21,58	31,59	29,86	33,51	40,00	40,00
40,00	26,23	31,01	23,69	30,73	28,03	40,00	40,00	40,00
40,00	25,40	35,14	21,77	29,27	28,79	40,00	40,00	40,00
40,00	28,72	32,41	23,26	40,00	31,00	40,00	40,00	40,00
40,00	25,84	34,90	21,04	30,77	28,19	37,61	40,00	31,07
40,00	26,67	33,69	23,40	31,95	28,25	40,00	40,00	40,00
40,00	29,04	31,93	27,42	30,96	29,22	40,00	40,00	40,00
40,00	26,32	31,15	24,96	30,98	29,03	40,00	40,00	40,00
40,00	24,58	29,88	22,88	29,65	27,06	34,21	40,00	40,00
40,00	24,48	32,57	22,02	28,69	28,09	34,22	40,00	40,00
37,47	24,04	28,99	24,15	28,99	26,39	34,87	40,00	40,00
40,00	26,61	31,33	22,64	30,25	27,28	40,00	40,00	40,00
40,00	30,40	40,00	23,96	33,63	30,42	40,00	40,00	40,00
40,00	27,59	31,37	24,56	31,34	26,94	33,65	40,00	40,00
40,00	26,93	40,00	25,05	30,94	30,00	40,00	40,00	40,00
40,00	27,75	40,00	23,30	31,35	30,22	40,00	40,00	40,00
40,00	25,66	40,00	23,00	29,93	29,29	40,00	40,00	40,00
40,00	26,80	33,24	25,30	30,05	27,52	40,00	40,00	40,00
40,00	28,01	40,00	22,13	32,17	29,25	40,00	40,00	40,00
40,00	31,15	40,00	24,96	40,00	30,61	40,00	40,00	40,00

mmu-miR-93	mmu-miR-96	RNU44	RNU48	U6 snRNA
27,18	40,00	40,00	40,00	30,34
29,83	40,00	40,00	40,00	29,96
25,88	40,00	40,00	40,00	27,63
23,79	40,00	40,00	33,62	26,81
24,92	40,00	40,00	34,83	28,99
25,73	40,00	40,00	40,00	27,22
26,76	40,00	40,00	40,00	30,45
24,49	40,00	40,00	34,03	29,34
21,86	40,00	40,00	33,25	27,86
21,73	40,00	40,00	40,00	24,29
24,27	40,00	40,00	40,00	30,52
25,60	40,00	40,00	40,00	29,90
23,51	40,00	40,00	40,00	29,41
24,18	40,00	40,00	34,88	30,83
26,44	40,00	40,00	40,00	31,48
25,72	40,00	40,00	40,00	30,95
26,61	40,00	40,00	40,00	30,93
28,25	40,00	40,00	40,00	29,44
26,34	40,00	40,00	40,00	29,30
27,67	40,00	40,00	40,00	30,03
26,60	40,00	40,00	40,00	31,19
24,62	40,00	40,00	34,69	30,14
24,09	40,00	40,00	33,66	29,09
24,07	40,00	40,00	40,00	28,83
23,60	40,00	40,00	35,58	29,04
25,64	40,00	40,00	33,10	29,92
23,14	40,00	40,00	40,00	28,70
23,68	40,00	40,00	40,00	26,24
26,28	40,00	40,00	40,00	30,25
25,30	40,00	40,00	40,00	31,50
23,25	40,00	40,00	34,62	30,37
22,88	40,00	40,00	40,00	29,85
23,32	40,00	40,00	35,92	30,22
23,81	40,00	40,00	40,00	28,63
25,36	40,00	40,00	40,00	30,74
24,20	40,00	40,00	40,00	30,72
24,82	40,00	40,00	40,00	30,95
25,57	40,00	40,00	35,98	30,03
23,77	40,00	40,00	35,71	29,16
24,28	40,00	40,00	35,00	29,48
24,76	40,00	40,00	40,00	30,68
26,34	40,00	40,00	40,00	30,49

Additional File 7 B. Raw data of biomarker discovery utilizing commercial TaqMan microRNA Assays.

Age	Gender	Smoking status	Study group	Histological subtyp	miR-24	miR-28-3p	miR-132-3p	miR-146b-5p
54	Male	Ever	Mesothelioma	Biphasic	21,50	26,99	29,98	26,75
72	Male	Ever	Mesothelioma	Biphasic	24,97	28,95	33,84	29,49
68	Male	Never	Mesothelioma	Epithelioid	21,43	26,25	28,72	25,38
34	Male	Ever	Mesothelioma	Epithelioid	19,34	23,86	27,00	23,22
70	Male	Never	Mesothelioma	Biphasic	21,39	25,31	28,61	25,32
59	Male	Never	Mesothelioma	Epithelioid	22,10	25,96	29,03	25,74
74	Male	Never	Mesothelioma	Epithelioid	23,65	27,94	30,66	27,61
73	Male	Never	Mesothelioma	Epithelioid	21,43	25,04	27,18	25,17
72	Male	Never	Mesothelioma	Sarcomatoid	19,28	23,22	24,76	22,21
66	Male	Ever	Mesothelioma	Epithelioid	19,12	23,11	25,38	22,58
73	Male	Ever	Mesothelioma	Biphasic	21,49	25,95	27,31	25,42
53	Male	Never	Mesothelioma	Epithelioid	22,63	25,86	29,11	25,98
56	Male	Ever	Mesothelioma	Epithelioid	20,63	24,92	27,30	24,36
77	Male	Ever	Mesothelioma	Epithelioid	22,14	25,99	26,76	25,55
84	Male	Never	Mesothelioma	Epithelioid	22,72	27,00	31,16	26,45
76	Male	Ever	Mesothelioma	Sarcomatoid	22,66	26,95	29,53	26,15
72	Male	Ever	Mesothelioma	Epithelioid	22,86	26,95	30,71	25,99
77	Male	Ever	Mesothelioma	Sarcomatoid	25,34	29,35	35,00	28,26
85	Male	Ever	Mesothelioma	Epithelioid	23,33	27,05	31,72	25,93
78	Male	Never	Mesothelioma	Epithelioid	25,77	28,98	32,13	27,60
68	Male	Ever	Mesothelioma	Epithelioid	24,02	27,89	31,22	27,23
67	Male	Ever	Asbestos-exposed control		21,42	26,25	27,21	25,96
71	Male	Ever	Asbestos-exposed control		21,90	26,70	28,38	26,39
79	Male	Ever	Asbestos-exposed control		20,24	25,45	26,31	24,74
72	Male	Never	Asbestos-exposed control		20,00	25,30	27,04	25,25
77	Male	Ever	Asbestos-exposed control		22,29	28,31	28,60	27,02
68	Male	Ever	Asbestos-exposed control		20,56	25,95	26,96	24,89
79	Male	Never	Asbestos-exposed control		20,08	25,56	26,26	25,04
71	Male	Never	Asbestos-exposed control		22,36	27,07	29,77	26,89
75	Male	Never	Asbestos-exposed control		20,54	25,43	27,54	25,16
43	Male	Ever	Asbestos-exposed control		20,04	24,36	26,39	24,82
78	Male	Ever	Asbestos-exposed control		19,37	24,27	25,69	23,71
72	Male	Ever	Asbestos-exposed control		19,05	24,65	26,71	24,41
72	Male	Never	Asbestos-exposed control		19,20	23,86	25,94	23,51
57	Male	Ever	Asbestos-exposed control		20,06	25,06	26,75	24,58
53	Male	Never	Asbestos-exposed control		22,25	27,66	29,75	27,48
69	Male	Never	Asbestos-exposed control		20,85	25,85	27,17	25,29
57	Male	Never	Asbestos-exposed control		20,52	25,34	26,78	25,16
81	Male	Ever	Asbestos-exposed control		22,12	26,97	28,02	26,35
75	Male	Ever	Asbestos-exposed control		19,80	24,62	26,15	24,01
55	Male	Ever	Asbestos-exposed control		21,95	26,79	28,54	26,18
82	Male	Never	Asbestos-exposed control		22,84	26,93	28,36	27,34

Additional File 7 C. Raw data of biomarker verification utilizing commercial TaqMan microRNA Assays.

Age	Gender	Smoking status	Study group	Histological subtyp	miR-16	miR-24	miR-28-3p	miR-126	miR-132-3p	miR-146b-5p	miR-625-3p	U6-snRNA
85	Male	Ever	Mesothelioma	Biphasic	23,14	23,61	29,31	27,40	31,71	26,06	30,09	29,86
73	Male	Ever	Mesothelioma	not available	20,96	21,20	26,16	24,95	28,56	25,24	27,06	27,48
68	Male	Never	Mesothelioma	not available	20,25	21,26	26,92	23,74	28,99	24,43	28,46	30,81
71	Male	Never	Mesothelioma	not available	22,77	25,15	31,04	27,05	31,92	29,09	32,19	27,07
51	Male	not available	Mesothelioma	Epithelioid	20,08	21,27	27,02	26,74	28,77	24,81	29,10	29,96
71	Male	Ever	Mesothelioma	Epithelioid	21,54	22,70	28,26	27,71	30,43	26,02	27,76	29,69
53	Male	Never	Mesothelioma	Epithelioid	21,72	21,84	27,94	25,19	29,57	24,73	27,69	30,40
55	Male	Ever	Mesothelioma	Epithelioid	22,56	22,29	28,93	27,13	30,82	24,71	29,35	32,19
75	Male	Never	Mesothelioma	Sarcomatoid	22,14	19,62	24,41	27,46	26,79	23,21	28,25	28,11
71	Male	Never	Mesothelioma	Epithelioid	21,93	19,67	24,78	27,55	27,33	23,57	30,49	30,00
67	Male	not available	Mesothelioma	Epithelioid	19,75	19,86	24,68	24,17	26,99	22,79	28,33	28,29
76	Male	Never	Mesothelioma	Epithelioid	18,89	18,71	24,25	27,49	25,77	22,42	25,96	28,40
72	Male	Never	Mesothelioma	Epithelioid	20,12	20,19	25,28	24,48	27,56	23,43	28,36	29,37
76	Male	Ever	Mesothelioma	not available	18,39	19,74	24,57	23,27	27,49	23,56	25,47	27,81
76	Male	Never	Mesothelioma	Epithelioid	20,13	23,17	28,06	26,49	30,01	26,45	29,14	30,77
75	Male	Ever	Mesothelioma	Epithelioid	18,26	18,43	23,22	27,10	25,72	22,05	26,44	25,54
77	Male	Never	Mesothelioma	Epithelioid	18,38	20,06	25,90	27,91	28,63	25,17	31,63	29,73
67	Male	Ever	Mesothelioma	Epithelioid	21,08	22,13	25,99	27,56	29,21	26,29	28,99	26,88
72	Male	Ever	Mesothelioma	Biphasic	19,44	20,22	25,12	31,82	27,76	24,42	28,99	28,51
75	Male	Never	Mesothelioma	Epithelioid	20,23	20,85	26,10	28,69	28,25	24,87	28,05	30,30
62	Male	Ever	Mesothelioma	Epithelioid	17,02	16,97	21,69	24,83	24,39	20,40	24,16	28,36
39	Male	Never	Mesothelioma	Sarcomatoid	16,95	19,07	24,73	35,00	26,84	24,19	24,80	29,69
55	Male	Ever	Asbestos-exposed control		27,35	24,86	27,79	27,61	30,77	28,87	34,30	34,82
57	Male	Ever	Asbestos-exposed control		22,98	22,13	25,17	23,84	28,66	25,94	28,71	32,85
55	Male	Ever	Asbestos-exposed control		21,35	22,39	25,33	23,17	28,41	24,86	30,99	31,03
59	Male	Never	Asbestos-exposed control		24,84	25,82	29,04	26,99	31,22	27,41	33,16	32,92
53	Male	Never	Asbestos-exposed control		26,42	24,88	26,40	27,09	29,65	26,91	34,51	32,09
68	Male	Never	Asbestos-exposed control		25,74	24,85	26,65	27,43	30,32	26,92	34,24	29,92
71	Male	Never	Asbestos-exposed control		22,42	23,46	27,21	24,69	30,11	24,74	32,54	34,30
73	Male	Ever	Asbestos-exposed control		24,85	25,47	28,14	26,96	31,63	27,61	32,05	33,22
75	Male	Ever	Asbestos-exposed control		31,96	24,02	29,19	31,89	31,06	29,10	34,96	32,67
77	Male	Never	Asbestos-exposed control		24,66	22,14	27,99	26,38	30,02	26,95	31,44	31,33
71	Male	Ever	Asbestos-exposed control		26,12	24,10	27,21	27,31	29,90	26,53	33,17	31,16
62	Male	Ever	Asbestos-exposed control		27,75	21,65	27,10	28,23	29,56	26,08	30,95	33,48
75	Male	Never	Asbestos-exposed control		21,19	21,92	26,02	23,26	28,46	24,45	30,44	33,86
76	Male	Never	Asbestos-exposed control		19,31	20,46	26,04	21,96	28,17	25,16	28,82	32,57
67	Male	Ever	Asbestos-exposed control		21,87	21,71	27,53	24,73	29,38	26,56	30,26	30,11
76	Male	Never	Asbestos-exposed control		35,00	23,01	28,46	35,00	29,77	25,55	35,00	35,00
67	Male	not available	Asbestos-exposed control		20,87	20,71	28,15	23,34	28,56	25,39	29,34	32,93
71	Male	Never	Asbestos-exposed control		29,63	24,44	26,31	28,98	28,67	25,83	33,99	33,35
77	Male	Never	Asbestos-exposed control		24,11	21,97	26,93	25,47	29,56	26,10	31,40	32,20
73	Male	Ever	Asbestos-exposed control		22,86	24,36	27,79	26,45	29,77	26,76	32,76	30,50
71	Male	Ever	Asbestos-exposed control		23,44	22,15	24,72	24,70	28,10	24,91	31,18	31,60
85	Male	Ever	Asbestos-exposed control		20,95	21,34	24,72	23,11	27,72	24,03	28,15	29,77
75	Male	Never	Asbestos-exposed control		25,27	24,57	26,92	26,96	29,68	26,44	32,29	31,85
62	Male	Ever	Asbestos-exposed control		24,73	22,15	27,66	26,20	29,42	26,20	30,95	31,37
76	Male	Ever	Asbestos-exposed control		20,44	20,89	27,24	23,09	28,22	25,00	30,08	31,09
67	Male	not available	Asbestos-exposed control		24,45	23,02	28,71	26,43	30,51	27,11	35,00	32,40
75	Male	Ever	Asbestos-exposed control		35,00	27,36	30,02	35,00	34,20	35,00	35,00	35,00
67	Male	Ever	Asbestos-exposed control		24,59	21,72	27,09	25,87	29,16	26,24	32,29	26,55
76	Male	Never	Asbestos-exposed control		33,96	24,39	29,23	34,46	31,37	28,58	35,44	31,85
68	Male	Never	Asbestos-exposed control		26,41	25,50	27,63	27,97	30,46	27,22	32,72	34,79
61	Male	Never	Asbestos-exposed control		23,90	24,07	27,29	26,17	29,60	26,30	32,44	31,27
71	Male	Never	Asbestos-exposed control		32,44	24,07	27,91	26,34	29,56	27,05	34,32	32,60
85	Male	Ever	Asbestos-exposed control		25,87	24,09	29,70	27,36	31,02	26,15	33,90	34,20
71	Male	Never	Asbestos-exposed control		23,34	27,70	27,40	31,99	30,05	29,60	35,00	34,21
61	Male	Never	Asbestos-exposed control		24,71	25,22	28,80	27,24	30,60	27,60	35,00	31,11
76	Male	Ever	Asbestos-exposed control		24,52	22,25	27,96	26,94	29,37	26,27	31,57	32,69
49	Male	Ever	Asbestos-exposed control		20,88	22,46	27,19	23,59	29,12	25,14	29,30	32,71
72	Male	Never	Asbestos-exposed control		26,11	24,87	30,19	28,48	31,30	27,92	33,89	32,34
75	Male	Never	Asbestos-exposed control		35,00	24,35	29,47	35,00	33,05	35,00	35,00	33,18
72	Male	Ever	Asbestos-exposed control		26,33	22,62	28,26	27,69	29,73	27,33	33,84	33,74
72	Male	Ever	Asbestos-exposed control		30,96	22,36	27,32	29,32	29,77	25,88	33,60	30,05
76	Male	Never	Asbestos-exposed control		27,69	22,42	26,72	28,40	29,68	26,17	32,02	30,70
75	Male	Never	Asbestos-exposed control		26,97	20,05	26,13	27,53	28,44	25,58	31,38	31,73
72	Male	Never	Asbestos-exposed control		27,17	23,94	29,05	29,03	30,59	28,20	34,15	31,03

Additional File 7D. Raw data of miRNAs in plasma samples spiked-in with lysed erythrocytes.

miRNA	Subject	Grade of hemolysis (%)					
		**0**	**0.125**	**0.25**	**0.5**	**1**	**2**
miR-20b	1	26,42	27,41	25,78	24,41	23,52	22,09
	2	25,22	24,13	24,34	22,93	21,50	20,45
	3	29,36	27,78	26,47	25,98	25,00	22,15
miR-24	1	21,58	23,30	22,23	21,70	21,95	21,13
	2	20,51	22,91	22,69	22,31	21,83	21,74
	3	24,53	24,05	24,21	24,12	23,50	22,11
miR-28-3p	1	25,09	26,73	26,39	26,24	26,90	25,17
	2	25,24	27,26	27,99	27,83	26,48	26,77
	3	29,19	30,03	29,55	29,17	28,23	27,27
miR-132-3p	1	28,36	28,61	28,92	28,11	29,24	27,34
	2	28,97	30,69	30,06	29,25	27,99	27,26
	3	29,08	29,09	29,58	28,91	28,53	28,95
miR-140-3p	1	26,34	26,14	25,16	25,23	24,42	23,09
	2	27,18	26,02	26,75	25,97	23,99	22,50
	3	28,19	27,31	26,22	26,01	23,70	23,68
miR-146b	1	25,03	26,94	25,76	25,25	25,43	24,53
	2	23,11	25,31	25,39	25,24	24,25	23,89
	3	29,24	29,70	29,18	29,36	28,56	26,22
miR-155	1	27,63	28,62	28,43	28,28	29,16	27,15
	2	27,01	29,45	29,48	29,12	28,82	28,54
	3	30,14	30,14	29,58	29,33	28,43	27,16
miR-191	1	22,70	23,29	22,71	22,04	21,49	20,67
	2	20,79	22,63	22,23	21,46	20,44	19,71
	3	25,84	25,08	24,84	24,49	23,50	21,05
miR-193a-5p	1	33,65	33,96	34,70	33,65	33,09	32,51
	2	28,56	28,57	28,66	28,59	28,31	27,89
	3	33,00	32,80	33,48	33,52	31,94	32,29
miR-328	1	30,00	32,07	31,29	29,96	30,65	28,88
	2	27,44	29,52	29,75	29,15	28,29	27,99
	3	34,58	34,07	33,50	32,89	32,26	31,07
miR-331	1	27,19	28,31	27,71	27,22	26,09	24,70
	2	25,85	26,45	27,33	26,01	24,23	23,73
	3	30,15	30,85	29,72	28,93	27,66	25,37
miR-381	1	31,18	31,53	31,21	31,94	31,98	31,19
	2	31,65	33,18	34,29	33,36	33,50	34,33
	3	31,44	32,75	31,93	31,31	31,29	31,20
miR-532	1	27,84	28,88	27,52	26,58	25,70	24,12
	2	27,47	26,72	27,10	26,16	24,71	23,70
	3	30,94	30,16	29,59	28,71	26,90	25,10
miR-628-5p	1	39,97	40,00	40,00	38,13	37,79	36,25
	2	33,07	35,39	35,80	34,50	32,38	32,91
	3	40,00	40,00	40,00	39,68	40,00	37,44
miR-660	1	25,50	25,50	24,54	24,05	22,90	21,63
	2	26,49	24,62	25,25	24,38	22,51	21,95
	3	26,78	25,92	24,93	23,91	23,06	22,15
